# Lipid-manager autophagy proteins ATG2 and ATG9 regulate extracellular vesicle secretion via amphisome biogenesis and cell lipidome modulation

**DOI:** 10.1038/s41467-026-75742-x

**Published:** 2026-07-22

**Authors:** Asmita Singh, Dylan J. Johnson, Jeffrey Kuhn, Katina L. Johnson, Jason G. Williams, Ricardo Vancini, Deloris Sutton, Kedar Sharma, Jadranka Loncarek, Mario J. Borgnia, Carlos M. Guardia

**Affiliations:** 1 Placental Cell Biology Group, National Institute of Environmental Health Sciences, National Institutes of Health, Durham, North Carolina USA; 2 Integrative Bioinformatics Support Group, National Institute of Environmental Health Sciences, National Institutes of Health, Durham, North Carolina USA; 3 Mass Spectrometry Research Center, National Institute of Environmental Health Sciences, National Institutes of Health, Durham, North Carolina USA; 4 Pathology Support Group, Division of Translational Toxicology, National Institute of Environmental Health Sciences, National Institutes of Health, Durham, North Carolina USA; 5 Molecular Microscopy Consortium, National Institute of Environmental Health Sciences, National Institutes of Health, Durham, North Carolina USA; 6 Cancer Innovation Laboratory, Center for Cancer Research, National Cancer Institute, National Institutes of Health, Frederick, Maryland USA

**Keywords:** Macroautophagy, Secretion, Membrane trafficking

## Abstract

Autophagy intersects with endocytic trafficking to regulate extracellular vesicle (EV) biogenesis, but how upstream lipid-handling autophagy proteins influence this crosstalk is unclear. Here we show that the autophagy lipid-supply proteins ATG9A and ATG2A/B restrain small EV (sEV) secretion by promoting amphisome formation and controlling cellular lipid composition. Deletion of ATG9A or ATG2A/B in cells, which abolishes autophagosome biogenesis, causes a RAB27A-dependent increase in secretion of CD63-enriched, smaller sEVs, and accumulation of intraluminal vesicles within multivesicular endosomes. Under lysosomal inhibition, wild-type cells release LC3- and autophagy cargo receptor-positive sEVs, whereas ATG9A- and ATG2A/B-deficient cells, despite hypersecretion of sEVs, fail to load LC3 or canonical cargo receptors, indicating a block in amphisome-mediated export. Proteomics reveals selective depletion of autophagy receptors and ferritinophagy factors and enrichment of RNA-binding proteins and endosomal trafficking regulators in sEVs from ATG9A- and ATG2A/B-deficient cells. Whole-cell lipidomics uncovers extensive rewiring of the lipidome, with accumulation of ceramides and neutral lipids, altered phospholipid balance, and transcriptional remodeling of lipid metabolic enzymes, while neutral sphingomyelinase inhibition normalizes sEV output. These findings identify ATG9A and ATG2A/B as lipid-dependent gatekeepers that couple autophagosome and amphisome formation, regulating membrane partition between degradative autophagy and exosome-mediated secretion.

## Abstract (web summary)

Singh et al. reveal that the lipid-handling autophagy proteins ATG9A and ATG2 regulate extracellular vesicle production by shaping cellular lipid composition and controlling whether cargo is degraded or released.

## Introduction

Macroautophagy (referred to as autophagy hereafter) is a conserved pathway of intracellular-content degradation and recycling^1,2^. Cells rely on autophagy to remove damaged organelles, protein aggregates, and invasive pathogens, in addition to controlling metabolic homeostasis during stressors such as hypoxia, starvation, and oxidative stress^3^. Proper execution of the autophagy cascade depends on the formation of the autophagosome, a unique double-membrane organelle that engulfs cytoplasmic content and fuses to endosomes from the endolysosomal system for breaking down its content and exporting the products back to the cytosol. The molecular machinery that coordinates the formation of the autophagosome requires the sequential activity and recruitment of several autophagy-related (ATG) gene products^4–6^. Upstream of these specialized ATG proteins, signaling is integrated and transduced into the activation of two major complexes: the ULK1/2 complex and the class III PI3K complex C1^4^. Together, these complexes will tether ATG9A-containing vesicles and induce the local production of PI3P, respectively, which in turn, recruits several effectors such as ATG2A and ATG2B (in complex with WIPI proteins) to assist in the delivery of phospholipids essential for the growth of the ATG9A-vesicles into a double-membrane cup-shaped phagophore^7–11^. These nascent autophagosomes get decorated with ubiquitin-like ATG8 family proteins such as LC3 through a conjugation machinery that covalently binds ATG8-like proteins to phosphatidylethanolamine (PE) in the autophagosome membrane^4^. The presence of lipid-conjugated ATG8-like molecules assures selective recruitment of cargo via interaction with cargo receptors like SQSTM1/p62, in addition to assisting in the closure of the autophagosome before fusion with endolysosomes^12,13^. This heterofusion event not only induces the degradation of the cargo but also regulates the formation of the amphisome, a hybrid organelle between multivesicular endosomes (MVEs) and autophagosomes.

Early cellular and biochemical characterization of amphisomes^14–17^ has shown that autophagosomes can fuse with early and late endosomes, accounting for a minor fraction of the most common autophagic vacuoles, the autolysosome^18^. But it has also been shown that treatment with leupeptin, a lysosomal protein degradation inhibitor, can induce a significant increase in the number of amphisomes^15^. The secretion of the amphisome content during prolonged impaired lysosomal inhibition has been recently termed SALI (secretory autophagy during lysosome inhibition)^19^, one of the many pathways every cell exploits to generate extracellular vesicles (EVs)^20^. The fusion of MVEs to the plasma membrane, releasing the intraluminal vesicles (ILVs) and their (cytosolic and membrane-bound) content to the extracellular medium, gives rise to a subpopulation of EVs called exosomes or small EVs (sEVs). These exosomes are small (40–150 nm in diameter) and contain several membrane proteins and lipids that are enriched in ILVs, such as the tetraspanin CD63 and sphingolipids, respectively^21^. In contrast, microvesicles are larger than 200 nm and, while their lumen also contains cytosolic material, they are generated by shedding from the plasma membrane^22^. They tend to be enriched in other proteins like Annexin A1^23^ and lipids such as ceramides and sphingomyelin^24^, but overlap of compositions between exosomes and microvesicles exists, given the nature of the endocytic and secretory pathways.

Together with the fact that induction of autophagy by starvation inhibits EV secretion^25^, cells tightly regulate cargo distribution between secretion and degradation. Remarkably, SALI seems to differ from other “non-canonical” autophagy pathways such as conjugation of ATG8 to single membranes (CASM)^26^, LC3-associated phagocytosis (LAP)^27^, LC3-associated endocytosis (LANDO)^28^, and LC3-dependent EV loading and secretion (LDELS)^29^, which specifically require the conjugation of ATG8 family of proteins to membranes other than the autophagosome. Accordingly, these processes recruit autophagic cargo to endosomes and ILVs, providing the cells with “non-canonical” ways of autophagic cargo degradation (microautophagy) or excretion through EVs. Significant progress in our understanding of CASM pinpoints to several perturbations that could uniquely activate SALI or CASM-related pathways: while vacuolar-type ATPase (v-ATPase) inhibitor Bafilomycin-A1 (BafA1) treatment (used in SALI) prevents both autophagosome degradation and CASM, lysosomotropic drug chloroquine (CQ) raises lysosomal pH, inhibiting autophagic flux, yet activates CASM^26^. Both SALI and CASM require certain autophagy machinery and converge at the MVE and amphisome level, with remarkable consequences for EV biogenesis, a crosstalk that has been poorly explored^30^.

Here, we employ a battery of conventional methods to isolate and characterize EVs from a selected combination of ATG knock-out (KO) cells and further define how SALI intersects with amphisome formation and EV secretion. We focus on ATG9A and ATG2A/B because these proteins supply and mobilize lipids during early autophagosome biogenesis, but may also influence membrane homeostasis through autophagy-independent roles at endosomes, lysosomes, lipid droplets, and the plasma membrane^31–34^. Our results show that loss of ATG9A or ATG2A/B produces two major cellular changes: increased secretion of CD63-enriched sEVs and drastic whole-cell lipidome remodeling. With more tetraspanin content and a complete change in protein and lipid content, increased secretion of EVs through classic pathways was observed. Comparison with ATG7 KO and FIP200 KO cells indicated that impaired autophagosome/amphisome formation contributes to defective export of LC3 and autophagy cargo receptors, whereas the enhanced EV output and lipid remodeling were most pronounced when the ATG9/ATG2 lipid-transfer axis is disrupted. Altogether, these findings provide a framework in which autophagy-dependent and -independent functions of lipid-handling ATG proteins coordinate membrane partitioning between degradative autophagy, amphisome-mediated cargo export, and MVE-dependent EV secretion.

## Results

### Autophagy-deficient cells differentially secrete sEVs

**Fig. 1:**
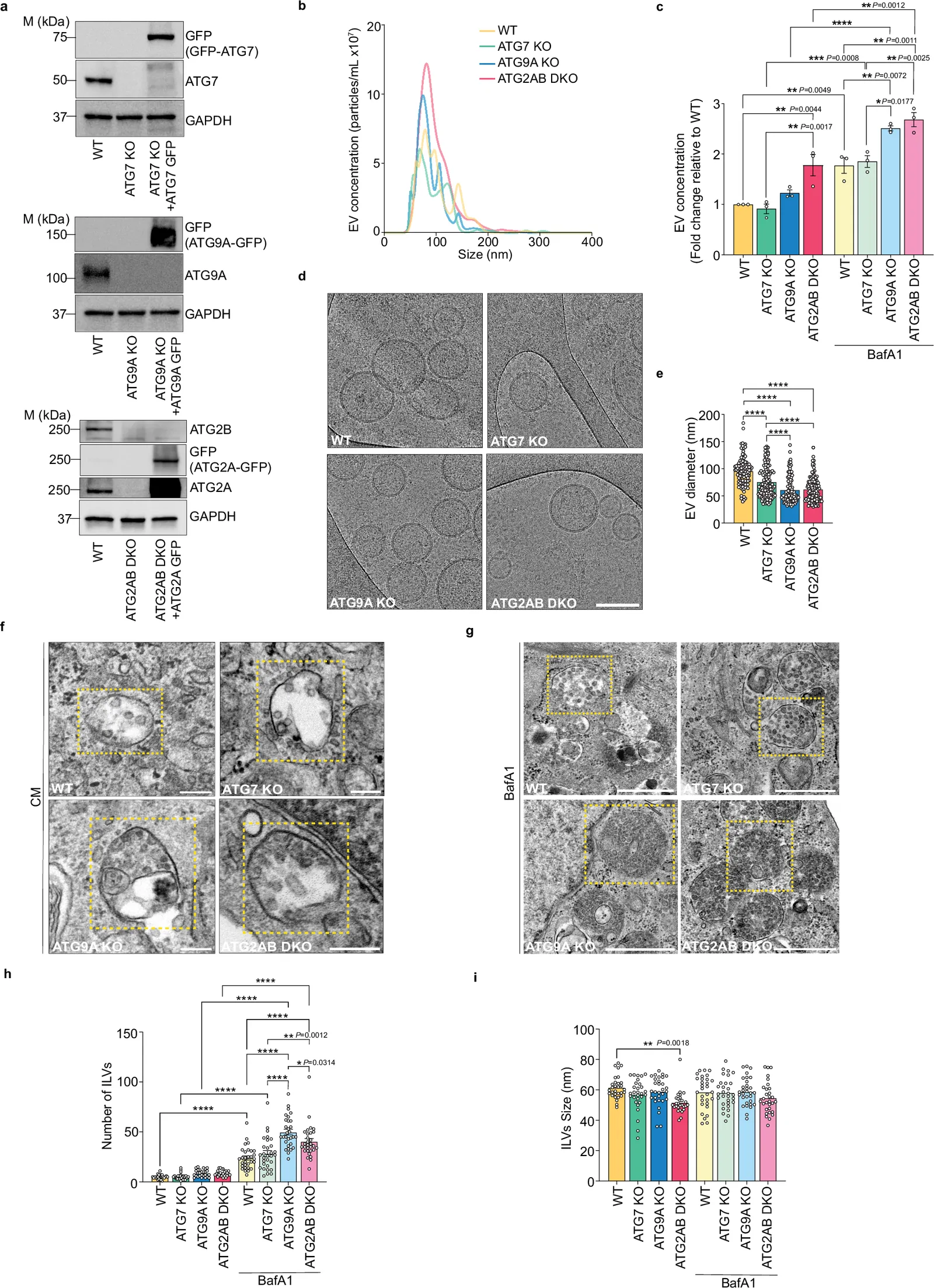
Autophagy-deficient cells differentially secrete EVs. **a** Immunoblot (IB) analysis of lysates from WT HeLa cells, cells with KO of ATG7 (ATG7 KO), ATG9A (ATG9A KO), and both ATG2A and ATG2B (ATG2AB DKO) and their corresponding rescued cells (ATG7 KO rescued with GFP-ATG7, ATG9A KO with ATG9A-GFP, and ATG2AB DKO with ATG2A-GFP), using antibodies as indicated on the right. The positions of molecular mass markers (in kDa) are indicated on the left. GAPDH was probed as a loading control. Representative image from *n* = 3 independent experiments. **b** Representative NTA profile of sEVs isolated from an equal number of cells using the isolation method described in the Materials and Methods section. **c** Quantification of total EV concentration from experiments such as those shown in (**b**). *n* = 3 biologically independent replicates in control medium and BafA1-treated cells. Data are plotted as mean ± SEM. Statistical significance was calculated using two-way analysis of variance (ANOVA) with Tukey’s multiple comparisons test. Only significant results are shown for clarity, * *P* < 0.05, ** *P* < 0.01, *** *P* < 0.001, **** *P* < 0.0001. **d** Representative cryo-electron microscopy images of EVs isolated from cells in control medium. Scale bar: 200 nm. **e** Quantification of particle size (diameter) from cryo-electron micrographs in (**d**). Data are plotted as mean ± SEM from *n* = 100 EV particles. Statistical significance was calculated using one-way ANOVA with Tukey’s multiple comparisons test, ****P < 0.0001. **f**,** g** Representative transmission electron micrographs of ultrathin sections from the indicated cells cultured either in control medium (**f**) or BafA1 (**g**). Yellow boxed regions indicate MVEs containing ILVs. Scale bar: 500 nm. **h**,** i** Quantification of the number and average size of ILVs (*n* = 25 MVEs) from images such as those shown in panels (**f**, **g**). Data are plotted as mean ± SEM. Statistical significance was calculated using two-way ANOVA with Tukey’s multiple comparisons test, and only significant results are shown: * *P* < 0.05, ** *P* < 0.01, *** *P* < 0.001, **** *P* < 0.0001.

To examine the impact of autophagy on EV release in HeLa cells, CRISPR-Cas9 was used to KO the two main ATG proteins involved in lipid management during autophagosome formation: ATG9A (ATG9A KO) and ATG2A/ATG2B (ATG2AB DKO) (Fig. 1a, Supplementary Information Table 1). These KO conditions rendered the cell’s ability to provide lipids for the growth of the phagophore membrane during early steps of autophagy^35^ and, potentially, to mobilize lipids in autophagy-independent manners as recently demonstrated^31–34^. ATG7 KO cells were also generated as a control of a downstream effector, inhibiting the LC3 lipidation on autophagosomes (and thus selective autophagy-related cargo handling) without blocking its formation^36,37^. Immunoblotting analysis confirmed successful KO in all generated lines (Fig. 1a). As expected, all these KO cells failed to show autophagic flux, which was rescued upon re-expression of the corresponding ATG proteins tagged with GFP (Fig. 1a, Supplementary Fig. 1a). To isolate sEVs secreted from these cells, conditioned media harvested from equal number of cells, cultured alone or in the presence of lysosomal acidification inhibitor BafA1 to trigger SALI, was recovered and a combined differential centrifugation with size exclusion chromatography (SEC) protocol was implemented (Material and Methods section). Under control-medium conditions, a significant increase in sEV concentration from ATG2AB DKO compared to WT cells was observed (Fig. 1b, c). This increase was much more pronounced in both ATG9A KO and ATG2AB DKO cells when lysosomes were inhibited, while ATG7 KO cells showed a similar pattern of secretion to WT cells (Fig. 1b, c). The increased secretion of sEVs from these KO cells was completely reversed by overexpression of the respective proteins (Supplementary Fig. 1b, c), demonstrating that the effects of the KOs on EV secretion were specific to the absence of the target ATG protein. Using cryogenic electron microscopy (cryo-EM)^38^, we observed that the diameter of the isolated sEVs from all ATG KO cells was significantly smaller compared to WT cells (Fig. 1d, e), showing a reduction in size of ~20% and 40% for ATG7 KO and both ATG9A KO and ATG2AB DKO sEVs, respectively. Given that the majority of sEVs originate from the fusion of MVEs to the plasma membrane^39^, we examined whether the morphology of MVEs was altered in ultrathin sections of the KO cells by transmission electron microscopy (TEM) experiments. We found that while ATG9A KO and ATG2AB DKO cells presented a trend towards MVEs with a greater number of ILVs in normal medium (Fig. 1f, h), the same cells treated with BafA1 showed a significant increase in MVE structures with many smaller ILVs (especially in ATG2AB DKO cells) (Fig. 1g-i). Altogether, these results suggested that the depletion of ATG9A or ATG2A/B increased the number of ILVs in MVEs, leading to enhanced secretion of smaller EVs in comparison to both WT and ATG7 KO cells.

### Autophagy knockout cells differentially regulate sEVs and secretory autophagy markers

**Fig. 2:**
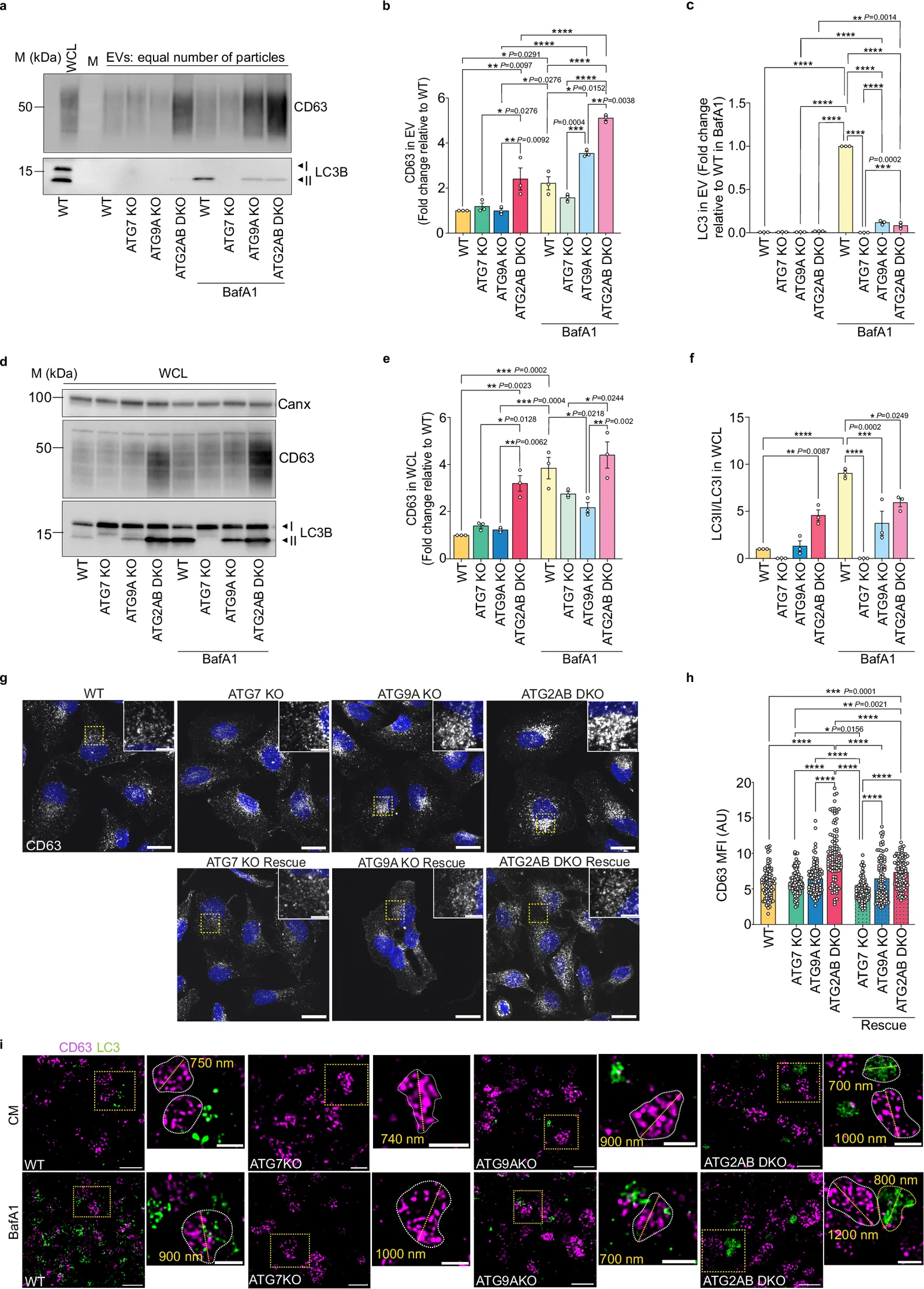
Intra and extracellular levels of CD63 are affected in ATG2AB but not ATG9A or ATG7 KO cells. **a** IB analysis of exosomal marker protein CD63 and secretory autophagy marker LC3B-II on an equal number of isolated EVs. The positions of molecular mass markers (M; second lane) (in kDa) are indicated on the left. The first lane contains the whole-cell lysate (WCL) from WT cells under BafA1 treatment conditions to corroborate LC3B-II migration in EV lanes. **b**,** c** Quantification of CD63 and LC3B-II in EVs from panel (**a**). Individual data points are marked as open circles from three independent biological replicates. Bars represent the mean ± SEM of fold change. Statistical significance was calculated using two-way ANOVA with Tukey’s multiple comparisons test, * *P* < 0.05, ** *P* < 0.01, *** *P* < 0.001, **** *P* < 0.0001. **d** IB analysis of CD63 and LC3B in WCL from WT and ATG KO cells under control or BafA1 treatment. The positions of molecular mass markers (M) (in kDa) are indicated on the left. **e**,** f** Quantification of cellular CD63 and LC3B-II/I levels as fold change relative to control WT cells from three independent experiments from panel (**d**). Bars represent the mean ± SEM. Statistical significance was calculated using two-way ANOVA with Tukey’s multiple comparisons test, * *P* < 0.05, ** *P* < 0.01, *** *P* < 0.001, **** *P* < 0.0001. **g** Representative confocal images of CD63 (gray) and DAPI (blue) in WT, ATG KO and rescued cells. Scale bar = 10 μm; inset image scale bar = 5 μm. **h** Quantification of the mean fluorescence intensities (MFI) of CD63 per cell from images shown in panel (**g**) and plotted as mean ± SEM from a total of 90 cells from three independent biological experiments. Statistical significance was calculated using two-way ANOVA with Tukey’s multiple comparisons test, * *P* < 0.05, ** *P* < 0.01, *** *P* < 0.001, **** *P* < 0.0001. **i** Representative deconvoluted STED images of endogenous CD63 and LC3B staining in WT and KO cells cultured in CM conditions from *n* = 3 independent biological replicates. Magnifications of the yellow-boxed regions are shown to the right. Scale bar = 1 µm; inset image scale bar = 0.5 µm.

The protocol of EV isolation used here yields sEVs that arise from different intracellular compartments^40^. To investigate the cargo composition of these EVs, we analyzed the most enriched tetraspanin marker CD63 in exosomes^41,42^ and LC3B, as a marker of SALI^19^, by immunoblotting. Analysis of sEVs released from WT and ATG KO cells showed that ATG2AB DKO-derived EVs contained more CD63 relative to those from WT cells under control conditions (Fig. 2a). A similar pattern of detection was observed for syntenin-1^43^, indicating an enrichment of exosomes on these preparations (Supplementary Fig. 2a, b). We then observed that under BafA1 treatment conditions, much more CD63 was present in the EVs released from ATG9A KO and ATG2AB DKO cells (Fig. 2a, b, Supplementary Fig. 2a, b). All preparations were microsome-free (calnexin-negative), but ectosomal proteins such as CD98 and CD147^39^ were detected in BafA1-treated cells (Supplementary Fig. 2a, b). This increase in CD63 levels per EV was a specific consequence of the ablation of these ATG proteins, given the reversal of the phenotype observed in the rescued cells (Supplementary Fig. 2c, d, f, g). Remarkably, the same alteration in levels of CD63 was found in the whole-cell lysates (WCL) from ATG2AB DKO cells (Fig. 2d, e). Immunofluorescence and flow cytometry analysis also showed increased CD63 signal intensity in ATG2AB DKO cells, which exhibited reversal in phenotype when rescued by overexpression of GFP-ATG2A (Fig. 2g, h, Supplementary Fig. 2i). These findings demonstrate that CD63 levels in sEVs are significantly affected in ATG2AB DKO cells (and ATG9A KO to a lesser degree) under both control and BafA1 when compared to WT and ATG7 KO cells, and that this could be a consequence of increased intracellular CD63 levels in the endolysosomal system.

We next examined sEV secretion in FIP200 KO cells, a subunit of the ULK1/2 complex, as a way to compare the effects of impaired autophagy initiation with loss of the lipid-transfer machinery (ATG9A KO and ATG2AB DKO) (Supplementary Fig. 4a). FIP200 KO cells showed autophagy flux deficiency with accumulation of LC3B and p62 (in ATG9A-positive vesicles) as shown by immunofluorescence and immunoblotting experiments (Supplementary Fig. 4b, d). Like ATG9A KO and ATG2AB DKO cells, FIP200 KO cells produced sEVs with reduced LC3B under BafA1 treatment, consistent with impaired autophagosome/amphisome-dependent export (Supplementary Fig. 4d). However, FIP200 KO cells did not accumulate lipid droplets by PLIN3 staining (Supplementary Fig. 4b) and did not show the increase in sEV particle number observed in ATG9A KO and ATG2AB DKO cells (Supplementary Fig. 4c), despite elevated CD63 levels in sEVs released under BafA1 treatment (Supplementary Fig. 4d). These results imply that while FIP200 loss recapitulates EV cargo changes linked to autophagy disruption, the enhanced EV secretion observed in ATG9A KO and ATG2AB DKO cells was not observed, likely reflecting additional autophagy-independent roles of these proteins in the phenotypes observed so far.

It has been proposed that sustained lysosomal inhibition or impaired autophagosome maturation during SALI induces the accumulation of amphisomes where autophagy and ILV cargo can mix and be released as EVs^19^. Thus, we next investigated whether we could observe the secretion of LC3B in the sEVs coming from our ATG-deficient cells. As expected, we did not observe significant LC3B secretion under control conditions from any of the cells (Fig. 2a, c, f, Supplementary Fig. 2e), though very low and variable amounts were observed in sEVs isolated from ATG2AB DKO cells. However, in BafA1-treated cells, LC3B secretion was clearly detected in sEVs from WT cells (Fig. 2a, c), and to a significantly lesser extent, in sEVs produced by ATG9A KO and ATG2AB DKO cells. The complete absence of the LC3B signal in sEVs from ATG7 KO cells in both conditions confirmed that the observed band corresponded to the lipidated form of LC3B (LC3B-II)^29^. Although shown in a different cell type, BafA1 treatment, and isolation conditions, EVs from ATG7 KO and ATG2AB DKO showed a complete absence of LC3B^19^, prompting us to interrogate where this LC3B-II comes from when ATG9A KO and ATG2AB DKO cells are unable to form autophagosomes and, thus, amphisomes. We hypothesized that this could be explained by basal CASM^26,44^. Treatment with either chloroquine (CQ), a CASM enhancer^45^, or overexpression of GFP-SopF, a CASM inhibitor^46^ did not show a particularly significant increase or decrease in the amount of LC3B-II in the EVs from the ATG9A KO or ATG2AB DKO cells, respectively (Supplementary Fig. 2j, k). Thus, these experiments suggest that the residual LC3B-II detected in CD63-rich sEVs from ATG9A KO and ATG2AB DKO cells is independent of amphisome-plasma membrane fusion and is not readily explained by CASM.

Finally, to evaluate how LC3B and CD63 behave inside these cells, we performed stimulated emission depletion (STED) super-resolution fluorescence microscopy experiments, under both control and BafA1-treated conditions (Fig. 2i, Supplementary Fig. 3). In WT cells, CD63 appeared as distinct clusters of small foci of around ~700 nm representing ILVs and MVEs. After treatment with BafA1, a significant increase of LC3-positive puncta in very close proximity to CD63 foci was observed suggesting the formation of amphisomes (Fig. 2i). As expected, lack of LC3B staining and structures in the ATG7 KO cells confirm the absence of membrane-associated LC3B in these cells (Fig. 2i). Interestingly, ATG9A KO and ATG2AB DKO cells presented with very large LC3-positive foci (~1000 nm) that resemble classic confocal and TEM experiments in these cells, where the lack of functional autophagosomes induces accumulation, aggregation, and segregation of LC3-rich structures in the cytoplasm^47,48^. The LC3-positive structures in ATG9A KO and ATG2AB DKO cells remained spatially segregated from CD63-positive compartments at the resolution of STED imaging, in contrast to the increased juxtaposition/overlap observed in BafA1-treated WT cells (Fig. 2i), suggesting that amphisome formation is effectively disrupted in these autophagosome-deficient cells.

Altogether, these results suggest that deficiency of ATG9A and ATG2A/B affects intracellular CD63 levels, impacting its content in ILVs and secreted sEVs, while simultaneously blocking the formation of amphisomes, which could explain the reduced abundance of LC3B and autophagy cargo in sEVs.

### Autophagy-deficient cells show enhanced fusion of CD63-positive MVEs with the plasma membrane

**Fig. 3:**
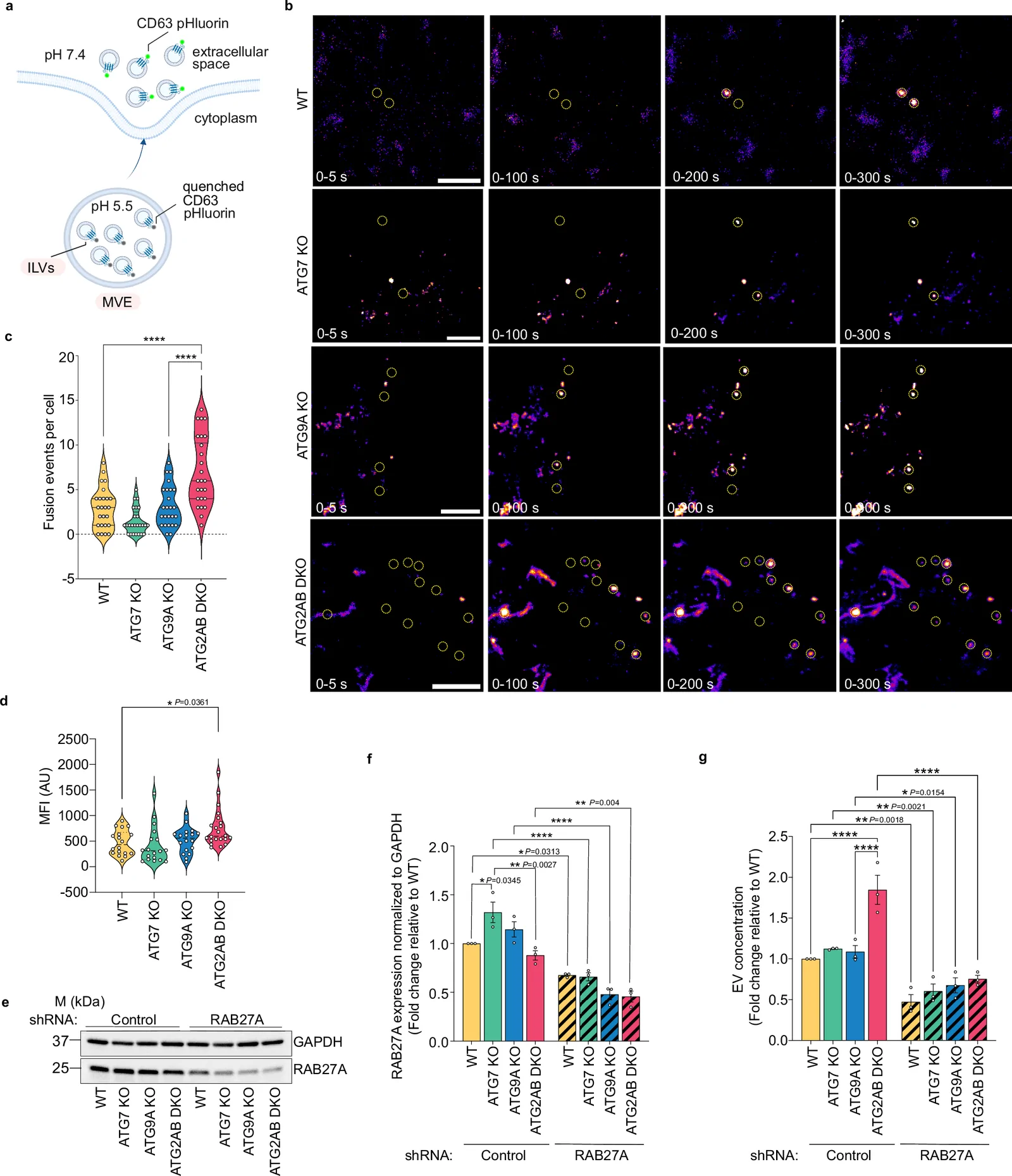
Secretion of EVs in autophagy-deficient cells is regulated by RAB27A. **a** Schematic representation of the CD63 pHluorin assay to visualize MVE-plasma membrane fusion^49^. Created in BioRender. Singh, A. (2026) https://BioRender.com/78ojc09 and modified in Adobe Illustrator. **b** Live-cell TIRF imaging of CD63-pHluorin expressing WT and ATG KO cells in control medium. Representative still images from Supplementary Movies 1–4, showing total cumulative fusion events over a time interval up to 300 s. Yellow circles represent single fusion events within the time-lapse. Scale bar = 20 µm; Supplementary Movies 1–4, Scale bar = 10 µm. **c** Quantification of fusion events per cell over 300 s for the data represented in panel (**b**). Statistical significance was calculated using one-way ANOVA with Tukey’s multiple comparisons test, **** *P* < 0.0001. **d** Quantification of fluorescence intensities of fusion events per cell for the data represented in panel (**c**). Statistical significance was calculated using one-way ANOVA with Tukey’s multiple comparisons test, **** *P* < 0.0001. **e** IB analysis of RAB27A KD compared to control cells (stably transfected with a scramble shRNA). The positions of molecular mass markers (in kDa) are indicated on the left. GAPDH was probed as a loading control. **f** Quantification of fold change in RAB27A expression levels (relative to WT in the control) normalized to GAPDH from three independent experiments, such as that shown in (**e**). Data are shown as mean ± SEM. Statistical significance was calculated using two-way ANOVA with Tukey’s multiple comparisons test, and only significant results are shown: * *P* < 0.05, ** *P* < 0.01, *** *P* < 0.001, **** *P* < 0.0001. **g** Quantification of total EV concentration derived from the indicated cells. *n* = 3 biologically independent replicates. Data are shown as mean ± SEM. Statistical significance was calculated using two-way ANOVA with Tukey’s multiple comparisons test, and only significant results are shown, * *P* < 0.05, ** *P* < 0.01, *** *P* < 0.001, **** *P* < 0.0001.

Given the higher amount of CD63 observed in sEVs released from ATG9A KO and ATG2AB DKO cells, we characterized whether this enhancement was indeed the result of enhanced MVEs fusing with the plasma membrane. To test this, we performed live cell total internal reflection (TIRF) microscopy in cells transfected with pH-sensitive pHluorin-tagged CD63, which remains quenched inside the acidic MVE compartment, but fluoresces only when MVEs fuse to the plasma membrane and are exposed to the neutral pH of the culture medium^49^ (Fig. 3a, b, Supplementary Movies 1–3). We observed a two-fold increase in fusion events in ATG2AB DKO cells compared to the other cell lines in complete medium (Fig. 3b, c, Supplementary Movie 4), along with increased fusion puncta intensities (Fig. 3d), suggesting a direct connection between EV concentration in the conditioned media and secretion from MVEs (Fig. 3b, c). Since RAB27A mediates docking and fusion to the plasma membrane^50^, we stably silenced RAB27A in WT and ATG KO cells to test whether the isolated EVs derive from this pathway of secretion. We confirmed the efficiency of RAB27A knockdown (KD) compared to empty vector control by immunoblot analysis (Fig. 3e, f, Supplementary Information Table 2). RAB27A KD in all the cells reduced the number of secreted EVs (Fig. 3g). Accordingly, these findings led us to conclude that RAB27A is involved in the increased secretion of sEVs observed in all the ATG KO cells.

### sEVs from ATG KO cells have different protein cargo composition

**Fig. 4:**
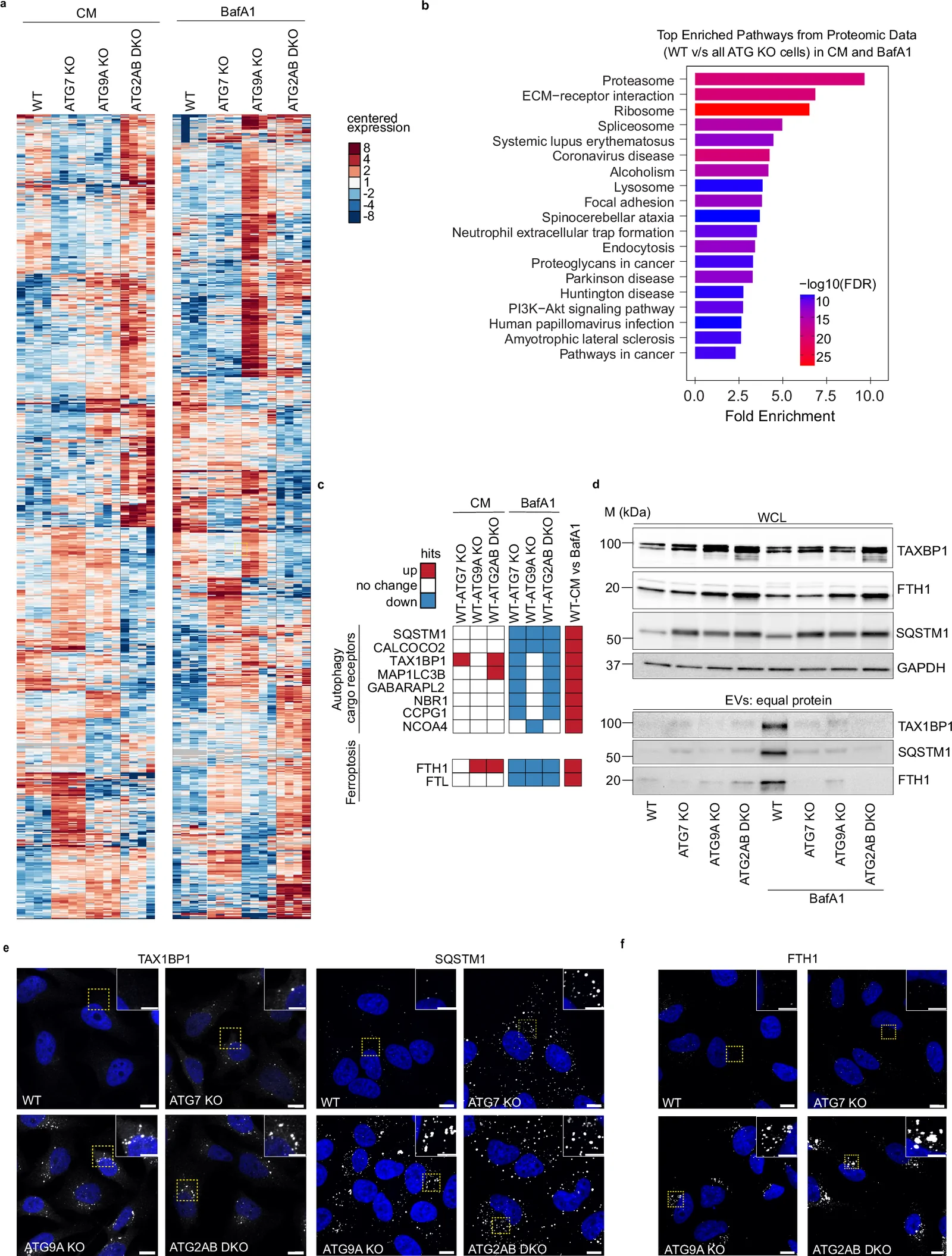
Autophagy-deficient cells secrete EVs with largely different protein composition. **a** Heat map (centered expression) depicting the differentially detected proteins in EVs isolated from WT and ATG KO cells in control medium (CM) or in the presence of BafA1. **b** Top enriched pathways from the proteome data shown in (a) (WT vs all ATG KO cells in CM and BafA1), ranked by enrichment of detected proteins in the pathway and colored by false discovery rate (FDR) using ShinyGO 0.82^121^. **c** Heat map depicting the differential abundance of autophagy cargo receptors and ferritinophagy-related proteins in EVs from ATG KO cells relative to WT cells under CM and BafA1-treated conditions. Significantly upregulated proteins are shown in red and downregulated proteins in blue. **d** IB validation of selected proteins, which are differentially abundant, as shown in panel (**c**), in both WCL and EVs. The positions of molecular mass (M) markers (in kDa) are indicated on the left. GAPDH was probed as a loading control. Results are representative of *n* = 2 independent biological replicates. **e**,** f** Representative confocal images of cargo receptors TAX1BP1 and SQSTM1 and ferroptosis-associated protein FTH1 (gray) and DAPI (blue) in WT and ATG KO cells in CM. Scale bar = 10 µm, inset image scale bar = 5 µm. Results are representative of *n* = 2 independent biological replicates.

How different are the sEVs secreted from these autophagy-deficient cells? In order to start answering this question, we performed proteomic analysis of EVs isolated from WT and KO cells under control and BafA1 treatment conditions. sEVs were isolated using EXODUS^51^ from four independent biological replicates, and total protein-normalized samples were processed for relative quantification using label-free mass spectrometry (Materials and Methods section). Inspection of the proteins identified from all the conditions (*n* = 1722) showed substantial overlap with the top 100 proteins enriched in EVs from databases such as ExoCarta^52^ and Vesiclepedia^53^ and more than 60 core proteins from MISEV guidelines^40^, confirming sEV identity from all the samples (Supplementary Fig. 5a, b). PCA analysis (Supplementary Fig. 5c) and centered expression heat maps for all the differentially detected proteins (Fig. 4a, Supplementary Data 1) revealed the separation of ATG KO from WT samples in both control and BafA1 conditions, suggesting global differences at the protein level of all the EVs. Remarkably, top enriched pathways from these differentially detected proteins included pathways related to cellular homeostasis, such as proteasome, focal adhesion, transcription regulation and translation, endolysosomal pathway, and autophagy (Fig. 4b).

In order to tease out relevant comparisons, volcano plots of enriched and depleted protein hits relative to WT sEVs for each condition were created (Supplementary Fig. 5d). ATG9A KO (total differentially detected hits = 215) and ATG2AB DKO (354) sEVs showed a much higher degree of changes than sEVs from ATG7 KO (193) when compared to the composition of EVs from WT cells from control medium (Supplementary Fig. 5d). Remarkably, upregulation of proteins in sEVs from KO cells compared to WT cells was the general trend driving the global differences (Supplementary Fig. 5d). Under BafA1 treatment, sEVs presented many more differentially secreted proteins in all the ATG-deficient cells compared to WT: ATG9A (277), ATG2AB (445), and ATG7 (237) (Supplementary Fig. 5d), showing a ~ 25% increase all across the samples, consistent with the observed increase in overall protein secretion (Fig. 2a, b). Remarkably, sEVs released from ATG7 KO cells under BafA1 treatment shared more differentially detected proteins with ATG2AB DKO sEVs than in control conditions (155 versus 63, a ~ 2.5-fold increase) while a similar comparison between ATG9A KO and ATG2AB DKO sEVs did not change significantly (120 versus 112, ~1.1-fold increase) (Supplementary Fig. 6a, b). This global analysis suggests a shift in sEV cargo composition from ATG7 KO cells towards a profile much more similar to ATG2AB DKO EVs than ATG9A KO, under the same lysosome-inhibited conditions, while in control medium conditions, only sEVs from ATG2AB DKO cells seem to be drastically different from the rest.

Interestingly, under SALI, sEVs released from WT cells showed remarkable enrichment of autophagosome cargo receptors (Supplementary Fig. 5e), as previously demonstrated^19^. This prompted us to interrogate the status of these specific proteins in the EVs isolated from the other generated cell lines. Analysis of proteins such as NBR1, TAX1BP1, SQSTM1, CALCOCO2, MAP1LC3B, and GABARAPL2 (Fig. 4c, Supplementary Data 2) showed no significant changes or minimal enrichment in the KOs relative to WT sEVs in control medium, but were significantly depleted under BafA1 treatment, confirming the requirement of active autophagy and formation of amphisomes for secretion in sEVs (Fig. 4c). Immunoblotting in whole-cell lysates (Fig. 4d) and immunofluorescence experiments of representative cargo receptors SQSTM1 and TAX1BP1 (Fig. 4e) confirmed accumulation of the proteins inside the ATG KO cells compared to WT. But more importantly, isolated sEVs from these cells presented very low levels of these cargo receptors (Fig. 4d) in control medium and accumulation only in WT cells under BafA1 treatment, which mimics the trends observed from the proteomics analysis (Fig. 4c). Interestingly, another group of proteins showed a similar pattern of depletion under SALI in the ATG KO cells. Those proteins were NCOA4, FTL, and FTH1, all involved in ferritinophagy, a form of autophagic ferroptosis^19,54^ (Fig. 4c). Validation of the proteomics data was performed by FTH1 immunoblotting and immunofluorescence experiments that corroborated the trends (Fig. 4c–e). This elevated basal FTH1 signal in sEVs from ATG9A KO and ATG2AB DKO cells may reflect the enhanced ILV production or microautophagy-like ferritin handling in these backgrounds^55,56^. Remarkably, several cargo proteins shown to be involved in RNA loading into EVs, such as YBX1^57^ and HNRNPK^58^, were upregulated in sEVs from ATG9A and ATG2AB DKO cells (Supplementary Fig. 6a, b). Especially in EVs isolated from ATG9A KO cells, we found a peculiar enrichment of proteins involved in the regulation of turnover of recycling endosomes, including RAB5B, RAB11, RAB13, RABAC1, RAB9A, SCAMP3, and TFRC (Supplementary Fig. 6a, b), which also confirmed an increase in vesicular trafficking and sorting upstream of MVE formation in this particular ATG KO cell line. Altogether, these results demonstrate that a loss in autophagosomes and ATG8-conjugation machinery in the cell globally affects sEV protein content and composition and selectively depletes autophagy cargo receptors from sEVs.

### Autophagy-deficient cells show whole-cell lipidome alterations that affect CD63 trafficking

**Fig. 5:**
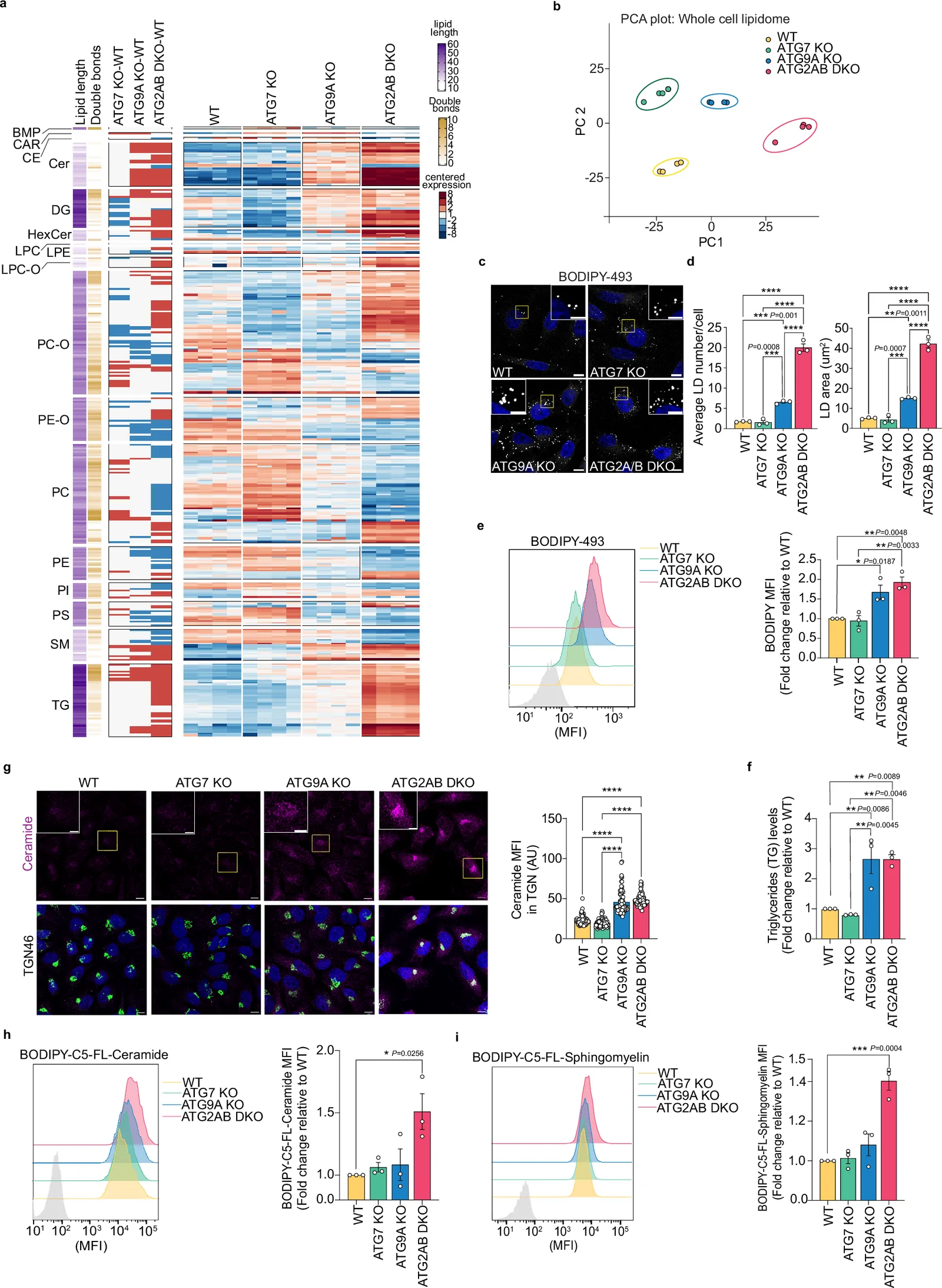
Autophagy-deficient cells show whole-cell lipidome alterations. **a** Heat map showing the differential lipid-species abundance in WT and ATG KO cells (*n* = 4 independent replicates). Each rectangle represents a lipid species, colored by centered abundance from blue (decreased) to red (increased): BMP, bis(monoacylglycero)phosphates; CAR, acylcarnitines; CE, cholesteryl esters; DG, diacylglycerols; HexCer, hexosylceramides; LPC-O, ether-linked lysophosphatidylcholines; LPE, lysophosphatidylethanolamines; PC-O, ether-linked phosphatidylcholines; PE-O, ether-linked phosphatidylethanolamines; PI, phosphatidylinositols; PS, phosphatidylserines; SM, sphingomyelins; TG, triacylglycerols; Cer, ceramides; LPC, lysophosphatidylcholines; PC, phosphatidylcholines; PE, phosphatidylethanolamines. Annotations indicate fatty-acyl chain length and number of carbon-carbon double bonds. The pairwise comparison matrix highlights significant differences between groups. **b** Principal component analysis of lipidomic profiles from WT and ATG KO cells. **c** Representative confocal images (three independent experiments) of BODIPY 493-labeled lipid droplets (LDs; gray-LUT) and nuclei (blue). Insets show boxed areas. Scale bar = 10 µm, insets, 5 µm. **d** Average LD number and area per cell were quantified from images. Quantification was performed using 3 biological replicates. **e** Representative flow cytometry histograms of neutral lipid content as measured by BODIPY 493 staining. Quantification of mean fluorescence intensity of BODIPY 493 in 10,000 cells per independent biological replicate (*n* = 3) and expressed relative to WT. **f** Cellular TG content expressed relative to WT, shown as mean ± SEM, from 3 biological replicates. **g** Representative confocal images of ceramide (magenta-LUT), TGN46 (green-LUT), and nuclei (blue). Insets show boxed areas. Quantification of ceramide intensity in the TGN was performed from 80 cells acquired in three biological replicates. Scale bars = 20 µm; insets, 5 µm. **h**,** i** Representative flow cytometry histograms and quantification of BODIPY-C5-Sphingomyelin and BODIPY-C5-Ceramide fluorescence in 10,000 cells per replicate (*n* = 3 independent experiments). Data in (**d–i**) are mean ± SEM. Statistical significance was calculated using one-way ANOVA with Tukey’s multiple comparisons test, and only significant results are shown: * *P* < 0.05, ** *P* < 0.01, *** *P* < 0.001, and **** *P* < 0.0001.

Since ATG9A and ATG2 proteins are directly involved in autophagosome formation and expansion via lipid mobilization between membranes^6^, may also function in other lipid-homeostatic membrane pathways, and we observed increased ferroptosis markers both inside the cells and the secreted EVs (Fig. 4c–e), we wondered if lipid flux perturbation in the autophagy-deficient cells contributed to the disruption in EV biogenesis. To tackle this question, we first performed untargeted lipidomic profiling using mass spectrometry. Whole-cell lysates and EVs were prepared and isolated from WT and ATG KO cells, respectively. Lipidomic analysis of whole-cell lysate revealed striking alterations in lipid composition in both ATG9A and ATG2AB DKO cells when compared to WT cells (Fig. 5a, Supplementary Data 3). Except for minor differences driven by upregulation of polyunsaturated phosphatidylcholine (PC) species and their ether forms (PC-Os), the lipidomic profile of ATG7 KO cells was very similar to WT (Fig. 5a, b). Significant accumulation of ceramides, sphingomyelins, diacylglycerides (DG), triacylglycerides (TG), and PC-Os and overall depletion of PC, phosphatidylethanolamine (PE), and phosphatidylserine (PS) glycerophospholipids was observed in ATG9A and ATG2AB DKO cells when compared with WT (Fig. 5a). In addition to these global changes, we observed alterations in lipid acyl chain properties, including chain length and number of carbon-carbon double bonds with a remarkable downregulation of polyunsaturated PCs and a concomitant increase in the presence of these acyl chains in DG/TG species in ATG2AB DKO cells (Fig. 5a). While PC is ubiquitously present in all cellular membranes, SM, PE, and PS (for synthesizing PE) are the major lipid species involved in autophagosome formation^59,60^. The notable difference in DG and TG accumulation aligns with previous reports of accumulation of larger lipid droplets in ATG9A and ATG2AB DKO cells^34^, a phenotype that we could recapitulate in our cells as well: staining with BODIPY 493, which has affinity for neutral lipids, followed by confocal microscopy (Fig. 5c, d) and flow cytometry analysis of total fluorescence (Fig. 5e) revealed an increase in lipid droplets only in ATG9A and ATG2AB DKO cells. Relevant to sEV biogenesis, we also detected significant changes in ceramide levels among these cells (Fig. 5a). Staining with anti-ceramide antibody showed a higher punctate accumulation of ceramides in the *trans*-Golgi network in all the ATG-deficient cells with a significant increase in both ATG9A and ATG2AB DKO cells (Fig. 5g)^61^. Incubation of WT and ATG KO cells with BODIPY-conjugated ceramide and sphingomyelin (a precursor of ceramides) showed higher intracellular signal in ATG2AB DKO cells, suggesting impaired turnover and trafficking into membranes and organelles in these cells (Fig. 5h, i). Ceramide and SM have been implicated in EV biogenesis^62–64^. Given that the accumulation of ceramides increases EV secretion^65^, we utilized GW4869, an nSMase2 inhibitor, to prevent ceramide production^66^. We observed a complete recovery in EV secretion in ATG9A and ATG2AB DKO cells to similar levels in the WT and ATG7 KO cells by analyzing both sEV concentration (Supplementary Fig. 7a) and CD63 content per sEV (Supplementary Fig. 7b, c). These results support the observation that in the ATG9A and ATG2AB KO cells, devoid of amphisomes, the increase in sEVs is directly modulated by the excess in Cer/SM.

Similar lipidomics analysis of EVs isolated from the WT and ATG KO cells was performed, despite the limited starting material (Supplementary Fig. 7d, e, Supplementary Data 4). While the replicates were more dispersed than those obtained from whole cells (Supplementary Fig. 7d, e), analysis of the clustering of the different lipid species showed significantly differentially detected lipids in the EVs from ATG7 KO cells compared to the WT. In almost all the lipid categories, ATG7 KO EVs showed enrichment of phospholipids and cholesterol relative to those isolated from WT cells (Supplementary Fig. 7d). Remarkably, the lack of statistically significant differences in the lipidome of EVs between the ATG9A KO, ATG2AB DKO, and WT was in striking contrast to the changes observed inside the cells.

**Fig. 6:**
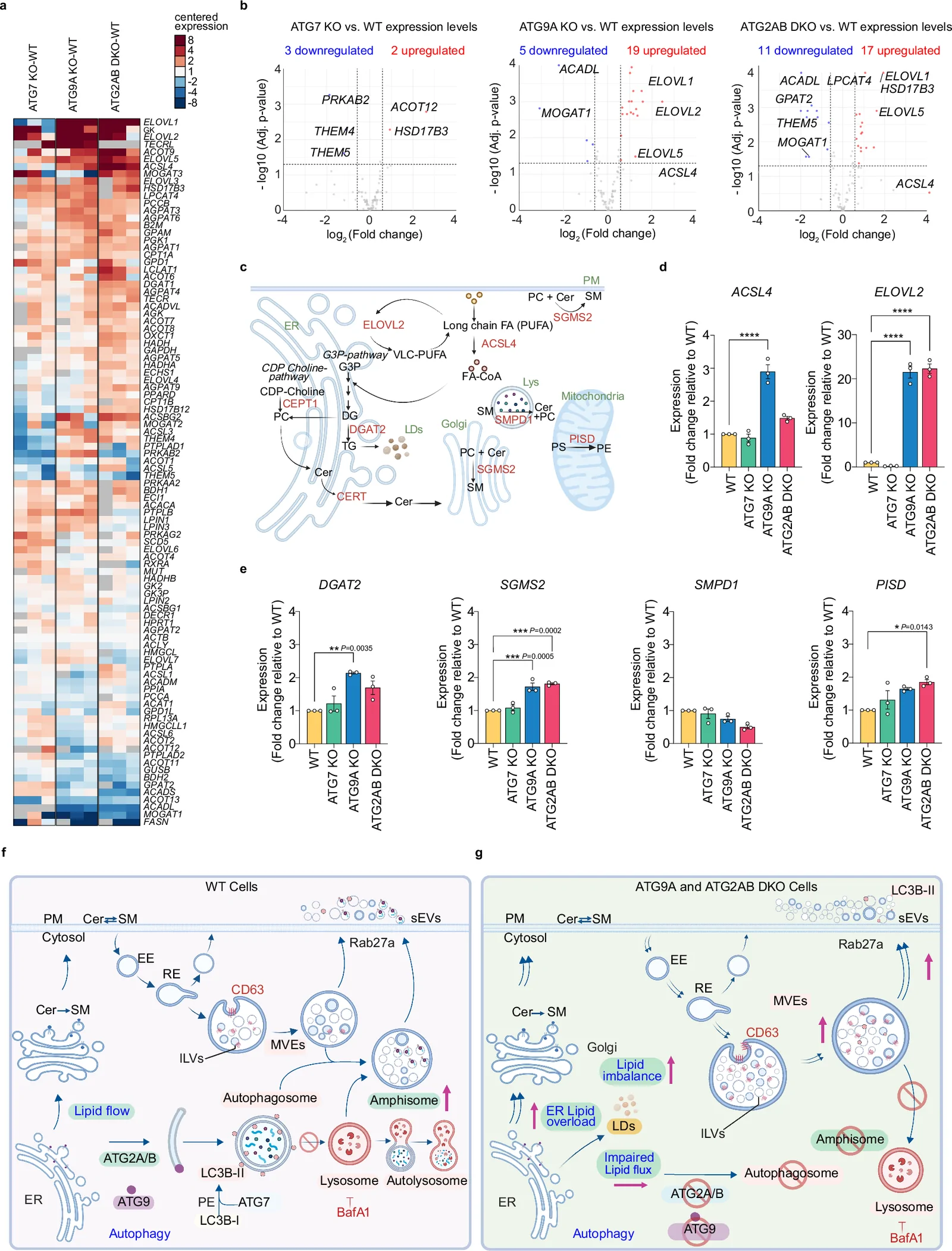
Autophagy-deficient cells show alterations in gene expression for key lipid processing pathways. **a** Heat map showing the differential expression of fatty acid metabolic genes in ATG KO cells compared to WT HeLa cells (*n* = 3 independent biological replicates). Log2-scaled abundances were median-centered within each sample by subtracting the sample-specific median log2 abundance, thereby aligning sample medians to zero while preserving within-sample rank structure. Data shown as log_2_ fold changes represented by its normalized centered expression intensity on a −8 to +8 scale from blue (decreased level) to red (increased level). **b** Volcano plot depicting differentially expressed genes described in (**a**), showing the significant upregulated and downregulated genes in ATG KO cells compared to WT. y-axis depicts -log_10_(adjusted *p*-value), while x-axis depicts log_2_(fold change). Differential expression was assessed using two-sided empirical Bayes moderated t-tests implemented in limma (version 3.56.2). Multiple-testing correction was performed using the Benjamini-Hochberg method, with adjusted *P*-values reported as false discovery rates. **c** Schematic representation of the main biochemical reactions with key genes involved in fatty acid processing, ceramide and sphingomyelin regulation, and phospholipid balance in the cell. Lys: lysosome; PM: plasma membrane; ER: endoplasmic reticulum. Created in BioRender. Singh, A. (2026) https://BioRender.com/q1k176n and modified in Adobe Illustrator. **d**,** e** Expression level of selected fatty acid metabolic genes by qRT-PCR in WT and ATG KO cells. Data are shown as mean ± SEM (*n* = 3 independent biological replicates). Statistical significance was calculated using one-way ANOVA with Tukey’s multiple comparisons test, and only significant results are shown: * *P* < 0.05, ** *P* < 0.01, *** *P* < 0.001, **** *P* < 0.0001**. f**,** g** A model summarizing a putative mechanism of sEV biogenesis and the role of amphisome-mediated secretion in WT cells (**f**). The scheme also highlights the increase in amphisome formation and secretion in the absence of functional lysosomes. **g**, The increase in sEV biogenesis in the absence of lipid -handling proteins ATG9A and ATG2 due to impaired flux of lipids from the ER (LD accumulation), depletion of amphisomes, and an increase in CD63-positive ILVs in MVEs. The schematics were drawn using BioRender Created in BioRender. Singh, A. (2026) https://BioRender.com/7s6se94 and modified in Adobe Illustrator.

Given the changes in cellular lipidome in the ATG KO cells and the surprising shifts in lipid composition, we speculated that these changes must affect the regulation of the critical enzymes involved in lipid metabolism, whether as a direct consequence of autophagy inhibition or a compensatory reaction. For that, we evaluated the mRNA expression of several enzymes involved in fatty acid biosynthesis, elongation, TG synthesis, phospholipid synthesis, and ceramide and sphingomyelin metabolism pathways, using an RT-qPCR targeted screen^67^ (Fig. 6a, Supplementary Data 5). Significantly regulated genes were observed in the three KO cells, with ATG9A KO and ATG2AB DKO showing the largest changes and overlap of genes (Fig. 6b). Upregulation of ELOVL genes and ACSL4 suggested increased elongation of fatty acids (FA) (mainly long-chain polyunsaturated fatty acids (PUFAs)^68,69^ and activation to FA-Acyl-CoA (Fig. 6c), respectively, in these KO cells, which prompted us to validate those genes using different primers. Consistent with this, ELOVL2 showed a dramatic 20-fold increase in ATG9A KO and ATG2AB DKO cells relative to WT (Fig. 6d). While ACSL4 was slightly increased in ATG2AB DKO cells, it was significantly upregulated in ATG9A KO cells (Fig. 6d), confirming the screen results. Downstream of FA activation, DGAT2 is a critical enzyme to convert DGs into TGs that will be stored in LDs^70,71^. Given the accumulation of these organelles in the ATG9A KO and ATG2AB DKO cells (Fig. 5c, d), we also evaluated the levels of expression of this enzyme that was not included in the original targeted screen. RT-qPCR experiments showed significant upregulation of DGAT2 in both KO cells compared to WT, in line with the LD phenotype (Fig. 6e).

As the biogenesis of EVs is also regulated by SM and Cer, and we observed changes in these lipid classes in the lipidomics experiments (Fig. 5a), we next interrogated the key enzymes in the biosynthesis of these lipids: SGMS2 and SMPD1 (Fig. 6e, Supplementary Information Table 3)^72^. SGMS2 is a Golgi and plasma membrane-enriched enzyme that converts PC and Cer into SM and DGs^73^, and SMPD1 is localized in endolysosomes and is responsible for performing the opposite reaction^74^. While we observed upregulation of SGMS2 in ATG9A KO and ATG2AB DKO (Fig. 6e), SMPD1 was significantly downregulated in ATG2AB DKO (Fig. 6e), complementing the results we observed in the lipidomics of these cells, where significantly increased levels of Cer and reduced levels of PUFA-containing SM species (Fig. 5a) were detected. The lack of differences in lipid metabolism genes in ATG7 KO cells points to a dispensable role of downstream autophagy machinery in the lipidome changes, as previously suggested by our lipidomics data (Fig. 5a).

Altogether, this series of experiments indicates that the deletion of key lipid transfer proteins ATG9A and ATG2 can significantly alter cellular lipid pools, leading to dysfunctional lipid flux, accumulation of lipid droplets and sphingolipid imbalance in cells, which in turn could affect membrane trafficking pathways and sEV biogenesis (Fig. 6f, g).

## Discussion

Over the past decade, multiple lines of work have established that autophagy is deeply interwoven with the endo-exocytic system rather than operating as an isolated pathway dependent on lysosomal degradation. Early studies demonstrate that autophagosomes intersect with endosomes to form amphisomes and that components of the MVE pathway influence autophagic flux, placing cargo sorting and vesicle dynamics upstream of degradative fusion (reviewed in^30,75^). More recent datasets extend this view by connecting LC3-conjugation machinery and endosome-derived vesicles to selective cargo loading into EVs^19,29^. Parallel advances have reframed “the lysosome” as a heterogeneous family of organelles that differ in position, acidity, enzyme content, and signaling competence^76–78^. Such heterogeneity likely dictates which autophagosomes (or amphisomes) they fuse with and when. Mechanistically, this crosstalk is supported by shared effectors: ESCRT modules function in autophagosome closure, ILV biogenesis, and amphisome maturation, while class III PI 3-kinase complexes generate the PI3P landscapes that license both autophagosome nucleation and endosomal maturation^79–81^, reinforcing the need for a model of a shared membrane economy that allocates material between degradation and secretion rather than two insulated pathways.

Our work focused on the study of four particular ATG KO cell lines: ATG7, ATG9A, ATG2A/B, and FIP200. They block autophagy progression or efficacy at different stages. ATG7 deletion disrupts ATG8 lipidation and selective autophagy cargo handling, whereas FIP200 deletion blocks the upstream autophagy-initiation complex. In contrast, ATG9A and ATG2A/B act at the lipid-transfer and membrane-expansion steps of phagophore growth and also participate in other membrane-homeostatic processes. This comparison revealed that loss of ATG9A or ATG2A/B produced the strongest sEV phenotype: increased secretion of smaller CD63-enriched vesicles, accumulation of ILVs inside MVEs, and elevated intracellular CD63 (Fig. 1b–i, Fig. 2a–h). FIP200 KO cells shared the autophagy-cargo defect, including impaired LC3B export into sEVs, but did not show increased sEV particle output or lipid droplet accumulation (Supplementary Fig. 4a–d). These data argue that impaired autophagosome/amphisome formation is sufficient to alter EV cargo routing, but that the hypersecretory EV phenotype requires disruption of the ATG9A/ATG2A/B lipid-handling axis. Given a recent study demonstrating CD63 as a lipid-sorting protein to EVs^82^, together with the established notion that lipid perturbations affect exosome biogenesis^83^ and the autophagy-dependent and -independent roles of these ATG proteins in lipid mobilization^34,35,84–88^, we speculated whether lipid reorganization could explain the selective enhancement of EV secretion in ATG9A KO and ATG2AB DKO cells.

We showed a significant imbalance in whole-cell lipidome in ATG9A and ATG2AB KO cells (Fig. 5a, b), with massive accumulation of SM/Cer and DG/TG of PUFAs sequestered and accumulated in LDs, among other phospholipid species, indicating an almost complete rewiring of the lipidome when these proteins are absent. De novo phospholipid synthesis and targeted lipid transfer are increasingly appreciated as core drivers of phagophore growth^89–92^, and lipid profiling indicates autophagosome membranes are enriched in PC and highly unsaturated species that favor curvature and rapid remodeling^91,93^. We speculated that the absence of phospholipid mobilization during autophagosome generation could produce an ER-controlled reorganization that led not only to the accumulation of LDs but also to an imbalance in SM/Cer. Sphingolipid metabolism has been linked to both autophagy^94,95^ and EV biogenesis control^62–64^. SM accumulation in SMPD1-deficient cells disrupts autophagosome closure by perturbing ATG9A cycling, suggesting a direct sensitivity of ATG9A-dependent membrane dynamics to SM/Cer balance^96^. Coupled with our whole-cell lipidomics and transcriptional remodeling of enzymes such as SGMS2, SMPD1, ELOVL2, ACSL4, and DGAT2 (Fig. 6d, e), a cohesive picture emerges: ATG9A/ATG2 normally maintain a lipid environment permissive for phagophore growth and amphisome fusion; when this is lost, excess or mischanneled lipids are available to the endosomal system, accelerating ILV budding and MVE fusion frequency, thereby increasing exosome output while starving the amphisome route of the right lipid cues. The particular details of how the flux of each lipid species is regulated, however, remain to be tested.

Our findings support a model in which the autophagy lipid-supply factors ATG9A and ATG2A/B are required for membrane fate regulation between degradative (lysosome) and secretory (amphisome/exosome) pathways. When these factors are disabled, endosomal systems skew toward increased ILV production and RAB27A-dependent fusion at the plasma membrane, yielding a surge of CD63^+^ sEVs, while LC3-positive autophagic cargoes fail to access EVs under lysosomal inhibition. Because super-resolution microscopy showed a lack of LC3-CD63 content mixing (Fig. 2i), and amphisomes, although acidic, lack key lysosomal hydrolases such as acid phosphatase and cathepsin B^97^, the inner autophagosomal membrane is unlikely to be enzymatically degraded, so the autophagosome and ILV contents would remain segregated. In addition, unbiased proteomics results demonstrated selective depletion of canonical autophagy receptors from sEV fractions (Supplementary Fig. 5a–e), indicating that amphisome formation (not merely LC3 lipidation at non-autophagosomal sites) is a major gating step for SALI-type cargo export. This mechanistic decoupling clarifies a long-standing ambiguity in the field: exosome upregulation does not imply secretory autophagy. Rather, it can partially emerge from a lipid-state shift that simultaneously impairs amphisome competency and stimulates MVE biogenesis and exocytosis.

These insights can also be interpreted in the context of the different models of autophagosome biogenesis. Yeast-based work emphasized PC/PI-rich Atg8 membranes with striking unsaturation, a composition well suited for rapid expansion and curvature, and proposed that localized de novo synthesis is key to net lipid transfer from the ER to the phagophore^10,91,93^. More recent mammalian evidence suggests ATG9A compartments (that give rise to autophagosomes) possess a distinct lipid makeup (enriched in PE and cholesterol, relatively low in PI)^98^, and posits that PI and/or PI3P may be dynamically supplied (potentially through ATG2A-mediated transfer from the ER and recruitment of PI3KC3-C1 via RAB1) before or during early phagophore nucleation^98^. While we cannot adjudicate among these models here, our data are fully consistent with the conclusion that the site and timing of lipid delivery are decisive variables: mis-timed or mis-localized PI/PI3P production, altered PE/PC/SM balance, or excess neutral lipid buffering could each push membranes away from amphisome formation and toward ILV-specific production. Notably, autophagosomal membranes are protein-poor, implying a bioenergetic and logistical advantage for building them from freshly synthesized lipids rather than stripping proteins from existing membranes^89,99,100^; when autophagosome formation is blocked, this flux could back up at the ER and be rerouted into endosomal pathways, amplifying MVE activity and exosome secretion. These concepts also align with classical observations that enhancing degradative autophagy often suppresses EV release, underscoring a see-saw between the two fates^25,50^.

Our data sharpen two conceptual advances. First, they place amphisomes at the heart of SALI cargo transfer into EVs under lysosomal inhibition, resolving why LC3 and cargo receptors are abundant in sEVs from wild-type cells but depleted when ATG9A/ATG2 are absent, despite increased EV numbers^19^. Second, they identify lipid-supply autophagy proteins as players in MVE dynamics, supporting a model in which the *quality* of lipid input (unsaturation, headgroup identity, ceramide/SM ratios) determines whether LC3^+^ membranes fuse with MVEs or not. This framework generates testable predictions at single-organelle resolution such as elevating local SM or depleting PE at endosomes should decrease amphisome formation while increasing ILVs and buffering DG/TG via DGAT2 inhibition should shift the balance back toward degradative routing by limiting neutral-lipid sinks that otherwise promote ILV budding. These hypotheses will need to be tested in future research.

The physiological impact of the sEV surge in the ATG KO cells is equally important. EVs may offload lipotoxic species or, conversely, propagate maladaptive stress signals^101–103^. Our initial observations prompt targeted tests in recipient cells such as neurons, fibroblasts, or macrophages to assess lipid droplet accumulation, inflammatory responses, oxidative stress tolerance, and ferroptosis sensitivity following uptake of EVs from ATG9A/ATG2-deficient versus WT cells, particularly in light of our findings showing a significant enrichment of ferritinophagy-related proteins in sEVs under SALI-induced stress (Fig. 4c). Orthogonal in vivo and organoid models will be essential to evaluate whether this secretory skew is beneficial (lipid detoxification, paracrine support) or deleterious (inflammatory activation, proteostasis disruption) in tissue contexts relevant to neurodegeneration, metabolic disease, and cancer. Although secretory autophagy is often viewed as a compensatory route engaged when lysosomal function is compromised or cellular stress exceeds degradative capacity^104,105^, the resulting diversion of cargo to the extracellular space not only can be leveraged as a readout of autophagy-lysosome system status but, in multicellular contexts, likely represents an evolutionarily co-opted mode of intercellular communication rather than mere cytosolic waste disposal^106–108^. In turn, this selective export endows EVs with high translational value as accessible biomarkers for disease onset, progression, and therapeutic response in disorders such as cancer and neurodegeneration, underscoring the need to resolve how EVs are generated and loaded with cargo, questions that remain unanswered.

Limitations of our current study should be acknowledged. EV preparations remain heterogeneous despite SEC and orthogonal characterization, leaving residual uncertainty about the exact vesicle subclasses carrying (or lacking) LC3 and cargo receptors. The detection of ectosomal markers such as CD98 and CD147 indicates that plasma membrane-derived ectosomes may contribute to the isolated sEV-enriched fractions, particularly under lysosomal inhibition. Future iterations will combine buoyant-density gradients, tetraspanin-specific immunocapture, and single-vesicle analyses to quantify co-positivity rates and reduce route conflation in a manner consistent with MISEV best practices^109^. Our ‘omics signatures, while striking, are correlative rather than causal. In addition, because most phenotypes were defined in chronic KO cells, long-term adaptation to loss of ATG9A, ATG2A/B, ATG7, or FIP200 cannot be excluded. Therefore, acute perturbation systems, including AID (auxin-inducible degron)- or dTAG (degradation tag)-based endogenous degradation, acute lipid editing, and structure-function rescues, will be needed to dissect sufficiency, necessity, and temporal order. Finally, our initial work centers on transformed cell lines. Extending to primary cells and in vivo systems will test generality and reveal organismal consequences.

In summary, we define a lipid-controlled checkpoint, governed by the autophagy lipid-supply machinery, that partitions membranes between degradative autophagy through amphisomes and secretion through MVEs that release EVs. Disrupting this lipid supply increases vesicle output yet prevents the lysosome-inhibition–driven export of autophagy components and cargo receptors, establishing amphisome formation as a major gate for secretory autophagy. Systematically modulating lipid-metabolic enzymes and endosomal trafficking adapters should enable precise control of degradative versus secretory routing in physiology and disease.

## Methods

### Cell lines

Human HeLa (ATCC, CCL-2) and HEK293T cells (ATCC, CRL-3216) were grown in complete medium (CM) consisting of Dulbecco’s-modified Eagle’s medium (DMEM, ATCC, 30-2002) supplemented with 10% fetal bovine serum (FBS, Gemini Bio, 100-106-500), 100 IU/mL penicillin, and 100 μg/mL streptomycin (ThermoFisher, 15140122) at 37 °C and 5% CO_2_. EV-depleted FBS-containing medium was prepared by ultracentrifugation of 20% FBS in DMEM at 120,000 x *g* for 16 h^110^. Where indicated, cells were also treated with 100 nM Bafilomycin A1 (BafA1) (Millipore Sigma, 196000) or 5 µM nSMase inhibitor (GW4869) (Millipore Sigma, D1692) for 16 h (EVs isolation) or 3 h (autophagy flux assays).

### Antibodies and reagents

We used the following antibodies for immunofluorescence staining (IF), flow cytometry (FACS), and immunoblotting (IB): horseradish peroxidase (HRP)-conjugated anti-GFP (IB 1:5,000; Miltenyi Biotech, 130091833), anti-ATG7 (IB 1:1,000; Cell Signaling Technology, 8558), anti-ATG9A (IB 1:1,000; IF 1:500; Abcam, ab108338), anti-ATG2A (IB 1:500; Proteintech, 23226-1-AP), anti-ATG2B (IB 1:500; Proteintech, 25155-1-AP), anti-GAPDH-HRP (IB 1:1,000; Santa Cruz Biotechnology, sc-47724), anti-LC3B (IB 1:1,000; IF 1:100; Cell Signaling Technology, 3868), anti-CD63 (IB 1:1,000; IF 1:500; BD Bioscience, 556019), anti-Calnexin (IB 1:1,000; Cell Signaling Technology, 2679), CD63-Alexa Fluor-647 (FACS 1:100; BD Biosciences, 561983), Alexa Fluor-647-IgG1 κ Isotype Control (FACS 1:100; BD Biosciences, 557714), anti-PLIN3/TIP47 (IF 1:500; Proteintech, 10694-1-AP), anti-RAB27A (IB 1:1,000; Cell Signaling Technology, 69295), TGN46 (IF 1:500; Bio-Rad, AHP500G), anti-Ceramide (IF 1:500; Sigma, C8104), TAX1BP1 (IB 1:1,000; IF 1:500; Bethyl, A303-791AT), SQSTM1 (IB 1:1,000; IF 1:500; Progen, GP62-C), FTH1 (IB 1:1,000; IF 1:500; Cell Signaling Technology, 4393 T), anti-FIP200 (IB 1:1,000; Cell Signaling Technology, 12436). We also used the following secondary antibodies: Star Red anti-rabbit (STED 1:500; Abberior, STRED-1002), Star Orange anti-mouse (STED 1:500; Abberior, STRED-1001), Alexa Fluor 488-conjugated donkey anti-mouse IgG (IF 1:500; Invitrogen, A32766), Alexa Fluor 555-conjugated donkey anti-mouse IgG (IF 1:500; Invitrogen, A32773), Alexa Fluor 555-conjugated donkey anti-rabbit IgG (IF 1:500; Invitrogen, A32794), Alexa Fluor 488-conjugated donkey anti-rabbit IgG (IF 1:500; Invitrogen, A32790), Alexa Fluor 647-conjugated donkey anti-guinea pig IgG (IF 1:500; Invitrogen, A21450), HRP-conjugated anti-guinea pig IgG (IB 1:5,000; Jackson Immuno Research, 706-035-148), HRP-conjugated anti-rabbit IgG (IB 1:10,000; Amersham, NA934V), and HRP-conjugated anti-mouse IgG (IB 1:10,000; Jackson Immuno Research, 115-035-174). Several lipid probes were used in this study: BODIPY 493/503 (Thermo Fisher Scientific, D3922), BODIPY 558/568 Red C12 (Thermo Fisher Scientific, D3835), BODIPY FL C_5_-Sphingomyelin (Invitrogen, D3522), and BODIPY FL C_5_-Ceramide (Invitrogen, D3521).

### Generation of HeLa KO cells

ATG7 KO, ATG9A KO, ATG2AB DKO and FIP200 KO HeLa cells were generated using the CRISPR-Cas9 system. For ATG9A and FIP200, sgRNA sequences complementary to the first exon of human ATG9A and second exon of FIP200 were designed using Benchling 2021 (https://www.benchling.com) and were cloned into pSpCas9(BB)-2A-GFP (pX458) (gift of Feng Zhang, Addgene, 48138). For ATG7, ATG2A and ATG2B, gRNA-Cas9 plasmids were used as previously described^34^ (Supplementary information Table 1). These plasmids were transfected into HeLa cells and, 24 h later, GFP-positive cells were single-sorted in a 96-well plate on a BD FACS Aria III flow cytometer. After several days, the single clonal population of cells was analyzed by immunoblotting to confirm KO of the genes. ATG2A KO cells were subsequently transfected with the pX458 plasmid containing gRNA targeting ATG2B to generate ATG2AB DKO cells (Supplementary information Table 1).

### Generation of stably-transfected cell lines using lentivirus

Complementary DNA (cDNA) encoding human ATG2A with an N-term GFP tag was subcloned in the pMRXIP vector (a gift from Noboru Mizushima, Addgene, 114462). Human ATG9A-GFP (as described previously^34,111^) and human ATG7 (a gift from Eiki Kominami & Isei Tanida, Addgene, 87867) with an N-terminal GFP tag were subcloned into pLKO.1-puro lentiviral expression vector to generate ATG9A-GFP and GFP-ATG7 plasmids, respectively. pEGFP-C1-SopF (a gift from Leigh Knodler, Addgene 137734) was subcloned into pLKO.1-puro lentiviral expression vector. All lentiviruses were packaged in HEK293T cells according to published protocols^112^. Briefly, cells were transiently transfected with psPAX2 (a gift from Didier Trono - Addgene, 12260), pMD2.G (a gift from Didier Trono - Addgene, 12259), and the corresponding expression plasmids using Lipofectamine 2000 (ThermoFisher, 11668019). Supernatants were collected 48 h post-transfection, aliquoted, and stored at −80 °C. Titers were determined using digital droplet PCR (Bio-Rad, QX200) to measure the number of lentiviral particles integrated into transduced HEK293T cells using FAM-labeled GAG probe to detect the lentiviral gag gene expression (Supplementary information Table 3). To generate stable ATG rescued and GFP-SopF expressing cell lines, HeLa cells were infected with 1:10 MOI of the respective GFP-tagged lentiviruses. Cells were cultured under puromycin (ThermoFisher, A1113803) selection at a 2 µg/mL concentration. After 48 h, stable cells were expanded, sorted by FACS (single clones for ATG rescue experiments; in bulk for GFP-SopF transduced lines), and propagated until they were checked by immunoblotting against GFP to confirm the overexpression. pGIPZ shRNA vectors (Horizon Discovery) were used to deliver shControl and shRAB27A (Supplementary information Table 2) after generation of lentiviral particles as above. HeLa shControl and shRAB27A stable lines were generated by infection with 1:10 MOI of lentiviruses along with 1 µg/mL of polybrene (Millipore Sigma, H9268) for 24 h. After 48 h, the medium was replaced with 2 µg/mL puromycin containing medium. Stably selected cells were confirmed immunoblotting for RAB27A.

### sEVs isolation

Equal numbers of WT and ATG KO cells were seeded at 28,000 cells per cm^2^ in a 150-mm cell culture dish in CM. At 80% confluency, cells were cultured in EV-depleted FBS-containing medium. When specified, 100 nM bafilomycin A1 (BafA1) or 5 μM GW4869 was used for 16 h before collecting the supernatant for sEV isolation as previously described^113^, with modifications. Briefly, the supernatant underwent sequential differential centrifugation at 400 × *g* for 10 min, followed by 800 × *g* for 10 min and then at 2000 × *g* for 10 min, followed by 12,000 × *g* for 45 min to remove cells, large debris, and apoptotic blebs, respectively. The resulting supernatant was filtered through a 0.2 μm Steriflip filter (Millipore Sigma, SCGP00525). Filtered conditioned media were then concentrated using 100 kDa Amicon Ultra 15 mL centrifugal filters (Millipore Sigma, UFC910024) to a final volume of 500 μL. Concentrates were loaded onto 70 nm qEV 500 size exclusion columns (Izon, SP1) for fractionation. Separate qEV columns were utilized for each cell and treatment. EV enriched fractions F7 to F10 (500 μL each) were pooled and used for downstream experiments^111^. Cells from which EV-enriched conditioned medium was derived were harvested by washing with 1x PBS, followed by detachment using 0.25% trypsin-EDTA (ThermoFisher, 25200056), and trypsin deactivation with CM. Cells were counted using an automated cell counter (Revvity, Cellometer, SD100). Total cell number was utilized to normalize sEV secreted per cell by dividing total EV particle concentration obtained from Nanoparticle Tracking Analysis (NTA) (see below). Equal numbers of particles were used for comparison of EV content by immunoblotting. For lipidomic and proteomic studies (see below), sEVs were isolated using the EXODUS method^51^. Briefly, 0.2 μm filtered conditioned media were directly loaded into medium exosome isolation devices (EIDs) (Exodus Bio, MA03). After isolation was done, sEVs were recovered from EIDs by pipetting up and down ( ~ 20 times) 1 mL of PBS per device. To estimate total protein content, a fraction of sEVs was ultracentrifuged (Beckman Coulter, Optima TLX) at 65,000 × *g* in TLA-55 fixed-angle rotor (Beckman Coulter, 366725). The pellet was lysed in RIPA lysis buffer containing 1 mM phenylmethylsulphonyl fluoride (PMSF, Thermo Scientific, 36978) and protease inhibitor cocktail (PIC, Thermo Scientific, 78430) on ice for 30 min, with vortexing every 10 min. Protein estimation was carried out using Pierce BCA Protein Assay Kit (Thermo Scientific, 23225). sEV volume corresponding to 30 μg protein was pelleted and used for mass spectrometric analyses.

### NTA

To measure particle concentration and size distribution of EVs, NTA was performed on a Malvern Nanosight NS300 unit equipped with a 488 nm laser and a high-sensitivity sCMOS camera (NanoSight, Malvern, Worcestershire, UK). Freshly isolated sEVs by SEC were diluted in 0.1 µm-filtered PBS to achieve a particle concentration of 1 × 10^6^–1 × 10^9^ particles/ mL and were analyzed using the NanoSight 3.3 Dev Build 3.3.301 software. The sample infusion motorized syringe pump was set at a speed of 30/AU (AU, arbitrary unit) to inject the sample suspension into the flow cell. The camera levels were set at a range of 13–16 for optimal visualization of particles. 3 to 5 videos of 60 s each with 1 s delay with viscosity of water at room temperature were captured in script control mode. The detection threshold was set for high sensitivity and minimum background noise, with levels set at 8/AU. For any given set of experiments, all the parameters were kept constant.

### CryoEM grid preparation and screening of EVs

Approximately 3 µL of isolated sEVs were applied to freshly glow-discharged Lacey Carbon 300-mesh grids (EMSS, CFT313-50). The sample was incubated on the grid for 1 min in a Leica EM GP2 environmental chamber (Leica Microsystems) set to 95% relative humidity and 15 °C for 90 s. After incubation, excess sample was removed by blotting with a double layer of Whatman Grade 40 filter paper for 3 s. The grid was then rapidly plunge-frozen into liquid ethane at –182 °C. The cryo-EM grids were screened at 22000x magnification on 200 kV Talos Arctica microscope (Thermo Fisher Scientific) equipped with Gatan K2 summit detector (Gatan). 200 micrographs for each sample were collected using the SmartScope program^114^ for further analysis. The diameter of randomly selected individual sEVs was calculated manually by drawing a straight line through the EV using the line profile tool in Fiji software (https://fiji.sc).

### Transmission electron microscopy (TEM)

Cells were trypsinized and initially fixed in Modified Karnovsky’s fixative (2.5% glutaraldehyde/2% paraformaldehyde in 0.1 M sodium cacodylate buffer, pH 7.4) at room temperature for 2 h. Samples were rinsed with 0.1 M sodium cacodylate buffer, post-fixed in 1% osmium tetroxide in phosphate buffer, rinsed in distilled water, dehydrated through a graded ethanol series (70%, 75%, 95%, 100%), and transitioned to acetone using a Leica EM TP processor. The samples were then infiltrated with increasing concentrations of Polybed 812 resin (1:3 resin/acetone, 1:2, 1:1 to absolute resin), embedded in absolute resin, and placed in an oven at 60 °C for 48 h to polymerize. Once polymerized, blocks were trimmed and semithin sections approximately 0.5 µm thick were cut, mounted on glass slides, and stained with 1% toluidine blue O in 1% sodium borate. The sections were examined by light microscopy to identify the areas of interest. Each block was trimmed to the area of interest, and thin sections approximately 70 nm thick were placed on a 200-mesh copper grid and stained with 3% aqueous uranyl acetate and Reynolds lead citrate. Digital images were captured at an AMT BioSprint16AV 16-megapixel bottom-mount camera attached to a JEOL JEM-1400+ transmission electron microscope operating at an accelerating voltage of 80 kV. Median filter was applied on raw electron microscopy images in Fiji/ImageJ. The diameter of ILVs within randomly selected MVEs was calculated manually by drawing a straight line through measurable distance of the ILV using the line profile tool in Fiji software (https://fiji.sc). MVEs were identified by the morphology and pattern of ILV distribution.

### Autophagy flux assay

To measure autophagic flux, WT and ATG KO cells (500,000 cells per well) were seeded in 6-well plates and were treated with 100 nM BafA1 in either control medium (CM) or starvation medium (EBSS, Thermo Fisher Scientific, 24010043) for 3 h before collecting cell lysates for immunoblotting of LC3-I/II.

### Immunoblotting

Cells were lysed in RIPA lysis buffer containing 1 mM PMSF and protease inhibitor cocktail on ice for 30 min, with vortexing for a few seconds every 10 min. Lysate was clarified by centrifuging (17,000 × *g* for 15 min at 4 °C). The clear supernatant was collected and protein concentrations were estimated using Pierce BCA Protein Assay Kit (Thermo Scientific, 23225). Equal amounts of lysate in either reducing or non-reducing (for detection of CD63) SDS-sample loading buffer were incubated at either 37 °C (for probing ATG9A) or 95 °C for 5 min. The samples were separated on 4–20% sodium dodecyl sulfate–polyacrylamide gels (Biorad, 5671093) and transferred to LF-PVDF membranes (Bio-Rad, 1704275). Membranes were blocked (5% skimmed milk in TBS-T: Tris buffered saline with 0.1%Tween 20) for 1 h at room temperature and incubated at 4 °C overnight with indicated primary antibody in blocking buffer. After three washes in TBS-T, membranes were incubated with HRP-conjugated secondary antibodies in 2% blocking buffer for 1 h at room temperature. After three washes, detection was performed using Immobilon Forte Western HRP substrate (Merck, WBLUF0500), and signals were visualized with the ChemiDoc Touch Imaging System (Bio-Rad, USA). Band intensities were quantified using ImageJ software.

### Immunofluorescence

Cells were plated on round 12-mm glass coverslips and when ready to process, washed three times with PBS, followed by fixation with 4% paraformaldehyde (Electron Microscopy Sciences, 15714) in PBS for 15 min at room temperature. Excess fixative was rinsed three times with PBS and cells were permeabilized with 0.1% Triton X-100 in PBS for 5 min at room temperature. After three washes with PBS, cells were blocked in 0.5% BSA (Goldbio, A-421-100) in PBS. Cells were then incubated with primary antibody at indicated dilutions (antibodies and reagent section) in blocking buffer for 30 min at 37 °C, followed by washing with PBS three times and incubation with secondary antibody in blocking buffer for 30 min at 37 °C. Cells were washed three times with PBS and coverslips were mounted on glass slides using Fluor mount-G reagent with DAPI (Electron Microscopy Sciences, 17984-24). Cells were imaged in XYZ plane using 63×/1.40 NA Plan Apochromat Oil DIC M27 objective lens (Carl Zeiss) in confocal microscope (LSM780; Carl Zeiss). Laser power and gain settings were kept constant for images used for comparative analysis. Images were acquired and maximum-intensity projected using ZEN software (Carl Zeiss).

### Immunofluorescence imaging data analysis

All images were analyzed using Fiji/ImageJ. To quantify the signal intensity of CD63, cell boundaries were manually drawn using freehand selection tool. Area normalized signal intensity was calculated and used for analysis. For quantification of ceramide mean signal intensity within trans-Golgi network (TGN) areas, TGN channel was used to create a binary mask after thresholding. This mask was applied on ceramide channel and signal intensity for ceramide was evaluated within TGN areas per cell region of interest. Fiji/ImageJ was used to adjust brightness and contrast for presentation purposes. Average LD number and area per cell were quantified from images acquired using the Opera Phenix system and analyzed with Harmony evaluation software.

### STED microscopy

STED imaging was carried out using STEDYCON (Abberior Instruments) and Eclipse Ti2 inverted microscope (Nikon Inc.) or LED inverted epifluorescent microscope (Zeiss Colibri 7) equipped with either 100×, NA 1.45 Plan Apo objective as described previously^115^. Star Red-labeled samples were excited with 640 nm, and Star Orange with 561 nm, with an excitation laser of 10% power and depleted with the STED laser at 97.88% and 100% power, respectively. Images were captured using a fixed pinhole size of 32 μm with a 10 nm pixel size. Excitation Signals were detected within a 7-ns gate and Avalanche photodetectors (650–700 nm; 575–625 nm). Browser-based control software (Abberior Instruments) was used to generate STED images. Confocal images were acquired simultaneously with STED lasers blocked. These confocal images were used to define boundaries of the representative region of interest and drawn around visually identifiable CD63- and LC3B-positive compartments under low resolution conditions (not intended to define amphisome boundaries quantitatively), prior to acquisition of corresponding STED images. Image deconvolutions were performed by Huygens Software (Version 17.10.0p6 64b, SVI, Netherlands), using the Maximum Likelihood Estimation (CMLE) algorithm under default settings. ImageJ/ FIJI was used to add a scale bar on deconvoluted images.

### FACS analysis

Cells were harvested using PBS-EDTA, washed with PBS, and 5 × 10^5^ cells were suspended in FACS tubes. Cells at 4 °C were fixed with 2% paraformaldehyde for 15 min followed by permeablization with 0.2% saponin in FACS buffer (1% FBS in PBS) for 30 min. Cells were washed with FACS buffer (1% FBS in PBS) and incubated with primary labeled antibodies in FACS buffer for 1 h at 4 °C, as previously described^116^. After three washes, cells were resuspended in PBS, and 10,000 events were acquired on a LSRFortessa Cell Analyzer (BD Bioscience) and analyzed using FlowJo Software version 10.8.0 (FlowJo, LLC**)**.

Neutral lipid content was measured as described previously^34^. Briefly, cells in 6-well plates were washed with PBS and incubated in 2 μg/mL of BODIPY 493 in CM for 30 min at 37 °C. After washing with PBS, cells were harvested using trypsin and after three washes cells were resuspended in chilled PBS and mean intensities from 10,000 events were acquired.

Sphingolipid incorporation and accumulation in WT and ATG KO cells was assessed using BODIPY-labeled fluorescent probes, as described previously^117^. Briefly, WT and ATG KO cells were incubated at 4 °C for 30 min in Hanks’ Balanced Salt Solution (HBSS, pH 7.4) supplemented with 10 mM HEPES, along with 5 µM of either BODIPY FL C5-Sphingomyelin (Invitrogen, D3522) or BODIPY FL C5-Ceramide (Invitrogen, D3521), and 5 µM delipidated bovine serum albumin (BSA). Cells were washed with PBS and further incubated in serum-free medium at 37 °C for an additional 30 min. Cells were harvested using trypsin, and after three washes, cells were resuspended in chilled PBS, and intracellular mean fluorescence intensities from 10,000 events were acquired.

### TIRF imaging

TIRF imaging was performed on cells as described previously^49,118^. Briefly, cells were plated on 35 mm glass-bottom live imaging dishes (Mattek, P35G-1.5-14-C) and transfected with 500 ng of pCMV-Sport6-CD63-pHluorin (gift from D.M. Pegtel, Addgene, 130901) using Lipofectamine 2000 (Invitrogen, 11668019). After 24 h, cells were washed with Tyrode’s solution at 37 °C and imaged in the same buffer using a Nikon ECLIPSE Ti spinning disk confocal microscope. Images were acquired at 3 MHz for 300–600 s using a TIRF objective (60×, 1.45 N.A.) equipped with a high-speed EMCCD camera (iXon DU897, Andor) and NIS-Elements AR microscope imaging software. Fusion events were detected by sudden increase in fluorescence intensity on plasma membrane. Regions of interest were selected using rectangular selection tool and time series videos of 300 s were prepared using Fiji/ImageJ with frame rate/s of 25.

### Proteomic analysis on sEVs

Samples (30 μg of total protein per EV sample) were prepared for mass spectrometric analyses using the ThermoScientific EasyPep Mini MS Sample Pep Kit and essentially the manufacturer's recommended protocol. Protein digests were analyzed by LC-MS/MS on a Q Exactive Plus mass spectrometer (ThermoFisher Scientific) interfaced with a nanoAcquity UPLC system (Waters Corporation) equipped with a HSS T3 C18 column (75 µm × 150 mm; 1.8 µm particle size) (Waters Corporation) and a Symmetry C18 trapping column (180 µm × 20 mm; 5 µm particle size) (Waters Corporation) at a flow rate of 450 nL/min. The trapping column was positioned in line with the analytical column and upstream of a micro-tee union, which was used both as a vent for trapping and as a liquid junction. Trapping was performed using the initial solvent composition. Approximately 1 μg of peptide digest was injected onto the column. Peptides were eluted by using a linear gradient from 99% solvent A (0.1% formic acid in water (v/v)) and 1% solvent B (0.1% formic acid in acetonitrile (v/v)) to 40% solvent B over 100 min. For the mass spectrometry, a data-dependent acquisition method was employed with a dynamic exclusion time of 15 s and also exclusion of singly charged ions. The mass spectrometer was equipped with a NanoFlex source and a stainless-steel needle and was used in the positive ion mode. Instrument parameters were as follows: sheath gas, 0; auxiliary gas, 0; sweep gas, 0; spray voltage, 2.7 kV; capillary temperature, 275 °C; S-lens, 60; scan range (m/z) of 375 to 1500; 1.6 m/z isolation window; resolution: 70,000; automated gain control (AGC), 3 × 10^6 ^ions; and a maximum IT of 100 ms. For the MS/MS scans: TopN: 10; resolution: 17500; AGC 5 × 10^4^; maximum IT of 50 ms; and an (N)CE: 27. Mass calibration was performed before data acquisition using the Pierce LTQ Velos Positive Ion Calibration mixture (ThermoFisher Scientific). Data were processed using Proteome Discover (ThermoFisher Scientific) using a processing workflow that employed nodes for the Minora Feature Detector, Sequest HT, and Percolator. Data were searched against the appropriate database using Sequest and included settings of: trypsin specificity; allowance for two missed cleavages; 20 ppm mass tolerance for MS; 0.6 Da mass tolerance for MS/MS; static modification of cysteine residues with carbamidomethylation; variable methionine oxidation; and variable modification of asparagine and glutamine deamidation. A consensus workflow containing Feature Mapper and Precursor Ion Quantifier allowed for label-free quantification. Protein normalized abundance measurements were log_2_-transformed and median-normalized. A total of 1875 proteins were identified that met the criteria of observation in at least 2/3 of samples in at least one experiment condition (control or BafA1).

### Lipidomic analysis

#### Bligh and Dyer extraction

Each sEV sample pellet was resuspended to a volume of 160 μL in DI H_2_O, and cell pellets were resuspended in 320 μL. The Bligh and Dyer method was used to recover lipids from samples^119^. Briefly, methanol (Optima^TM^ LC-MS grade, Fisher Chemical) and chloroform (Optima^TM^ LC-MS grade, Fisher Chemical) were mixed together with the aqueous sample to achieve a final ratio of 2:1:0.8 methanol:chloroform:water. Samples were extracted for 1 h at RT in a rotary shaker at 1850 rpm. Subsequent to extraction, one volume equivalent of chloroform was added with brief mixing, followed by one volume equivalent of DI H_2_O to induce phase separation for a final volume of 2:2:1.8 methanol:chloroform:water. Samples were incubated by shaking at 1850 rpm, then centrifuged at 1100 × *g* for 20 min to aid the phase separation process. The lower (organic) phase was recovered and dried under a nitrogen stream (Labconco RapidVap Vertex Dry Evaporator). sEV samples were resuspended in 100 μL 4:4:1 methanol:2-propanol:choloroform, while cell pellets were resuspended in 125 μL.

#### Lipidomic via UHPLC-MS/MS

A Vanquish UHPLC system (ThermoFisher Scientific) was outfitted with an Accucore C30, 2.1 × 150 mm, 2.6 μm particle size column (ThermoFisher Scientific) and maintained at 40 °C. Autosampler was maintained at 15 °C. A binary gradient elution was carried out using solvents A (ACN:water, 60:40,v/v) and B (IPA:ACN, 90:10, v/v), both containing 10 mM ammonium formate and 0.1% formic acid. The column was equilibrated in 30% B with a flow rate of 350 μL/min. Following injection, the subsequent gradient was: 0–0.5 min isocratic elution with 30% B; 0.5–5.5 min, 30–43% B; 5.5–5.6 min, 43–50%; 5.6–14.5 min, 50–70% B; 14.5–21 min, 70–99% B; 21.5–24.5 min, 99% B; 24.5–24.6 min, 99–30%; 24.6–28.5 min, 30% B for column re-equilibration. The injection volume was 4 μL of each sample. A Q Exactive Plus quadrupole-Orbitrap mass spectrometer (ThermoFisher Scientific) was used for data collection. All MS experiments were performed in positive and negative ion modes using a HESI source, with the MS operated in data-dependent acquisition mode. MS conditions were set as follows: MS scan range: 150–1700 m/z; Resolution 35,000; Default Charge State 1; AGC target 3e6; Max IT 200 ms; microscans 1; Sheath gas 20; Aux gas 5 in positive mode and 7 in negative mode; Aux gas temp 375 °C; Sweep 1; Needle voltage 3 kV (both modes), Capillary temp 350 °C; S-lens 50. Note that Sheath/Aux/Sweep gas flow rates and S-lens settings are arbitrary units set in the instrument software. MS/MS mode was operated at resolution 17,500 in the Top10N DDA MS2 mode; AGC target 5e5; Max IT 100 ms; isolation window 1.0 m/z; HCD fragmentation with stepped normalized collision energy: 20, 24 and 28; Min AGC 8e3; dynamic exclusion set at 15.0 s.

#### Data analysis

Raw data files were processed using Compound Discoverer v3.3 (ThermoFisher Scientific) and searched against an in-house in silico-generated spectral library database. Mass tolerance was set to 5ppm for precursor, and the search algorithm HighChem HighRes was used. Retention times were aligned across the cohort using the ChromAlign node in the CD software. The lower retention limit for searches was set to 1.8 min, and the upper limit was 22 min. Subsequent to this feature finding, features were then exported to a text file and processed using in-house R scripts. Processing steps included comparison of m/z and retention time of annotated features versus an in-house generated list of m/z and retention time based on known lipid retention times, and assessment of signal variance in pooled QC samples versus sample cohort (i.e., dispersion ratio)^120^. Briefly, a pooled QC was utilized to calculate the dispersion ratio for the sample set as follows: statistical dispersion (variance of measurements) of the pooled QC samples in relation to the dispersion of the biological test samples was calculated in the form of the D-ratio. If the distribution of both the biological test sample measurements and the QC random error is Gaussian, then the dispersion ratio (D-ratio) is the ratio of the sample standard deviation for the pooled QC samples to the sample standard deviation for the biological test samples. If the data distribution is not Gaussian, then the raw data must be mathematically transformed. This plot of D-ratios reveals the value at which observed variance can be attributed to the measurement as opposed to being putatively biological in nature. Only features that passed all three criteria (spectral library match, retention time match, and D-ratio cutoff) were considered for semi-quantitative analysis.

### Lipid droplet analysis

Lipid droplet (LD) labeling was done as previously described^34^. Briefly, after 4% PFA fixation, cells were permeabilized and blocked with 0.1% saponin and 1% BSA. Cells were incubated with BODIPY 493 (2 μg/mL) in blocking buffer for 30 min at 37 °C. Cells were washed three times with PBS, and coverslips were mounted on glass slides using Fluor mount-G reagent with DAPI and imaged as described above. For LD quantification experiments, WT and ATG KO cells were plated in imaging plates (PhenoPlate 96-well, Revity, 6055302), incubated for 16 h at 37 °C in CM containing 1 μM Red C12. Cells were washed and fixed as described above. Nuclei were stained with DAPI, followed by imaging on an Opera Phenix Plus High-Content Screening System (Revvity, HH14001000). Five optical stacks of 1-µm intervals were acquired with a 40x water objective and lipid droplet parameters were analyzed using Harmony Software (Version 5.1). Cell boundaries were automatically identified by Harmony software. Lipid droplets were analyzed in the red channel (555 nm) as mean intensities and area per cell, and nuclei staining (405 nm) was used for quantitation for counting cell numbers.

### Triglyceride estimation assay

Triglycerides were measured using the Multi Lipid Detection Assay Kit (Signosis, EA-7013) as per the manufacturer’s protocol. Briefly, WT and ATG KO cells were detached with trypsin and centrifuged at 1000 *g* for 5 min, followed by two washes with cold PBS. Cell pellets were resuspended in 1 mL PBS and homogenized by sonication (Qsonica, Q500). Lipids were then extracted and phase separated by adding 2 mL chloroform and 1 mL methanol, followed by centrifugation at 1500 × *g* for 10 min. The lower chloroform phase was collected, vacuum dried, and reconstituted in an equal volume of PBS. TG concentrations were estimated from a standard curve prepared using the standadrs provided in the kit.

### RT-qPCR

Total RNA was extracted from WT or ATG KO cells using RNeasy Mini Kit (Qiagen, 74104), according to the manufacturer’s instructions. 1000 ng total RNA was utilized for cDNA synthesis using the iScript cDNA synthesis kit (Bio-Rad, 1708891). Real-time PCR was performed in triplicate using primer pairs specific for *ACSL4*, *ELOVL2*, *DGAT2*, *SMPD1*, *SGMS2*, *PISD*, as shown. CFX96 Real-Time Detector (Bio-Rad) was used to detect amplification using the fluorescent signal of SYBR Green by utilizing POWER SYBR Green PCR master kit (Applied Biosystems, 4367659). The human ACTB gene was used as an internal control for normalizing target gene mRNA levels. Primers were ordered from IDT (Integrated DNA Technologies) and Millipore-Sigma, and the primer sequences are listed in (Supplementary information Table 3). For analysis of lipid-metabolism enzymes, quantitative PCR was performed in technical triplicate using Human Fatty Acid and Lipid Metabolism Primer Library (HFLM-1; RealTimePrimers, LLC., Melrose Park, PA, USA) on QuantStudio 7 Flex Real-Time PCR System (Applied Biosystems) with 45 cycles at 94 °C for 15 s and 60 °C for 60 s.

### Statistical analysis

Data analysis and visualization were performed using GraphPad Prism version 10.1 (GraphPad Software). Results are presented as mean ± standard error of the mean (SEM). Statistical comparisons among multiple groups were performed using either one-way or two-way ANOVA, followed by Tukey’s post-hoc test as indicated in figure legends. All statistical tests were two-sided, and differences were considered statistically significant at *P* values of less than 0.05 (*), 0.01 (**), 0.001 (***), or 0.0001 (****). Exact *P* values are shown in each figure next to the corresponding asterisk, except for *P* < 0.0001, where only asterisks are presented for figure clarity.

Raw abundances output from Compound Discoverer for 'omics analysis were log₂-transformed and median-normalized across samples. Calculation of statistical significance in 'omics data sets employed the limma-3.56.2 package. Protein analysis incorporated a post-hoc adjustment performed by the DEqMS-1.18.0 package designed for mass spectrometry proteomics data that accounts for the number of peptide spectra matches for each protein.

Heatmaps were produced using the ComplexHeatmap-2.15.4 package^120^. Statistical hits were defined as experimental groups within each contrast having an adjusted p-value of <0.05 and an absolute fold change of ≥2.0 relative to one another.

### Reporting summary

Further information on research design is available in the Nature Portfolio Reporting Summary linked to this article.

## Supplementary information

Supplementary Info

Description of Additional Supplementary Files

Supplementary Data

Supplementary Movie 1

Supplementary Movie 2

Supplementary Movie 3

Supplementary Movie 4

Reporting Summary

Transparent Peer Review file

## Source data

Source Data

## Data Availability

The mass spectrometry data are publicly available at MassIVE with the accession numbers MSV000101801 (10.25345/C5FB4X13K) (proteomics) and MSV000101819 (10.25345/C53X84089) (lipidomics). All data and materials generated in this study are available from the corresponding author upon reasonable request. Source data are provided with this paper.
