# Supplementary material for: Lipid-manager autophagy proteins ATG2 and ATG9 regulate extracellular vesicle secretion via amphisome biogenesis and cell lipidome modulation
Source: Nat Commun. 2026 Jul 22;17:8948. doi: 10.1038/s41467-026-75742-x (PMC13503743; doi:10.1038/s41467-026-75742-x)
Supplement: Supplementary file 1 [file 41467_2026_75742_MOESM1_ESM.pdf]

Supplementary Information for:

Lipid-manager Autophagy Proteins ATG2 and ATG9 Regulate Extracellular Vesicles Secretion via Amphisome Biogenesis and Cell Lipidome Modulation

Asmita Singh[1], Dylan J. Johnson[2], Jeffrey Kuhn[3], Katina L. Johnson[3], Jason G. Williams[3], Ricardo Vancini[4], Deloris Sutton[4], Kedar Sharma[5], Jadranka Loncarek[6], Mario J. Borgnia[5], and Carlos M. Guardia[1*]

[1] Placental Cell Biology Group, National Institute of Environmental Health Sciences, National Institutes of Health, Research Triangle Park, North Carolina, USA.

[2] Integrative Bioinformatics Support Group, National Institute of Environmental Health Sciences, National Institutes of Health, Research Triangle Park, North Carolina, USA.

[3] Mass Spectrometry Research Center, National Institute of Environmental Health Sciences, National Institutes of Health, Research Triangle Park, North Carolina, USA.

[4] Pathology Support Group, Division of Translational Toxicology, National Institute of Environmental Health Sciences, National Institutes of Health, Research Triangle Park, North Carolina, USA.

[5] Molecular Microscopy Consortium, National Institute of Environmental Health Sciences, National Institutes of Health, Research Triangle Park, North Carolina, USA.

[6] Cancer Innovation Laboratory, Center for Cancer Research, National Cancer Institute, National Institutes of Health, Frederick, Maryland, USA.

*Corresponding author:

carlos.guardia@nih.gov

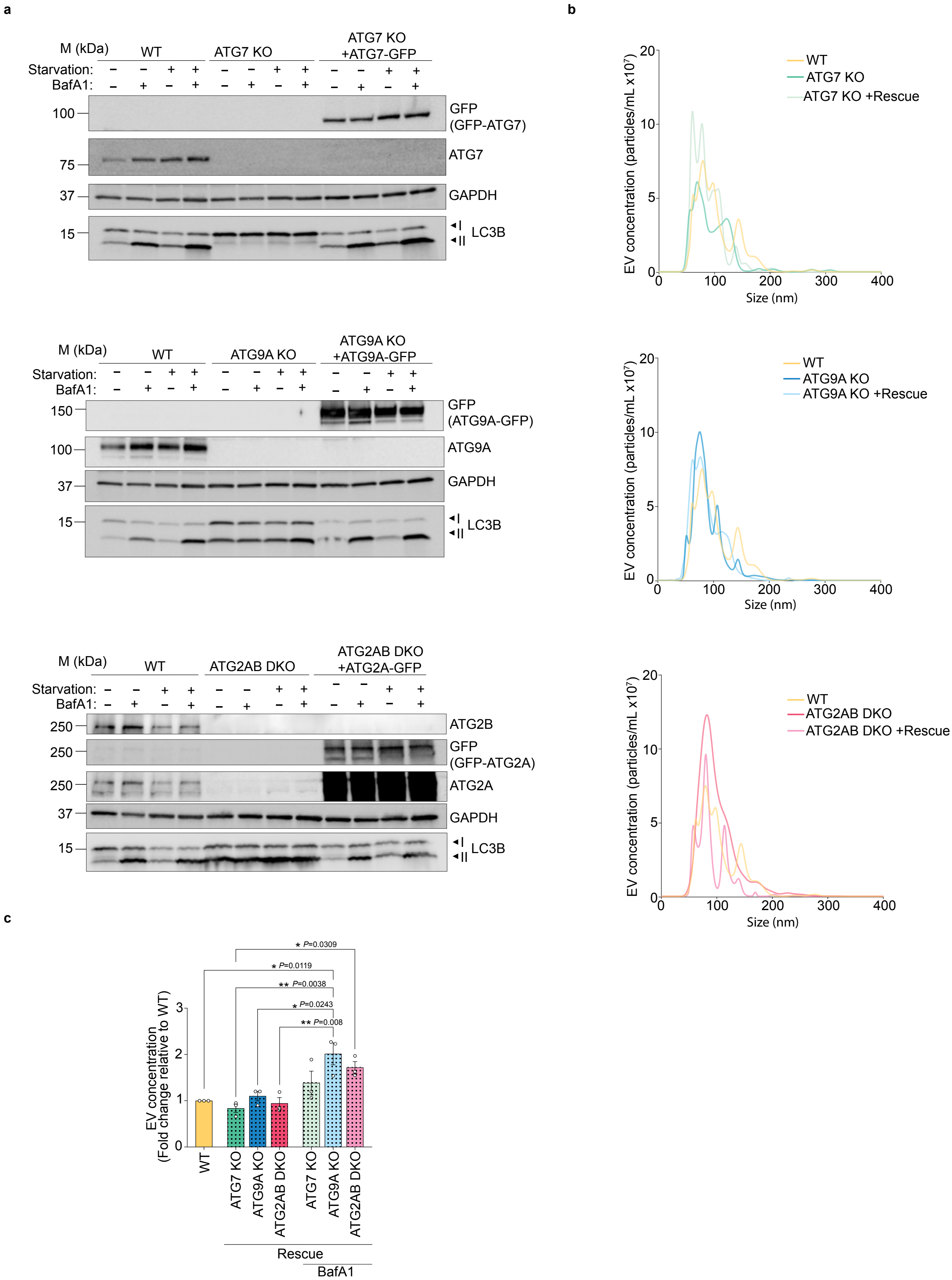
a
M (kDa)
WT
ATG7 KO
ATG7 KO +ATG7-GFP
Starvation:
BafA1:
100
75
37
15
GFP (GFP-ATG7)
ATG7
GAPDH
LC3B
I
II
ATG9A KO
ATG9A KO +ATG9A-GFP
150
GFP (ATG9A-GFP)
ATG9A
ATG2AB DKO
ATG2AB DKO +ATG2A-GFP
250
ATG2B
GFP (GFP-ATG2A)
ATG2A
b
WT
ATG7 KO
ATG7 KO +Rescue
ATG9A KO
ATG9A KO +Rescue
ATG2AB DKO
ATG2AB DKO +Rescue
EV concentration (particles/mL x10^7)
Size (nm)
c
EV concentration (Fold change relative to WT)
* P=0.0309
* P=0.0119
** P=0.0038
* P=0.0243
** P=0.008
WT
ATG7 KO
ATG9A KO
ATG2AB DKO
Rescue
BafA1

Supplementary Fig. 1 ATG KO cells do not show autophagy flux. a, IB analysis of lysates from WT, ATG KO and rescued cells after 3 h-treatment in control or starvation media (EBSS) with or without 100 nM BafA1. Membranes were probed with antibodies against GFP (for overexpressed constructs), LC3B, and the corresponding ATG protein, depending on the ATG KO studied in each blot. These are all indicated on the right of each blot. Molecular weight markers are indicated on the left side of each panel. GAPDH was used as loading control. **b,** Representative NTA profile of sEVs isolated from equal number of WT, ATG KO and rescued cells using size exclusion chromatography (SEC). **c,** Quantification (fold change relative to WT) of sEV concentration obtained from NTA analysis of n=3 biologically independent replicates of either control WT, ATG KO (as shown in **Fig. 1e**), and rescued cells or $BafA_1$ treated cells. sEV number is normalized to total cell count at the time of collection of conditioned media. Data is plotted as mean± SEM. Statistical significance was calculated using two-way ANOVA with Tukey's multiple comparisons test and only significant results are shown, * $P<0.05$, ** $P<0.01$.

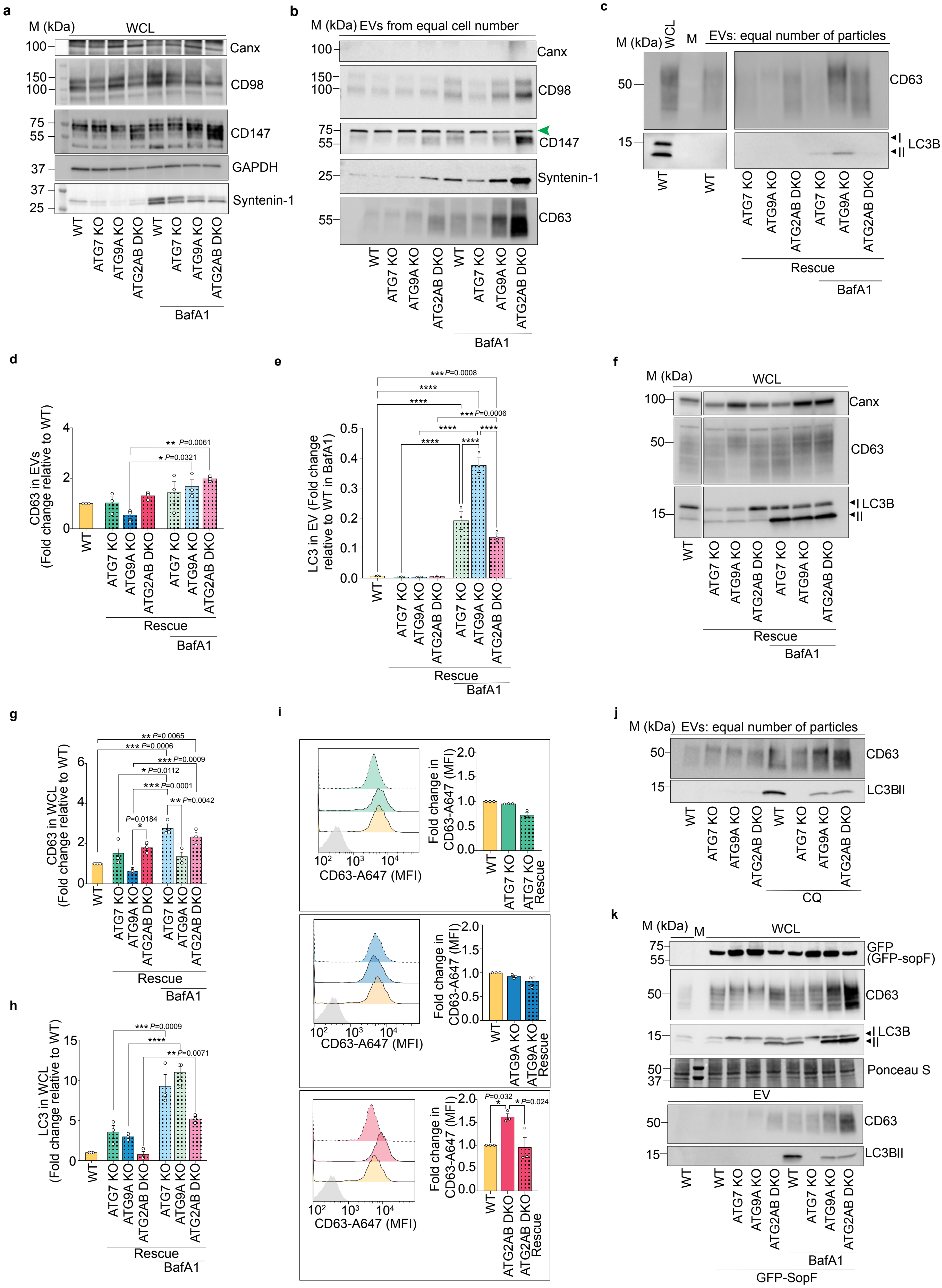
a
M (kDa)
WCL
Canx
CD98
CD147
GAPDH
Syntenin-1
WT
ATG7 KO
ATG9A KO
ATG2AB DKO
BafA1
b
M (kDa) EVs from equal cell number
Canx
CD98
CD147
Syntenin-1
CD63
BafA1
c
M (kDa)
WCL
M
EVs: equal number of particles
CD63
LC3B
Rescue
BafA1
d
CD63 in EVs
(Fold change relative to WT)
** P=0.0061
* P=0.0321
Rescue
BafA1
e
LC3 in EV (Fold change
relative to WT in BafA1)
*** P=0.0008
*** P=0.0006
Rescue
BafA1
f
M (kDa)
WCL
Canx
CD63
LC3B
Rescue
BafA1
g
CD63 in WCL
(Fold change relative to WT)
** P=0.0065
*** P=0.0006
*** P=0.0009
* P=0.0112
*** P=0.0001
** P=0.0042
P=0.0184
Rescue
BafA1
h
LC3 in WCL
(Fold change relative to WT)
*** P=0.0009
** P=0.0071
Rescue
BafA1
i
CD63-A647 (MFI)
Fold change in
CD63-A647 (MFI)
WT
ATG7 KO
ATG7 KO Rescue
ATG9A KO
ATG9A KO Rescue
P=0.032
* P=0.024
ATG2AB DKO
ATG2AB DKO Rescue
j
M (kDa) EVs: equal number of particles
CD63
LC3BII
CQ
k
M (kDa)
M
WCL
GFP
(GFP-sopF)
CD63
LC3B
Ponceau S
EV
CD63
LC3BII
BafA1
GFP-SopF

Supplementary Fig. 2 Intra and extracellular levels of EV marker CD63 are affected in ATG2 but not in ATG9A or ATG7 KO cells. a, b, IB of canonical sEV-associated exosome marker proteins, including CD63 and syntenin-1; the negative ER marker calnexin (Canx); and plasma membrane-derived ectosome-enriched proteins CD98 and CD147 (green arrowhead: unspecific band), in whole cell lysates from WT and ATGKO cells, and in sEVs derived from an equal number of WT and ATGKO cells under control condition or after 16 h treatment with 100 nM $BafA_1$. **c,** IB analysis of exosomal marker CD63 and autophagy marker LC3B(-II) on equal number of sEVs particles isolated via SEC obtained from either control WT, as shown in **Fig. 2a**, and ATG KO rescued cells treated for 16 h with 100 nM of $BafA_1$. Representative IB image probed with antibodies as indicated on the right. Molecular weight markers are indicated on the left side of panel. **d, e,** Quantification of the levels of sEVs-associated (d) CD63 and (e) LC3B from ATG KO cells under control or $BafA_1$ treatment conditions, as shown in panel (c). Individual data points are marked as open circles. n=3 independent biological replicates. Bars represent the mean± SEM of fold change in sEVs-associated CD63 and LC3. Statistical significance was calculated using two-way ANOVA with Tukey's multiple comparisons test. * $P< 0.05$, ** $P<0.01$, *** $P<0.001$, **** $P<0.0001$. **f,** IB analysis of the indicated proteins in whole cell lysates of WT, ATGKO cells, as shown in **Fig. 2d**, and rescued cells under control or $BafA_1$ treatment. Representative IB image probed using antibodies as indicated on the right. Molecular weight marker is indicated in the left side of panel**. g, h,** Quantification of intracellular CD63 and LC3B-II/I levels as the fold change relative to that of control WT cells from independent experiments, as shown in panel f**.** Individual data points are marked as open circles. n=3 independent biological replicates. Bars represent the mean ± SEM of fold change in CD63 and LC3 protein levels in whole cell lysates. Statistical significance was calculated using two-way ANOVA with Tukey's multiple comparisons test. * $P<0.05$, ** $P<0.01$, *** $P<0.001$. **i,** Representative flow cytometry overlaid histograms of total intracellular CD63 levels in WT, ATG KO and rescued cells, fixed and permeabilized, followed by incubation incubated with either Alexa Fluor-647-conjugated CD63 antibody and its isotype control immunoglobin (IgG- Alexa Fluor-647). Mean fluorescence intensities were analyzed using FlowJo. n=10,000. The quantification of CD63-MFI is plotted as fold change with respect to WT cells from three independent experiments. Statistical significance was calculated using one-way ANOVA with Tukey's multiple comparisons

test * $P<0.05$. **j,** IB analysis of exosomal marker CD63 and autophagy marker LC3B(-II) on equal number of sEVs particles isolated via SEC obtained from either control WT, ATG KO cells, or cells treated for 16 h with 50 μM chloroquine (CQ). Representative IB image out of n=2 replicates probed using antibodies as indicated on the right. Molecular weight markers are indicated on the left side of panel. **k,** IB analysis of the exosomal marker protein CD63 and the autophagy marker LC3B in whole cell lysates (WCL) and sEVs. The first lane contains WCL from WT cells, followed by WT and ATG KO cells stably expressing GFP-SopF, each under either control or BafA1 treatment. Data are representative of a single experiment. The positions of molecular mass markers (M; second lane), in kDa, are indicated on the left.

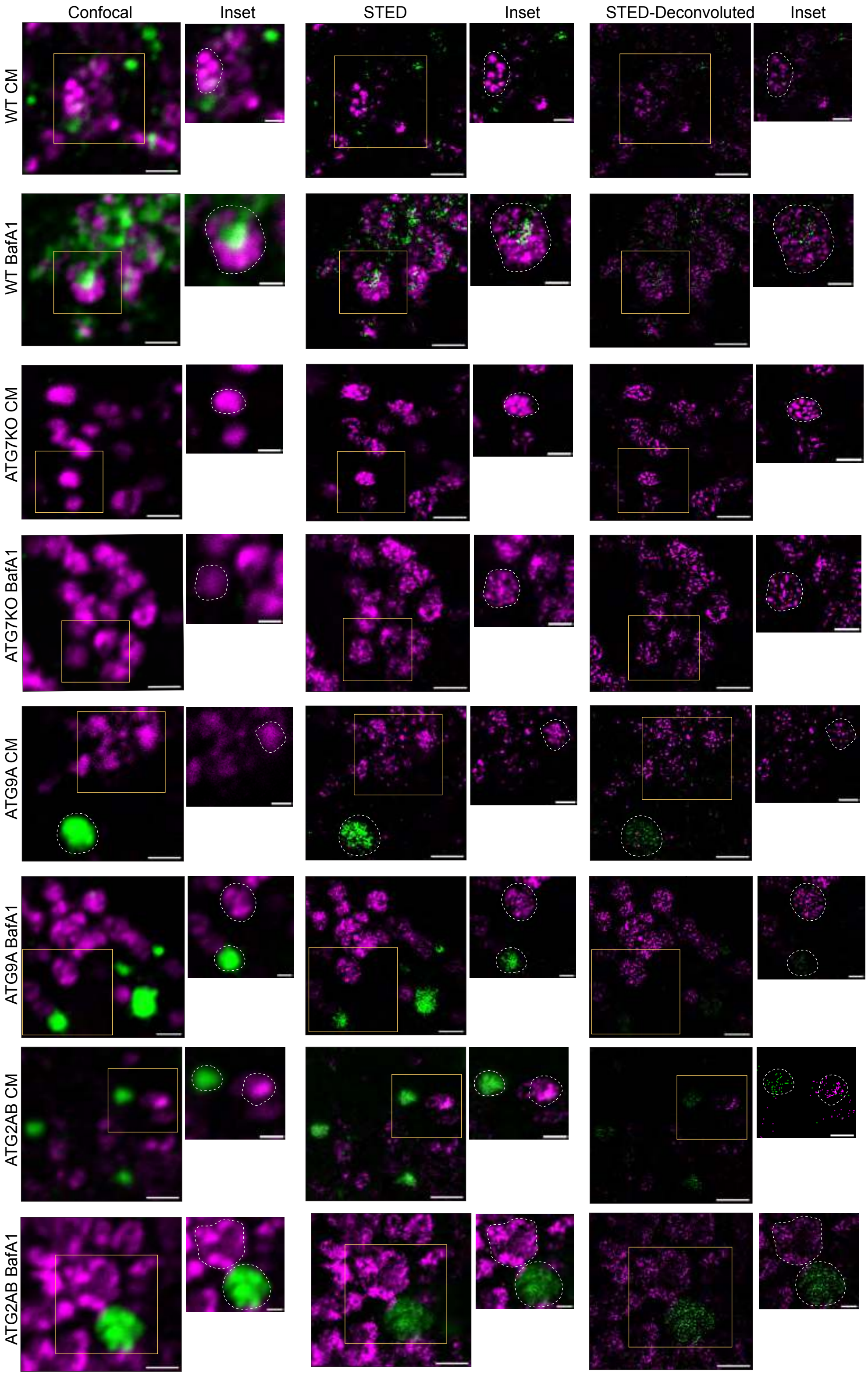

Supplementary Fig. 3 Amphisome formation is disrupted in autophagosome-deficient cells. Representative confocal, STED, and deconvoluted STED images of endogenous CD63 and LC3B staining in WT and KO cells cultured in CM and $BafA_1$ conditions. Magnification of yellow boxed regions are shown to the right as inset. Scale bar=1 μm; inset image scale bar=0.5 μm. White dashed lines represent visually identifiable CD63 or LC3-positive compartments.

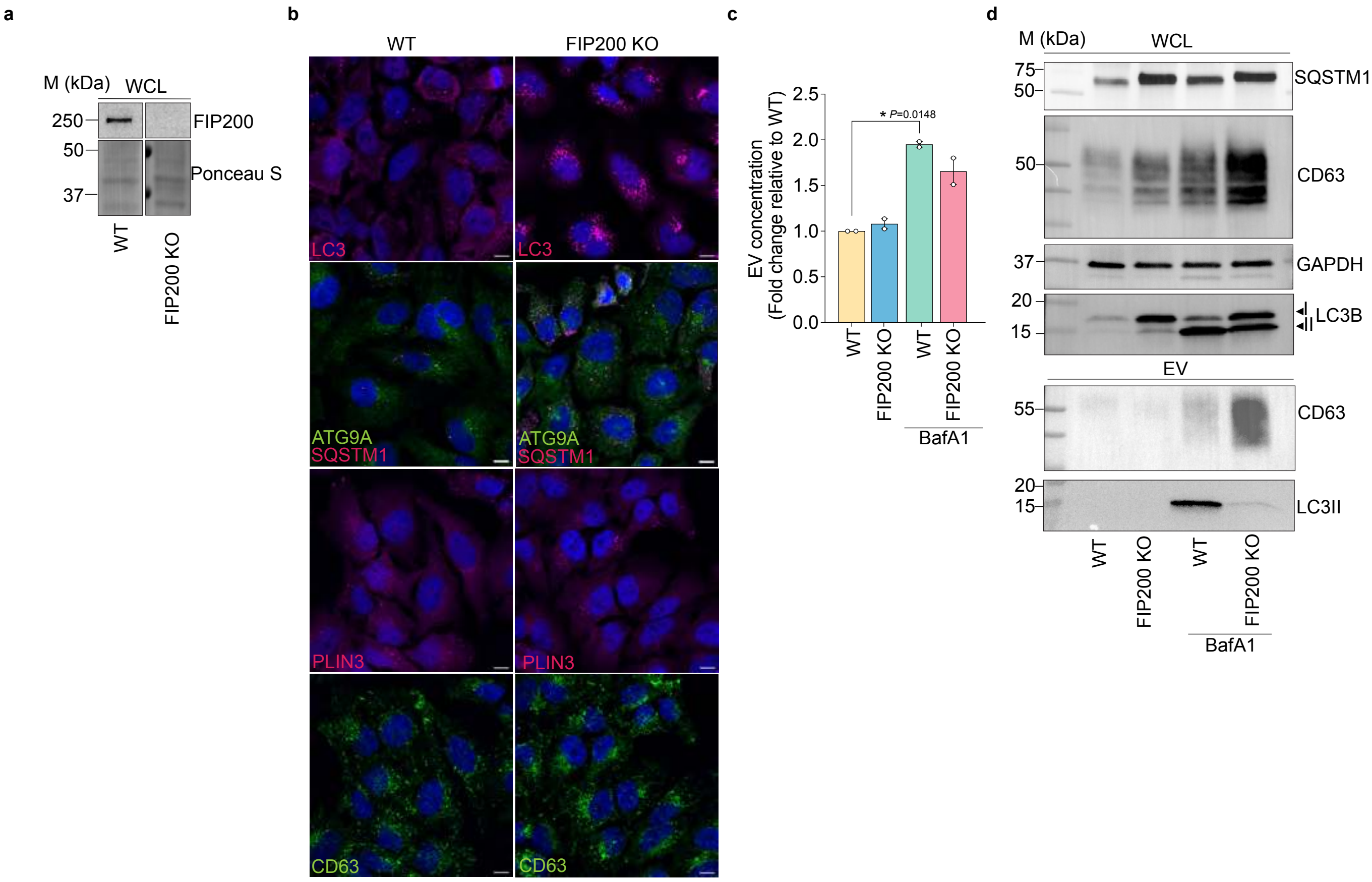

Supplementary Fig. 4 Distinct effects of FIP200 KO on EV secretion and cargo compared to ATG9A and ATG2AB DKO cells. a, Immunoblot (IB) analysis of lysates from WT and FIP200 KO cells, using antibody as indicated on the right. The positions of molecular mass markers (in kDa) are indicated on the left. Ponceau S staining was used as loading control. **b,** Representative confocal images of PLIN3/TIP47 (magenta), CD63 (green), LC3 puncta (magenta), ATG9A (green) and p62/SQSTM1 (magenta) in WT and FIP200 KO cells. Nuclei were stained with DAPI (blue). Scale bar=10 µm. **c,** Quantification (fold change relative to WT) of sEV concentration obtained from NTA analysis of two biologically independent replicates of either control WT and FIP200 KO or $BafA_1$ treated cells. sEV number is normalized to total cell count at the time of collection of conditioned media. Data is plotted as mean± SEM. Statistical significance was calculated using two-way ANOVA with Tukey's multiple comparisons test and only significant results are shown, * $P<0.05$. **d,** IB analysis of exosomal marker CD63 and autophagy marker LC3B in whole cell lysates or on equal number of sEVs particles isolated via SEC from either control WT or FIP200 KO cells treated for 16 h with 100 nM of $BafA_1$. Representative IB image probed with antibodies as indicated on the right. Molecular weight markers are indicated on the left side of panel.

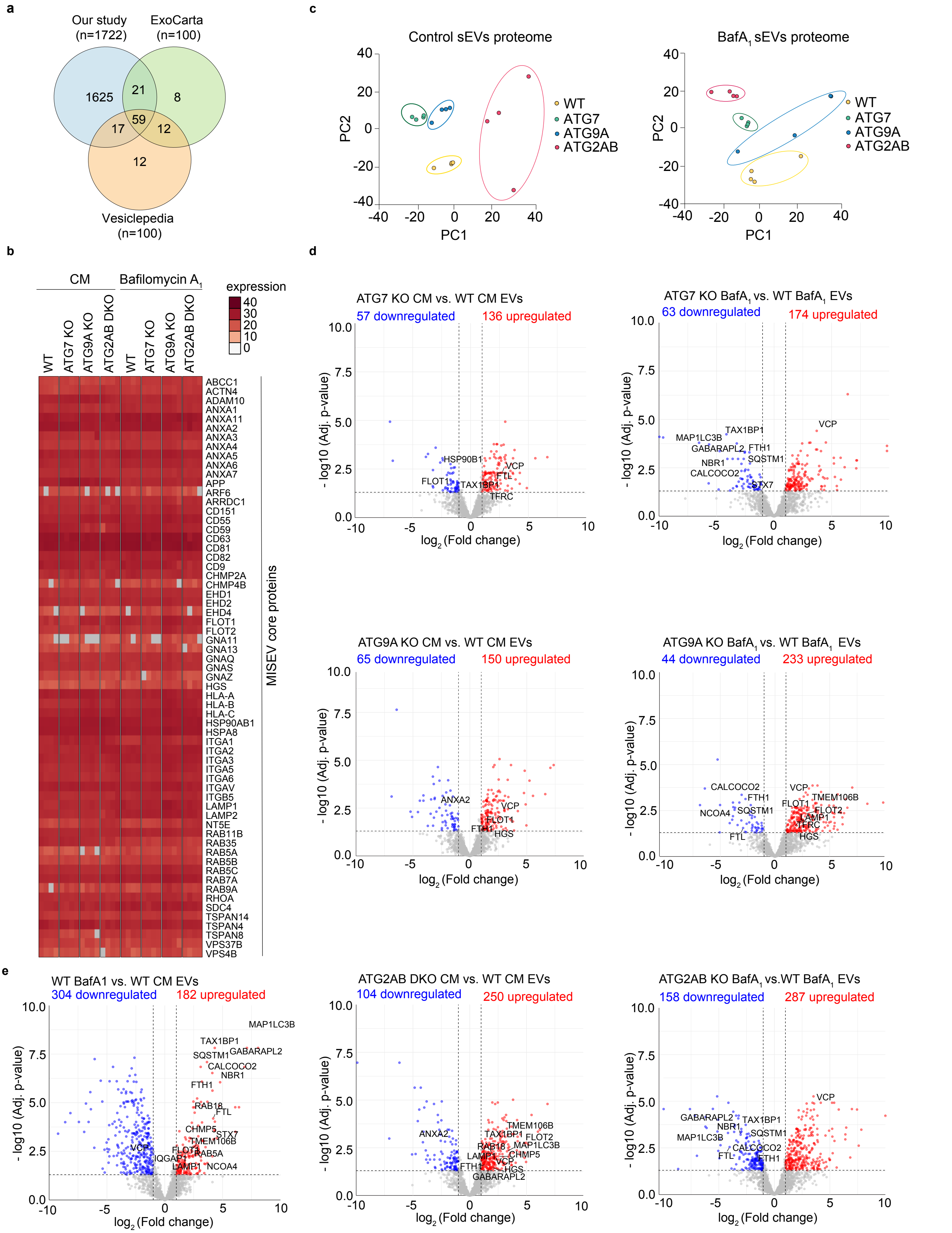
a
Our study (n=1722)
ExoCarta (n=100)
Vesiclepedia (n=100)
1625
21
8
59
17
12
12
b
CM
Bafilomycin A1
expression
40
30
20
10
0
WT
ATG7 KO
ATG9A KO
ATG2AB DKO
MISEV core proteins
ABCC1
ACTN4
ADAM10
ANXA1
ANXA11
ANXA2
ANXA3
ANXA4
ANXA5
ANXA6
ANXA7
APP
ARF6
ARRDC1
CD151
CD55
CD59
CD63
CD81
CD82
CD9
CHMP2A
CHMP4B
EHD1
EHD2
EHD4
FLOT1
FLOT2
GNA11
GNA13
GNAQ
GNAS
GNAZ
HGS
HLA-A
HLA-B
HLA-C
HSP90AB1
HSPA8
ITGA1
ITGA2
ITGA3
ITGA5
ITGA6
ITGAV
ITGB5
LAMP1
LAMP2
NT5E
RAB11B
RAB35
RAB5A
RAB5B
RAB5C
RAB7A
RAB9A
RHOA
SDC4
TSPAN14
TSPAN4
TSPAN8
VPS37B
VPS4B
c
Control sEVs proteome
BafA1 sEVs proteome
PC1
PC2
WT
ATG7
ATG9A
ATG2AB
d
ATG7 KO CM vs. WT CM EVs
57 downregulated
136 upregulated
ATG7 KO BafA1 vs. WT BafA1 EVs
63 downregulated
174 upregulated
ATG9A KO CM vs. WT CM EVs
65 downregulated
150 upregulated
ATG9A KO BafA1 vs. WT BafA1 EVs
44 downregulated
233 upregulated
- log10 (Adj. p-value)
log2 (Fold change)
e
WT BafA1 vs. WT CM EVs
304 downregulated
182 upregulated
ATG2AB DKO CM vs. WT CM EVs
104 downregulated
250 upregulated
ATG2AB KO BafA1 vs.WT BafA1 EVs
158 downregulated
287 upregulated

Supplementary Fig. 5 Comparative analysis of proteins found withing sEVs from WT and ATG KO cells under control and lysosomal inhibition conditions. a, Venn diagram depicting the overlap of total number of differentially abundant proteins (EVs from ATG KO vs WT cells; $\log_2$ fold change >1 ; P-value < 0.05) from our study with proteins quantified among different databases included in the ExoCarta (http://www.exocarta.org/) and Vesiclepedia (http://www.microvesicles.org/). **b,** Heat map showing the abundance of enriched proteins in EVs from all the samples detected and listed according to *MISEV* guidelines. **c,** Two-dimensional PCA plots of the total proteins quantified in EVs from control and BafA1 treated condition. **d,e,** Volcano plots depicting differentially expressed genes described in **Fig 4a**. y axis depicts $-\log_{10}$(adjusted p -value), while x axis depicts $\log_2$(fold change) abundance.

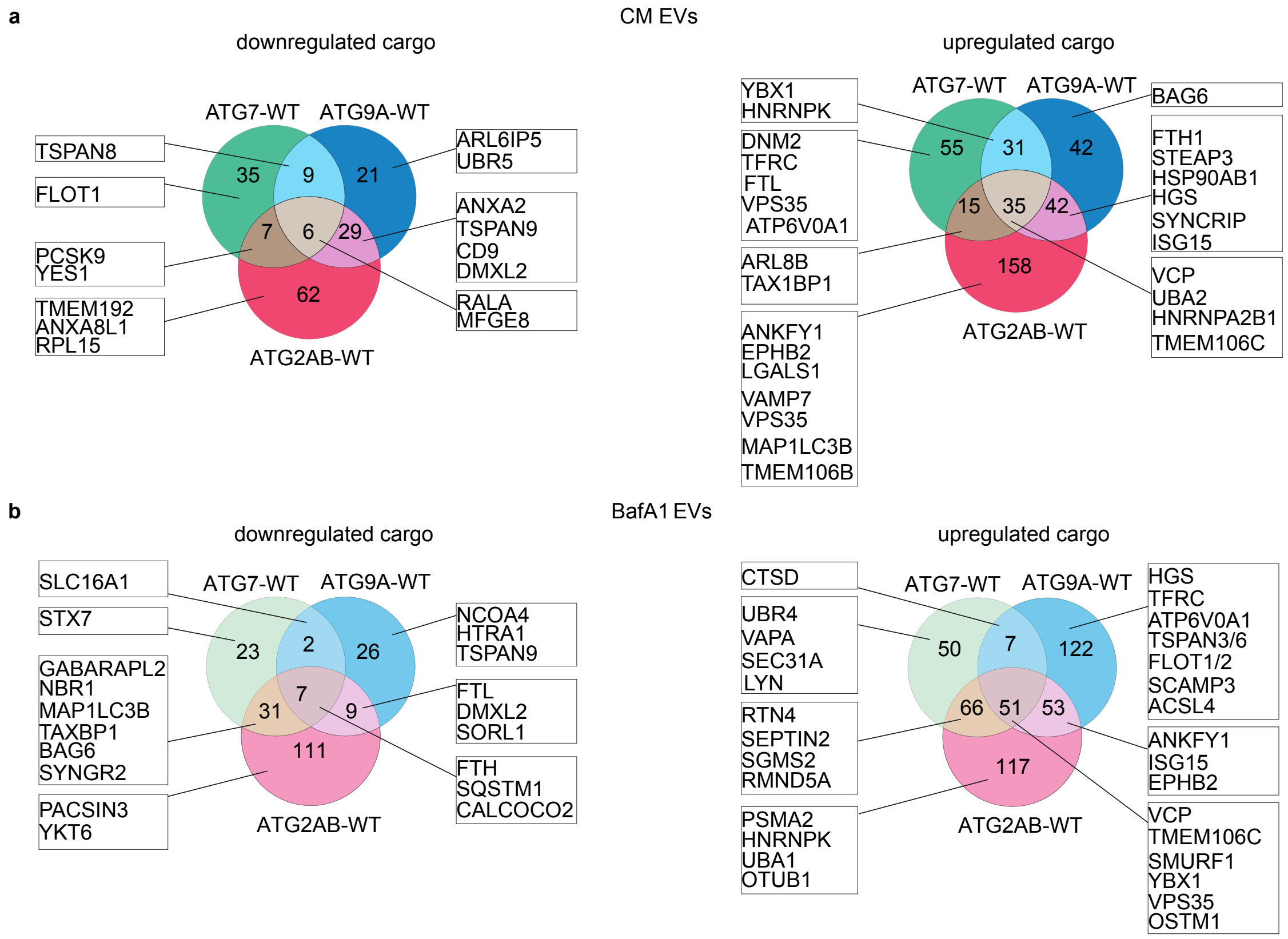

Supplementary Fig. 6 sEVs from WT and ATG KO cells exhibit distinct cargo with overlapping enrichment within sEVs from ATG KO cells. a-b, Venn diagrams illustrating the overlap of upregulated or downregulated cargo in ATG KO cells compared to WT cells under control (**a**) or BafA1 treatment (**b**) conditions. Selected proteins reported as autophagy regulators, components of EVs, or involved in EV secretion are shown in boxes.

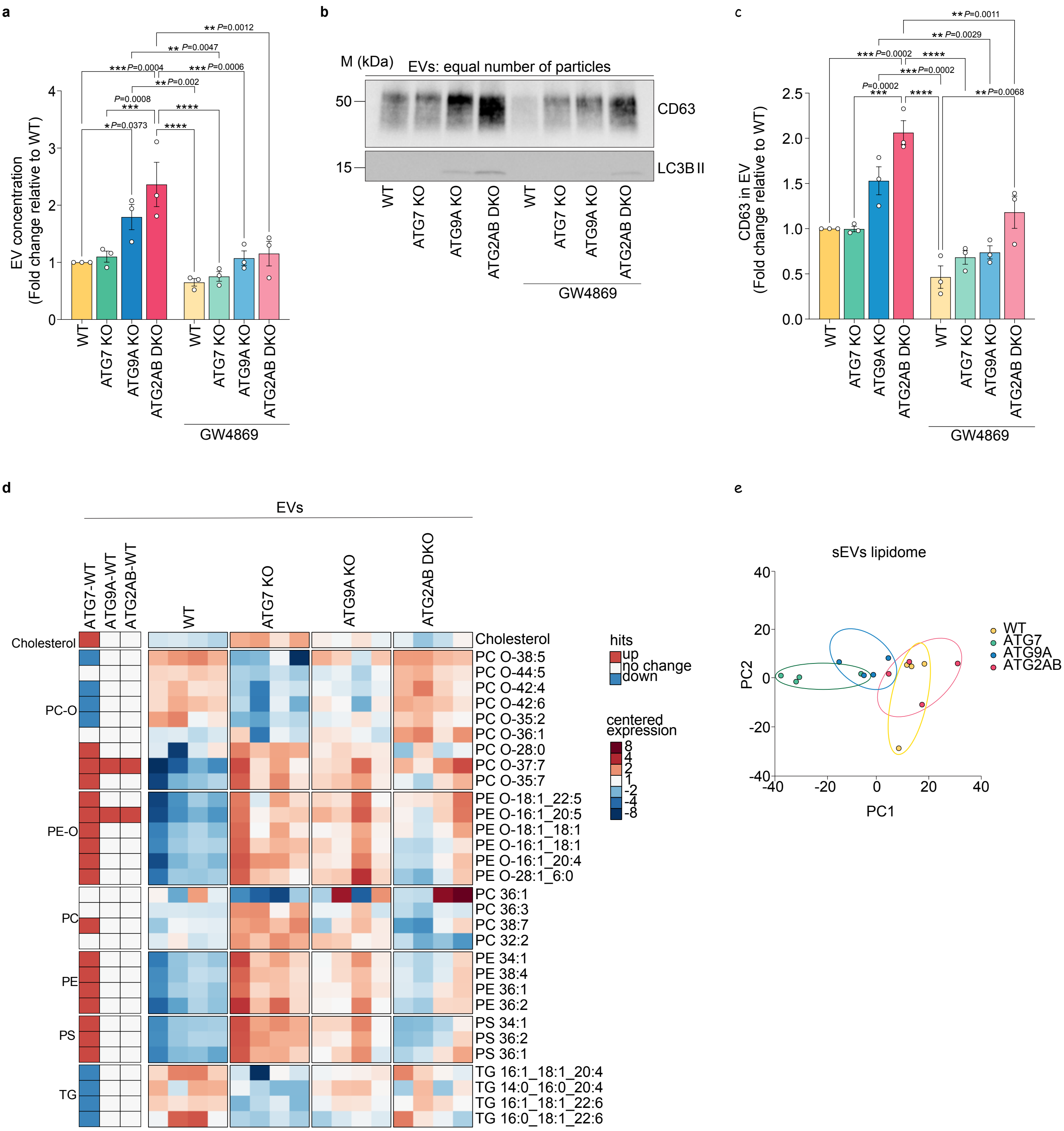
a
EV concentration
(Fold change relative to WT)
WT
ATG7 KO
ATG9A KO
ATG2AB DKO
GW4869
b
M (kDa)
EVs: equal number of particles
50
15
CD63
LC3B II
c
CD63 in EV
(Fold change relative to WT)
d
EVs
ATG7-WT
ATG9A-WT
ATG2AB-WT
Cholesterol
PC-O
PE-O
PC
PE
PS
TG
hits
up
no change
down
centered expression
PC O-38:5
PC O-44:5
PC O-42:4
PC O-42:6
PC O-35:2
PC O-36:1
PC O-28:0
PC O-37:7
PC O-35:7
PE O-18:1_22:5
PE O-16:1_20:5
PE O-18:1_18:1
PE O-16:1_18:1
PE O-16:1_20:4
PE O-28:1_6:0
PC 36:1
PC 36:3
PC 38:7
PC 32:2
PE 34:1
PE 38:4
PE 36:1
PE 36:2
PS 34:1
PS 36:2
PS 36:1
TG 16:1_18:1_20:4
TG 14:0_16:0_20:4
TG 16:1_18:1_22:6
TG 16:0_18:1_22:6
e
sEVs lipidome
PC1
PC2
WT
ATG7
ATG9A
ATG2AB

Supplementary Fig. 7 Lipidome analysis of sEVs derived from WT and ATG KO cells. a, Quantification of total EV concentration from indicated cells. n=3 biologically independent replicates in control medium and GW4869-treated cells. Data is plotted as mean ± SEM. Statistical significance was calculated using two-way analysis of variance (ANOVA) with Tukey's multiple comparisons test. Only significant results are shown for clarity, * *P*<0.05, ** *P*<0.01, *** *P*<0.001, **** *P*<0.0001. **b**, IB analysis of exosomal marker protein CD63 and LC3B-II on equal number of isolated EVs. The positions of molecular mass markers (in kDa) are indicated on the left. **c,** Quantification of CD63 in EVs from panel (a**).** Individual data points are marked as open circles from three independent biological replicates. Bars represent the mean ± SEM of fold change. Statistical significance was calculated using two-way ANOVA with Tukey's multiple comparisons test, ** *P*<0.01, *** *P*<0.001, **** *P*<0.0001. **d,** Heatmap showing the abundance of lipid species enriched in EVs. n= 4 independent replicates. Each rectangle represents a lipid species which is colored by its centered expression intensity scale from blue (decreased) to red (increased). CHOL, cholesterol; PC-O, ether-linked phosphatidylcholines; PE-O, ether-linked phosphatidylethanolamines; PC, phosphatidylcholines; PE, phosphatidylethanolamines PS, phosphatidylserines; TG, triacylglycerols. **e,** PCA plot of the lipidomic data from WT and ATG KO EVs. Data from the same biological replicate as in panel (d).

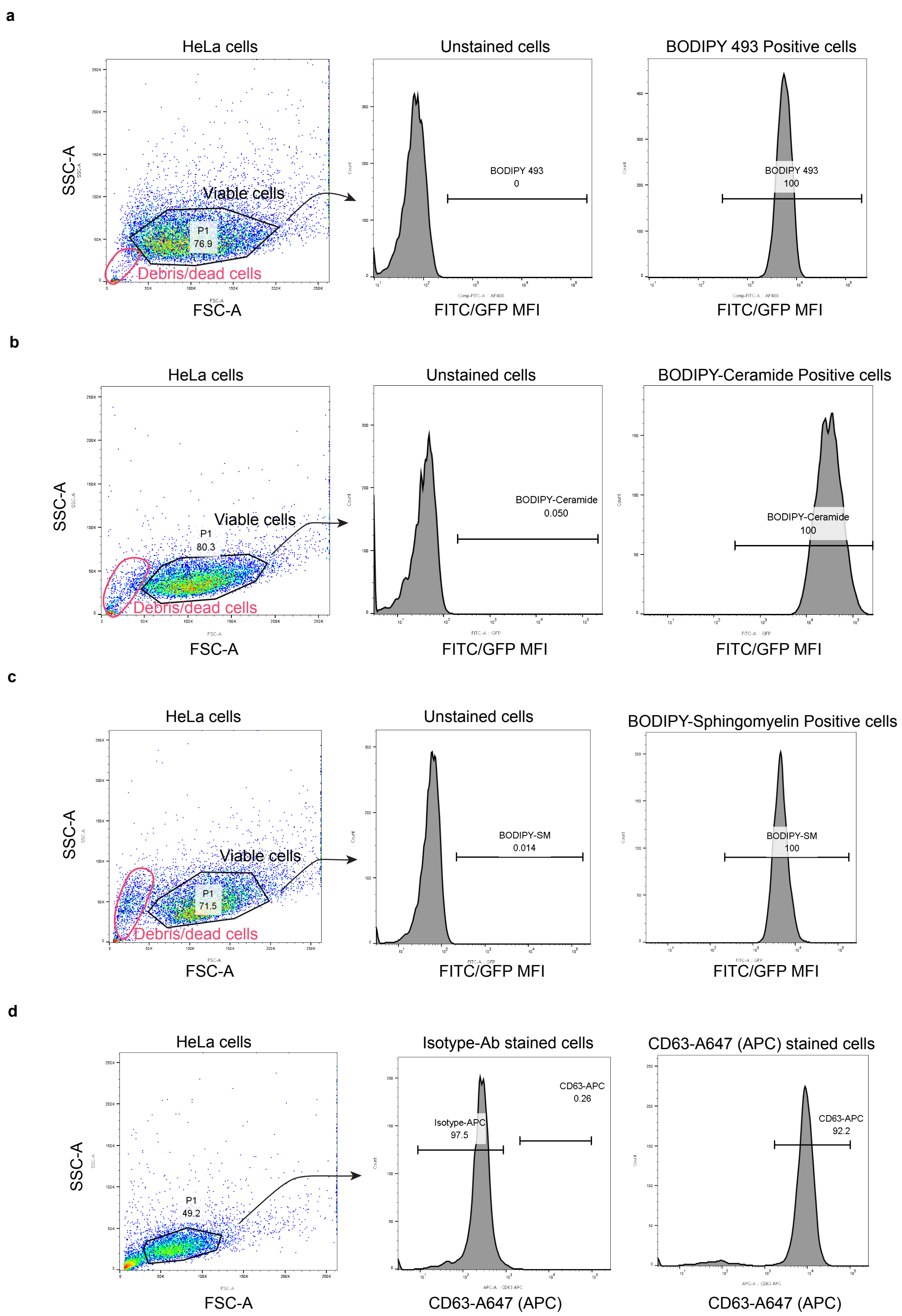
a
HeLa cells
SSC-A
FSC-A
Viable cells
P1
76.9
Debris/dead cells
Unstained cells
BODIPY 493
0
FITC/GFP MFI
BODIPY 493 Positive cells
BODIPY 493
100
FITC/GFP MFI
b
HeLa cells
SSC-A
FSC-A
Viable cells
P1
80.3
Debris/dead cells
Unstained cells
BODIPY-Ceramide
0.050
FITC/GFP MFI
BODIPY-Ceramide Positive cells
BODIPY-Ceramide
100
FITC/GFP MFI
c
HeLa cells
SSC-A
FSC-A
Viable cells
P1
71.5
Debris/dead cells
Unstained cells
BODIPY-SM
0.014
FITC/GFP MFI
BODIPY-Sphingomyelin Positive cells
BODIPY-SM
100
FITC/GFP MFI
d
HeLa cells
SSC-A
FSC-A
P1
49.2
Isotype-Ab stained cells
Isotype-APC
97.5
CD63-APC
0.26
CD63-A647 (APC)
CD63-A647 (APC) stained cells
CD63-APC
92.2
CD63-A647 (APC)

Supplementary Fig. 8 Gating strategy for analysis of neutral lipids, sphingolipid internalization, and intracellular CD63 levels in WT, ATGKO and Rescued cells. a-c, Gating strategy for measurement of the neutral lipid levels and sphingolipid internalization in WT and ATG KO cells. Cells were stained as described in Methods section. Unstained cells (without BODIPY) were used to set FSC-A/SSC-A gate and BODIPY negative gate for MFI. Viable cells were gated (P1) on SSC-A and FSC-A plot. These gated cells were analyzed for BODIPY signal in GFP/FITC channel in flow cytometer. 10,000 cells were acquired on P1 gate for all replicates. **d,** Gating strategy for measurement of intracellular CD63 levels. Cells were stained as described in Methods section. Unstained cells were used to set FSC-A/SSC-A gate and APC/Alexa 647 negative gate for MFI. Isotype control (IgG-APC) was used for each cell type analyzed. 10,000 cells were acquired on P1 gate for all replicates. The figures showing gating strategy here are representative of one cell type and biological replicate.

Supplementary Table 1. sgRNA used in the study.

Target	sgRNA targeting sequence:	Source
ATG9A	5'-CTATAGTGATTCACCCCCAG-3'	This study
ATG7	5'-GAACTGCAGTTTAGAGAGTC-3'	Previous study (Mailler et al., 2021)
ATG2A	5'-GCTCAGCCTCGATCTGTACA-3'	Previous study (Mailler et al., 2021)
ATG2B	5'-CCTCTGCAGGAGGTACCGGC-3'	Previous study (Mailler et al., 2021)
FIP200	5'-AGAGTGTGTACCTACAGTGC-3'	This study

Supplementary Table 2. shRNA vectors for generating HeLa lentiviral stable cell lines.

Cell line	Target gene	shRNA	ShRNA targeting sequence
HeLa shControl	Non-target	shControl	5'-ATCTCGCTTGGGCGAGAGTAAG-3'
HeLa shRAB27A	*RAB27A*	ShRAB27A	5'-GGGAAGACCAGTGTACTTT-3'

Supplementary Table 3. RT-PCR Primers used in the study.

Target	Forward-primer	Reverse-primer
ACSL4	5'-TGCAGCACAGACCTGCTTTA-3'	5'-TGCTTCTTTGCCAAGTGTGG-3'
ELOVL2	5'-ACTTCTCTCCGCGTACATGC-3'	5'-CACCAAAGCACCTTGGCTAC-3'
DGAT2	5'-CTGGGAGATGGGGAGTGGC-3'	5'-ACACAGCCCAGTTTCGGAC-3'
SMPD1	5'-CCTCAGAATTGGGGGGTTCTATGC-3'	5'-AGCCACTGGAGCTGTCCT-3'
SGMS2	5'-CGGTTACGCTGACACTGACT-3'	5'-AGAATGCAGATGATCCCGGC-3'
PISD	5'-GCAACCTCAGCGAGTTCTTC-3'	5'-CCTTTACCTGCTCCACCTCA-3'
ACTB	5'-GCACTCTTCCAGCCTTCCTT-3'	5'-CGGATGTCCACGTCACACTT-3'
GAG	5'-GGAGCTAGAACGATTCGCAGTTA-3'	5'-GGTTGTAGCTGTCCCAGTATTTGTC-3'
