# Supplementary material for: Lipid-manager autophagy proteins ATG2 and ATG9 regulate extracellular vesicle secretion via amphisome biogenesis and cell lipidome modulation
Source: Nat Commun. 2026 Jul 22;17:8948. doi: 10.1038/s41467-026-75742-x (PMC13503743; doi:10.1038/s41467-026-75742-x)
Supplement: Supplementary file 2 [file 41467_2026_75742_MOESM2_ESM.pdf]

Title: Supplementary Data 1

Description: EV proteomic analysis. Generated for Figure 4a-b and Supplementary Fig. 3c.

Title: Supplementary Data 2

Description: Enrichment of cargo receptors. Generated for Figure 4c.

Title: Supplementary Data 3

Description: Whole cell lipidome analysis. Generated for Fig. 5a.

Title: Supplementary Data 4

Description: EV lipidome analysis. Generated for Supplementary Fig. 4d,e.

Title: Supplementary Data 5

Description: qPCR analysis for differential expression of fatty acid metabolic genes using Human Fatty Acid and Lipid Metabolism Primer Library. Generated for Supplementary Fig. 6a,b.

Title: Supplementary Movies 1-4

Description: Live-cell TIRF imaging of CD63-pHluorin expressing WT (Supplementary Movie 1), ATG7KO (Supplementary Movie 2), ATG9A (Supplementary Movie 3) and ATG 2AB DKO (Supplementary Movie 4) cells were imaged in control medium, showing fusion events over a time interval up to 300 s with frame rate/s of 25 as shown in Figure 3. Scale bar=10 μm.
