# Supplementary material for: Lipid-manager autophagy proteins ATG2 and ATG9 regulate extracellular vesicle secretion via amphisome biogenesis and cell lipidome modulation
Source: Nat Commun. 2026 Jul 22;17:8948. doi: 10.1038/s41467-026-75742-x (PMC13503743; doi:10.1038/s41467-026-75742-x)
Supplement: Supplementary file 3 [file 41467_2026_75742_MOESM8_ESM.pdf]

nature portfolio

Corresponding author(s): Carlos M. Guardia

Last updated by author(s): May 19, 2026

Reporting Summary

Nature Portfolio wishes to improve the reproducibility of the work that we publish. This form provides structure for consistency and transparency in reporting. For further information on Nature Portfolio policies, see our Editorial Policies and the Editorial Policy Checklist.

Statistics

For all statistical analyses, confirm that the following items are present in the figure legend, table legend, main text, or Methods section.

n/a	Confirmed	
[ ]	[x]	The exact sample size (n) for each experimental group/condition, given as a discrete number and unit of measurement
[ ]	[x]	A statement on whether measurements were taken from distinct samples or whether the same sample was measured repeatedly
[ ]	[x]	The statistical test(s) used AND whether they are one- or two-sided<br>*Only common tests should be described solely by name; describe more complex techniques in the Methods section.*
[x]	[ ]	A description of all covariates tested
[ ]	[x]	A description of any assumptions or corrections, such as tests of normality and adjustment for multiple comparisons
[ ]	[x]	A full description of the statistical parameters including central tendency (e.g. means) or other basic estimates (e.g. regression coefficient) AND variation (e.g. standard deviation) or associated estimates of uncertainty (e.g. confidence intervals)
[ ]	[x]	For null hypothesis testing, the test statistic (e.g. F, t, r) with confidence intervals, effect sizes, degrees of freedom and P value noted<br>*Give P values as exact values whenever suitable.*
[x]	[ ]	For Bayesian analysis, information on the choice of priors and Markov chain Monte Carlo settings
[x]	[ ]	For hierarchical and complex designs, identification of the appropriate level for tests and full reporting of outcomes
[x]	[ ]	Estimates of effect sizes (e.g. Cohen's d, Pearson's r), indicating how they were calculated

Our web collection on statistics for biologists contains articles on many of the points above.

Software and code

Policy information about availability of computer code

Data collection	For cell count: Cellometer Auto T4 system v 3.3.9.5. (Nexcelom Bioscience)<br>For WB gel imaging: ChemiDoc MP Imaging System v 3.0.1.14 (Bio-Rad)<br>For Nanoparticles acquisition: Nanosight NS300 (Malvern Panalytical)<br>For CryoEM grid preparation: Leica EM GP2 environmental chamber (Leica Microsystems)<br>For CryoEM data acquisition: 200 kV Talos Arctica microscope (Thermo Fisher Scientific)<br>For TEM grid preparation: EM TP tissue processor (Leica)<br>For TEM data acquisition: JEM-1400plus Electron Microsope (JEOL)<br>For Confocal microscopy imaging: ZEN Black v 2.3 (Zeiss) and Opera Phenix Plus High-Content Screening System (Revvity)<br>For STED imaging: STEDYCON v 10.2.1622 (Abberior Instruments)<br>For STED image deconvolution: Huygens software v 25.10 (Scientific Volume Imaging)<br>FOR TIRF acquisition: Nikon ECLIPSE Ti spinning disk confocal microscope v Fusion 2.4.0.14 (Nikon microscopes)<br>For FACS acquisition: LSR Fortessa v 8.0.3 (BDbiosciences)<br>For FACS sorting: FACSymphony S6 v 9.9 and v 9.5.1 (BDbiosciences)<br>For Mass spectrometry data acquisition: Q Exactive™ Plus Hybrid Quadrupole-Orbitrap™ Mass Spectrometer v 2.8 (Thermo Fisher Scientific)<br>For Real time PCR data acquisition: QuantStudio 7 Flex Real-Time PCR System (Applied Biosystems)
Data analysis	For WB image analysis: Image Lab v 6.1. (Bio-Rad)<br>For Nanoparticles analysis: Nanoparticle Tracking Analysis software v 3.4 (Malvern Panalytical)<br>For EV diameter and other immunofluorescence quantification: FIJI v 2.16.0/1.54p (ImageJ2) and Harmony Software v 5.1 (Revvity)

For FACS data analysis: FlowJo v 10.8.0 (FlowJo, LLC)
For statistical analysis and graphs: Graph Pad Prism version 10.6.1 (Graph Pad software, San Diego, CA)
For mass spectometry: Proteome Discoverer v 2.5.0.400 (Thermo Fisher Scientific), ComplexHeatmap v2.15.4, R v 4.3.1., limma v 3.56.2 package, DEqMS v 1.18.0.
For figure: Adobe Illustrator v 30.4 (Adobe)

For manuscripts utilizing custom algorithms or software that are central to the research but not yet described in published literature, software must be made available to editors and reviewers. We strongly encourage code deposition in a community repository (e.g. GitHub). See the Nature Portfolio guidelines for submitting code & software for further information.

Data

Policy information about availability of data

All manuscripts must include a data availability statement. This statement should provide the following information, where applicable:

- Accession codes, unique identifiers, or web links for publicly available datasets
- A description of any restrictions on data availability
- For clinical datasets or third party data, please ensure that the statement adheres to our policy

All the Mass spectrometry proteomics data are publicly available as of the date of publication and have been deposited to Massive with the accession number MSV000101801. The data are private but can be accessed by reviewers at: https://doi.org/doi:10.25345/C5FB4X13K, using the password: vQ1Zzj4zioo7l1SP
All the Mass spectrometry lipidomics data are publicly available as of the date of publication and have been deposited to Massive with the accession number MSV000101819.The data are private but can be accessed by reviewers at: https://doi.org/doi:10.25345/C53X84089 and using the password: BDL3NoO8605jWXqg
Source data along with uncropped blots are provided with this paper.All data and materials generated in this study are available from the corresponding author upon reasonable request.

Research involving human participants, their data, or biological material

Policy information about studies with human participants or human data. See also policy information about sex, gender (identity/presentation), and sexual orientation and race, ethnicity and racism.

Reporting on sex and gender	The study did not involve human participants.
Reporting on race, ethnicity, or other socially relevant groupings	The study did not involve human participants.
Population characteristics	The study did not involve human participants.
Recruitment	The study did not involve human participants.
Ethics oversight	The study did not involve human participants.

Note that full information on the approval of the study protocol must also be provided in the manuscript.

Field-specific reporting

Please select the one below that is the best fit for your research. If you are not sure, read the appropriate sections before making your selection.

☒ Life sciences ☐ Behavioural & social sciences ☐ Ecological, evolutionary & environmental sciences

For a reference copy of the document with all sections, see nature.com/documents/nr-reporting-summary-flat.pdf

Life sciences study design

All studies must disclose on these points even when the disclosure is negative.

Sample size	Sample size was not predetermined. The sample size was determined by the kind of experiments conducted or the similar kind of experiments conducted previously by other groups or labs working in the field. For extracellular vesicles isolation, NTA analysis, and WB of EV proteins, at least 3 independent experimental replicates from different passage of cell lines was conducted.
Data exclusions	No data exclusions were made in this study. The results reflect the full dataset without any omissions or biases introduced by selective exclusion of data points. FACS sorting of SopF-GFP+ cells, where bulk sorting was conducted, very high GFP+ signals were excluded.
Replication	Where applicable, experiments were repeated multiple times using either identical or new sample sets, ensuring that all conditions were consistently included for relative comparison.<br>Where applicable, positive and negative controls were incorporated to ensure proper calibration of each experimental repeat and to verify the reproducibility of the results.<br>For all experiment, the exact sample size is provided in the legend to each figure.
Randomization	No randomization was carried out.
Blinding	Blinding was not carried out. Cells were prepared, plated, and handled by same investigator due to intricacy of experiments.

Reporting for specific materials, systems and methods

We require information from authors about some types of materials, experimental systems and methods used in many studies. Here, indicate whether each material, system or method listed is relevant to your study. If you are not sure if a list item applies to your research, read the appropriate section before selecting a response.

Materials & experimental systems

n/a	Involved in the study
☐	☒ Antibodies
☐	☒ Eukaryotic cell lines
☒	☐ Palaeontology and archaeology
☒	☐ Animals and other organisms
☒	☐ Clinical data
☒	☐ Dual use research of concern
☒	☐ Plants

Methods

n/a	Involved in the study
☒	☐ ChIP-seq
☐	☒ Flow cytometry
☒	☐ MRI-based neuroimaging

Antibodies

Antibodies used

Primary antibodies used in the study:
GFP-HRP (Immunoblotting- 1:5,000, Miltenyi Biotech, 130091833)
ATG7 (Immunoblotting 1:1,000, Cell Signaling Technology, 8558)
ATG9A (Immunoblotting 1:1,000; Immunofluorescence 1:500; Abcam, ab108338)
ATG2A (Immunoblotting 1:500, Proteintech, 2322-1-AP)
ATG2B (Immunoblotting 1:500, Proteintech, 25155-1-AP)
GAPDH-HRP (Immunoblotting 1:1,000, Santa Cruz Biotechnology, sc-47724)
LC3B (Immunoblotting 1:1,000; Immunofluorescence 1:100; Cell Signaling Technology, 3868)
CD63 (Immunoblotting 1:1,000; Immunofluorescence 1:500; BD Bioscience, 556019)
Calnexin (Immunoblotting 1:1,000, Cell Signaling Technology, 2679)
CD63-APC (FACS 1:100, BD Biosciences, 561983)
PLIN3/TIP47 (Immunofluorescence 1:500; Proteintech, 10694-1-AP)
Rab27a (Immunoblotting 1:1000, Cell Signaling Technology, 69295)
TGN46 (Immunofluorescence 1:500, Bio-Rad, AHP500G)
Ceramide (IImmunofluorescence 1:500, Sigma, C8104)
TAX1BP1 (Immunoblotting 1:1,000, Immunofluorescence 1:500; Bethyl, A303-791AT)
SQSTM1 (Immunoblotting 1:1,000; Immunofluorescence 1:500; Progen, GP62-C)
FTH1 (Immunoblotting 1:1,000; Immunofluorescence 1:500; Cell Signaling Technology, 4393T)
FIP200 (Immunoblotting 1:1000; Cell Signaling Technology, 12436)
Secondary antibodies used in study:
Star Red anti-rabbit (STED 1:500, Abberior; STRED-1002)
Star Orange anti-mouse (STED 1:500, Abberior; STRED-1001)
HRP-conjugated anti-guinea pig IgG (Immunoblotting 1:5,000, Jackson Immuno Research, 706-035-148)
HRP-conjugated anti-rabbit IgG (Immunoblotting 1:10,000, Amersham, NA934V)
and HRP-conjugated anti-mouse IgG (Immunoblotting 1:10,000, Jackson Immuno Research, 115-035-174).

Validation

All antibodies are from commercially available sources and have been validated by the manufacturer with supporting publications found on manufacturer websites. All immunostaining include a "no primary antibody"; no signal was observed in any of the negative controls confirming the specific binding of the primary antibodies to their targets. All antibodies showed the bands of expected molecular weights.

Eukaryotic cell lines

Policy information about cell lines and Sex and Gender in Research

Cell line source(s)	HeLa (ATCC, CCL-2, LOT:70037076) cells was used in this study.
Authentication	1. New cell lines from ATCC were not authenticated. 2. Specific KO mutations were made in HeLa cells and was confirmed via western blotting using the corresponding antibodies.
Mycoplasma contamination	The cell lines were not tested for mycoplasma contamination. ATCC, cell line source mentions that all distributed cell lines are mycoplasm free. DAPI-staining did not show any signs of mycoplasma contamination througout the study.
Commonly misidentified lines (See ICLAC register)	No commonly misidentified lines were used in this study.

Plants

Seed stocks	The study did not involve plant samples.
Novel plant genotypes	The study did not involve plant samples.
Authentication	The study did not involve plant samples.

Flow Cytometry

Plots

Confirm that:

- [x] The axis labels state the marker and fluorochrome used (e.g. CD4-FITC).
- [x] The axis scales are clearly visible. Include numbers along axes only for bottom left plot of group (a 'group' is an analysis of identical markers).
- [x] All plots are contour plots with outliers or pseudocolor plots.
- [x] A numerical value for number of cells or percentage (with statistics) is provided.

Methodology

Sample preparation	For single cell sort or bulk sort, cells were washed, harvested with trypsin and were resuspended in complete DMEM. Cells were strained through 35micron nylon strainer cap to polystyrene FACS tubes, which were then sorted.<br>For FACS analysis-Cells were harvested using PBS-EDTA, washed with PBS, and 5 x10^5 cells were suspended in FACS tubes. Cells were washed with FACS buffer (1% FBS in PBS) and incubated with primary labelled antibodies in FACS buffer for 1 h at 4°C, as previously described108. After three washes cells were resuspended in PBS and 10,000 events were acquired on a LSRFortessa Cell Analyzer (BD Bioscience) and analyzed using FlowJo Software version 10.8.0 (FlowJo, LLC).
Instrument	Cells were sorted according to their characteristic GFP fluorescence using acBD FACSAriaII cell sorter (Becton Dickinson Biosciences) equipped with a 100-micron nozzle. FACS analysis was carried out on LSRFortessa flow cytometer (BD Bioscience)
Software	For FACS data acquisition: FACSDiVa v 8.0.1. (BD biosciences)<br>For FACS data analysis: FlowJo v 10.9.0 (BD biosciences)
Cell population abundance	All the analysis was performed on 10,000 cells/sample.
Gating strategy	Forward (FSC) and side scatter (SSC) was utilized to mark viable cell population. Unlabelled HeLa cells were used for setting the gate for background signal in any particular channel.

- [x] Tick this box to confirm that a figure exemplifying the gating strategy is provided in the Supplementary Information.
