# Supplementary material for: Lipid-manager autophagy proteins ATG2 and ATG9 regulate extracellular vesicle secretion via amphisome biogenesis and cell lipidome modulation
Source: Nat Commun. 2026 Jul 22;17:8948. doi: 10.1038/s41467-026-75742-x (PMC13503743; doi:10.1038/s41467-026-75742-x)
Supplement: Supplementary file 4 [file 41467_2026_75742_MOESM9_ESM.pdf]

nature portfolio

Peer Review File

Lipid-manager Autophagy Proteins ATG2 and ATG9 Regulate Extracellular Vesicles Secretion via Amphisome Biogenesis and Cell Lipidome Modulation

Corresponding Author: Dr Carlos Guardia

This file contains all reviewer reports in order by version, followed by all author rebuttals in order by version.

Version 0:

Reviewer comments:

Reviewer #1

(Remarks to the Author)
In this manuscript, Singh et al. employ multiple complementary approaches to investigate how crosstalk between the endosomal pathway and autophagy contributes to exosome biogenesis. Specifically, they show that depletion of the autophagosome biogenesis proteins ATG9A and ATG2A/B blocks amphisome formation and leads to increased secretion of CD-63 positive small extracellular vesicles (sEVs), particularly under conditions where lysosome activity is inhibited. Further, ATG9/ATG2 depletion alters the cellular lipidome, promoting sphingomyelin and ceramide accumulation which likely augments intraluminal vesicle production ahead of sEV secretion. Overall, this work will be of broad interest to the autophagy, extracellular vesicle, and membrane trafficking fields.
The experiments are well designed and the data is largely convincing. The evidence for a ceramide-dependent mechanism is consistent with the literature and backed up by small molecule inhibition (GW4869), but how this ILV route is ordinarily triggered in the absence of BafA1-dependent lysosome inhibition is not yet fully developed. I have no major critiques, but as the interpretation of cargo, protein and lipid composition rerouting critically depends on the specificity and quality of the EV preparations, I would recommend more controls be considered for that data. Second, while the GW4869 experiments support a role for ceramide-dependent EV biogenesis in autophagy-deficient cells, the persistently elevated CD63-positive EV secretion in ATG2 DKO cells indicate that additional mechanisms may contribute and warrant further quantitative analysis and discussion. Addressing these issues would strengthen the mechanistic conclusions and increase confidence in the proposed model.
1. Exosome purity: The authors do a very nice job of tracking CD63 partitioning into the EV fraction, but there is relatively little biochemical evaluation of the overall purity of that fraction. To establish the proportion of vesicles isolated by SEC as sEVs, the authors might consider demonstrating both positive and negative markers of EVs to rule out major cellular and non-vesicular contaminants. Related, to what extent do the SEC-isolated sEVs derive from a CD63 origin, consistent with MVE related biogenesis? To better infer the biological origin, the authors might consider additional markers associated with alternative EV sources, particularly plasma membrane-derived small ectosomes. Similarly, it may be informative to highlight the relative amounts of these proteins in the mass spec analysis under different ATG KO conditions (figure 4).
2. Figure 1b and extended Figure 1b:
The separate traces are difficult to see in these figures. The authors might consider layouts where the curves are not directly superimposed. Furthermore, the colors used (especially for WT and ATG7 KO) should be adjusted to improve contrast and visual distinction.
3. Figure 1h–k:
Consider combining panels (h) and (j) as well as (i) and (k) to facilitate clearer comparison between control medium (CM) and Bafilomycin A1 conditions.
4. Line 117:
Typographical error: “ampishomes” should be corrected to “amphisomes.”
5. Lines 161 and 163:
Please clarify the figure reference.
6. Figure 3b and 3c:
The authors’ model predicts both more fusion events (reflecting the blocked amphisome maturation) and increased sEVs per fusion event (reflecting the apparent increased number of ILVs in the EM). If so, the authors should consider quantifying fluorescent intensity of each fusion event in addition to the total fusion events per cell.

7. Figure 4c/d: Broadly speaking, the deletion of the lipid handling proteins increases sEV secretion, while the deletion of any of the autophagy proteins decreases the packaging of autophagy cargo receptors. Ferritin heavy chain looks like an exception though, as its packaging into sEVs in CM appears to specifically increase in ATG9/2 depletions. Can the authors discuss this apparent activity? As microautophagy has also been described to control Ferritin levels, perhaps the increased ILV production they observe in some depletion experiments could be a contributing factor.
8. Figure 5h:
The relationship between ATG7 KO–induced ceramide changes and the bar graph showing ceramide MFI in the TGN requires clarification, as ATG7 KO image appears to show higher TGN ceramide levels than WT.
9. Figure 5 and associated text:
The figure panels and call-outs of these panels in the main text do not fully align. Please recheck each figure call-out.
10. Extended Data Figure 5.1b:
Please verify the labelling of Western blot lanes, particularly lane 2 (ATG7 KO?) and lane 3 (ATG9A KO?).
11. Figure 6g:
In this summary under ATG9 KO and ATG2 DKO conditions, the authors should clarify why they still consider the possibility of CASM-derived LC3B-II in sEVs, which they appear to have ruled out by the experiments in figure 2a and extended figure 2h. In particular, LC3-II accumulates in small vesicles in ATG2 KO cells (as discussed by authors), and partitions separately from CD63 (figure 2i), suggesting there may be very little protein available to support a CASM-dependent formation of CD63 exosomes implying that this process is LC3-II independent.

Reviewer #2

(Remarks to the Author)
I co-reviewed this manuscript with one of the reviewers who provided the listed reports. This is part of the Nature Communications initiative to facilitate training in peer review and to provide appropriate recognition for Early Career Researchers who co-review manuscripts.

Reviewer #3

(Remarks to the Author)
Overall comment:
This study presents compelling cellular, proteomic, and lipidomic evidence that loss of ATG9A or ATG2A/B leads to increased small EV secretion and altered endosomal lipid composition, a phenotype that is clearly distinct from canonical autophagy impairment, as evidenced by the lack of a similar effect in ATG7-deficient cells. While these findings strongly support a role for ATG9A/ATG2 in restraining endosome-derived EV output, the mechanistic and physiological interpretation warrants careful framing. The key phenotypes described—particularly enhanced EV secretion and release of LC3-positive vesicles under lysosomal inhibition—are revealed primarily under chronic genetic ablation or pharmacological blockade of lysosomal function, conditions that likely represent non-physiological stress states rather than regulated trafficking decisions. Thus, although the data convincingly demonstrate that ATG9A/ATG2 are required to prevent lipid-driven rerouting of membranes into the exosome pathway, they do not yet establish whether this restraint constitutes a dynamically regulated physiological function or instead reflects a buffering role that becomes apparent only when membrane homeostasis is severely perturbed. Clarifying whether partial, acute, or context-dependent modulation of ATG9A/ATG2 activity can tune EV output under more physiological conditions would substantially strengthen the model and help distinguish normal membrane-partitioning mechanisms from stress-induced overflow phenomena. However, the reviewer recognizes that such experiments are technically challenging at present. Overall, the data are robust, internally consistent, and support the authors' main conclusions. Even with the limitation noted above, the results will be valuable to the community for understanding how lipid transfer is mediated in cells—a complex yet important topic in cell biology.

Minor points:
1. The reviewer feels that citing Tsuboyama et al., Science (2016) and briefly explaining that ATG7 KO does not completely prevent autophagosome formation would help a broader audience better understand the comparisons among the various ATG KO cell lines used in this study.

2. The sentence describing ATG7 KO cells implies that inhibition of LC3 lipidation is demonstrated in Fig. 1a; however, Fig. 1a appears to show only loss of ATG7 expression. Please clarify the wording or provide experimental evidence supporting inhibition of LC3 lipidation (perhaps shown in Extended Data Fig. 1a).

3. Lines 159–161 state: "Cells treated with BafA1 showed a significant increase of amphisomal structures in the WT and ATG7 KO cells, where different cellular content in large endosomes can be observed (Fig. 1h,i)." This feature is difficult to discern for untrained readers. Please guide readers more explicitly to the defining ultrastructural features in these micrographs.

4. Figures 1j and 1k do not appear to be referenced in the text. In addition, the text seems to contain incorrect figure references in lines 159–163. Please verify the citations to Fig. 1f–k, particularly (Fig. 1f,g) in line 159 and (Fig. 1h,i) in lines 161 and 163.

5. Please explain what the dotted-line circles in Fig. 2i represent and how they were defined.

6. The sentence "In none of the conditions analyzed for these KO cells, these LC3-positive aggregates mixed with CD63-

positive MVEs (Fig. 2i), suggesting that amphisomes formation is effectively disrupted in these autophagosome-deficient cells." is confusing, as the term "mixed" is imprecise. Rephrasing this description would improve clarity.

7. The reference (Fig. 3a,b) in line 176 likely should read (Fig. 2a,b).

Reviewer #4

(Remarks to the Author)
Comments on Singh et al.
Cells secrete small vesicles (exosomes or sEVs) into the extracellular space and sequester cellular material in autophagosomes. Both pathways intersect in the endosomal compartment, where intralumenal small vesicles and larger vesicles derived from the inner autophagosomal membrane can be present in the same population of endosomes.
Here, the authors investigate how the autophagy lipid transfer and scrambling proteins ATG2 and ATG9, as well as the ATG7 protein involved in LC3 lipidation, influence exosome secretion and composition and the cellular lipidome. The authors report a number of interesting observations regarding the composition of exosomes in cells lacking the proteins mentioned above. The main findings are a drastically changed cellular lipidome and an increase in exosome secretion on cells lacking ATG2A/B or ATG9A. In addition, it is reported that, as expected, the secretion of autophagosomal cargoes is abolished in these cells.
Overall, the manuscript reports several observations, which are useful for the community. However, it is unclear if many of the differences between wild-type and ATG-deficient cells are due to direct autophagy-related effects of ATG2/9, chronic autophagy inhibition, and therefore indirect effects and/or autophagy-independent functions. Therefore, the actual impact of this study is somewhat limited in its current form.
Major comments:
1. ATG2A/B and ATG9A are essential autophagy proteins, and their deletion almost completely blocks autophagosome formation. However, it is becoming clear that these proteins have autophagy-independent roles. For example, ATG2 functions in lysosome repair (PMID: 36071159) and on lipid droplets (PMID: 41249819), while ATG9 cycles in the endosomal compartment and has been reported to have a role in plasma membrane integrity (PMID: 34257406). It is therefore far from clear if the effects on exosome secretion and the lipidome can be attributed (solely) to a lack of autophagosome biogenesis.
Further, the effects of ATG2A/B and ATG9A deletion are compared to ATG7 deletion. Deletion of ATG7 abolishes ATG8 lipidation in autophagy and CASM. While ATG8 lipidation is important for autophagosome biogenesis, this process is not essential (PMID: 27885029, 27864321). It is therefore not clear if the effects seen in the ATG2/AB and ATG9A cells are due to their lipid handling activities during autophagosome biogenesis or chronic, total block of macroautophagy.
In combination, the convoluted effects mentioned above make the reported observation hard to reconcile into a clear take-home message. Most of these issues could potentially be clarified by comparing the ATG2A/B and ATG9 KO cells with FIP200 KO cells. FIP200 deletion also completely blocks autophagosome biogenesis but it is not directly involved in lipid handling. Also, the authors could acutely deplete the ATG2A/B proteins using AID-based approaches.
2. To rule out the role of CASM in the formation of LC3-rich sEVs (lines 202-207), the authors could express the bacterial effector SopF (PMID: 31327526), which specifically blocks this pathway.
3. Figure 2i: the authors should clarify how the boundaries of amphisomes were defined.
4. Figure 4b: the authors should clarify which samples/conditions were compared for the enrichment analysis.
5. Lines 339, 349; Fig. 5a: the authors conclude that a small but significant increase in the PC/PE ratio was observed only in the ATG2AB DKO cells. However, it seems as if the same effect would also be observed for the other KO lines if the number of experiments/data points were increased.

Minor:
Line 62: the "autophagy initiation complex" should be renamed to ULK1/2 complex, if this is what they mean.
Figure 1j, k: these figure panels don't seem to be referred to in the text.
Line 176: it should be Fig. 2a,b instead of Fig. 3
Lines 318 – 322: This sentence should be rephrased because ATG7 is also involved in autophagosome formation.
Line 350: it should be Fig. 5h and not f

Version 1:

Reviewer comments:

Reviewer #1

(Remarks to the Author)
I am very satisfied with the revisions intended to address our concerns. I appreciate the new data and also the modified interpretations of their model.

Reviewer #2

(Remarks to the Author)
I co-reviewed this manuscript with one of the reviewers who provided the listed reports. This is part of the Nature

Communications initiative to facilitate training in peer review and to provide appropriate recognition for Early Career Researchers who co-review manuscripts.

Reviewer #3

(Remarks to the Author)
The revised manuscript has adequately addressed my concerns.

Reviewer #4

(Remarks to the Author)
The authors have addressed all my concerns and I have no further comments.

Open Access This Peer Review File is licensed under a Creative Commons Attribution 4.0 International License, which permits use, sharing, adaptation, distribution and reproduction in any medium or format, as long as you give appropriate credit to the original author(s) and the source, provide a link to the Creative Commons license, and indicate if changes were made.
In cases where reviewers are anonymous, credit should be given to 'Anonymous Referee' and the source.
The images or other third party material in this Peer Review File are included in the article's Creative Commons license, unless indicated otherwise in a credit line to the material. If material is not included in the article's Creative Commons license and your intended use is not permitted by statutory regulation or exceeds the permitted use, you will need to obtain permission directly from the copyright holder.
To view a copy of this license, visit https://creativecommons.org/licenses/by/4.0/

Responses to Reviewers

We thank the editor and reviewers for their careful assessment of our manuscript and for the constructive recommendations. We have revised the manuscript, figures, figure legends, and Methods to address the reviewers' concerns. In particular, we strengthened the biochemical characterization of the EV preparations, clarified the relationship between ATG9A/ATG2A/B function, lipid remodeling, amphisome biogenesis, and MVE-derived secretion, generated several new lines to answer concerns from the reviewers, revised the discussion to avoid overinterpreting chronic knockout and lysosome-inhibition conditions as physiological regulatory states, and corrected figure call-outs and typographical errors. We provide point-by-point responses below.

Reviewer #1:

In this manuscript, Singh et al. employ multiple complementary approaches to investigate how crosstalk between the endosomal pathway and autophagy contributes to exosome biogenesis. Specifically, they show that depletion of the autophagosome biogenesis proteins ATG9A and ATG2A/B blocks amphisome formation and leads to increased secretion of CD-63 positive small extracellular vesicles (sEVs), particularly under conditions where lysosome activity is inhibited. Further, ATG9/ATG2 depletion alters the cellular lipidome, promoting sphingomyelin and ceramide accumulation which likely augments intraluminal vesicle production ahead of sEV secretion. Overall, this work will be of broad interest to the autophagy, extracellular vesicle, and membrane trafficking fields. The experiments are well designed and the data is largely convincing.

We thank this reviewer for the positive assessment, finding our manuscript "of broad interest" to several fields and for acknowledging that "experiments are well-designed and the data is largely convincing".

The evidence for a ceramide-dependent mechanism is consistent with the literature and backed up by small molecule inhibition (GW4869), but how this ILV route is ordinarily triggered in the absence of BafA1-dependent lysosome inhibition is not yet fully developed. I have no major critiques, but as the interpretation of cargo, protein and lipid composition rerouting critically depends on the specificity and quality of the EV preparations, I would recommend more controls be considered for that data. Second, while the GW4869 experiments support a role for ceramide-dependent EV biogenesis in autophagy-deficient cells, the persistently elevated CD63-positive EV secretion in ATG2 DKO cells indicate that additional mechanisms may contribute and warrant further quantitative analysis and discussion. Addressing these issues would strengthen the mechanistic conclusions and increase confidence in the proposed model.

1. Exosome purity: The authors do a very nice job of tracking CD63 partitioning into the EV fraction, but there is relatively little biochemical evaluation of the overall purity of that fraction. To establish the proportion of vesicles isolated by SEC as sEVs, the authors might consider demonstrating both positive and

negative markers of EVs to rule out major cellular and non-vesicular contaminants. Related, to what extent do the SEC-isolated sEVs derive from a CD63 origin, consistent with MVE related biogenesis? To better infer the biological origin, the authors might consider additional markers associated with alternative EV sources, particularly plasma membrane-derived small ectosomes. Similarly, it may be informative to highlight the relative amounts of these proteins in the mass spec analysis under different ATG KO conditions (figure 4).

We have expanded the characterization of the EV fractions and now include additional positive and negative controls for the isolated vesicle preparations (Extended Figure 2.1, panels a and b). Specifically, the revised manuscript includes immunoblot-based assessment of canonical sEV-associated proteins, including CD63 and syntenin-1, together with negative cellular-contaminant markers such as calnexin. To address the possibility that the fractions contain plasma-membrane-derived small ectosomes, we also evaluated ectosome-associated/plasma-membrane-enriched markers, including CD98 (SLC3A2) and CD147 (BSG), and we now discuss these data in the context of CD63-positive, MVE-derived vesicle enrichment rather than absolute purity. In the proteomics section, we have clarified that equal protein input was used for the mass-spectrometry analysis (30 µg of total protein per EV sample) and we now point readers to the corresponding proteomics/EV-marker summary so the relative abundance of these markers can be evaluated across WT and ATG-deficient conditions (Extended Figure 4.1b). However, given that we did not normalize these samples per number of EVs, the results from the immunoblots and proteomics are not directly comparable.

2. Figure 1b and extended Figure 1b: The separate traces are difficult to see in these figures. The authors might consider layouts where the curves are not directly superimposed. Furthermore, the colors used (especially for WT and ATG7 KO) should be adjusted to improve contrast and visual distinction.

We have revised these panels (revised Figure 1b and Extended Data Fig. 1b). The NTA traces are now presented in a clearer layout without the dispersion traces, and the color palette has been adjusted to provide better contrast between the different samples.

3. Figure 1h–k: Consider combining panels (h) and (j) as well as (i) and (k) to facilitate clearer comparison between control medium (CM) and Bafilomycin A1 conditions.

We have reorganized the panels as suggested (now revised Figure 1h and i). The control-medium and BafA1 conditions are now displayed together for the corresponding ultrastructural measurements, enabling direct comparison between basal and lysosome-inhibited states.

4. Line 117: Typographical error: "ampishomes" should be corrected to "amphisomes."

Corrected.

5. Lines 161 and 163: Please clarify the figure reference.

We have checked and corrected the figure reference.

6. Figure 3b and 3c: The authors' model predicts both more fusion events (reflecting the blocked amphisome maturation) and increased sEVs per fusion event (reflecting the apparent increased number of ILVs in the EM). If so, the authors should consider quantifying fluorescent intensity of each fusion event in addition to the total fusion events per cell.

We agree that event intensity provides important information beyond event frequency, so we have added quantification of pHluorin-CD63 fluorescence intensity per fusion event, using the same live-cell TIRF imaging framework. The revised Figure 3 now separates two related parameters: the number of CD63-positive MVE fusion events per cell (Figure 3c) and the mean fluorescence intensity associated with individual fusion events (new Figure 3d). These analyses strengthen the interpretation that ATG2A/B-deficient cells display enhanced MVE-plasma membrane fusion and that the released CD63-positive structures are consistent with increased ILV/MVE cargo burden.

7. Figure 4c/d: Broadly speaking, the deletion of the lipid handling proteins increases sEV secretion, while the deletion of any of the autophagy proteins decreases the packaging of autophagy cargo receptors. Ferritin heavy chain looks like an exception though, as its packaging into sEVs in CM appears to specifically increase in ATG9/2 depletions. Can the authors discuss this apparent activity? As microautophagy has also been described to control Ferritin levels, perhaps the increased ILV production they observe in some depletion experiments could be a contributing factor.

We thank the reviewer for this insight. We now note that ferritin can be secreted through both nonclassical secretory autophagy and MVE-exosome pathways as shown before (PMID: 29074498 and 34700146, new Lines 335-338), and that the elevated basal FTH1 signal in ATG9A KO and ATG2AB DKO sEVs may reflect enhanced ILV production or microautophagy-like ferritin handling in these backgrounds.

8. Figure 5h: The relationship between ATG7 KO–induced ceramide changes and the bar graph showing ceramide MFI in the TGN requires clarification, as ATG7 KO image appears to show higher TGN ceramide levels than WT.

We agree that this needed clarification. We have revised the figure since ATG7 KO cells can show cytoplasmic/background ceramide signal that is visually prominent but does

not necessarily correspond to increased TGN-localized ceramide signal after masking or a good representation of the whole population of cells.

9. Figure 5 and associated text: The figure panels and call-outs of these panels in the main text do not fully align. Please recheck each figure call-out.

We have rechecked and corrected the Figure 5 call-outs throughout the Results section.

10. Extended Data Figure 5.1b: Please verify the labelling of Western blot lanes, particularly lane 2 (ATG7 KO?) and lane 3 (ATG9A KO?).

We thank the reviewer for catching this labeling error. The lane labels have been corrected.

11. Figure 6g: In this summary under ATG9 KO and ATG2 DKO conditions, the authors should clarify why they still consider the possibility of CASM-derived LC3B-II in sEVs, which they appear to have ruled out by the experiments in figure 2a and extended figure 2h. In particular, LC3-II accumulates in small vesicles in ATG2 KO cells (as discussed by authors), and partitions separately from CD63 (figure 2i), suggesting there may be very little protein available to support a CASM-dependent formation of CD63 exosomes implying that this process is LC3-II independent.

After performing the experiments suggested by Reviewer #4 (overexpression of SopF, a CASM inhibitor, followed by isolation of sEVs and characterization of LC3-II levels), we can now conclude that stable GFP-SopF expression did not lead to a clear decrease in LC3-II levels in sEVs, suggesting that CASM is unlikely the primary source of residual LC3-II in ATG9A and ATG2AB DKO derived sEVs (Extended data Figure 2.1). Accordingly, we have revised the model (Figure 6f,g) and text to avoid presenting CASM as a likely source of $CD63^{+}LC3B^{+}$ sEVs in BafA1-treated ATG9A KO or ATG2AB DKO cells. This is in line with the results from the CQ experiments in now Extended Data Fig 2j, as this reviewer nicely pointed out. Further experiments will be needed to properly characterize then how LC3-II is present in the sEVs from these KO cells.

Reviewer #2:

I co-reviewed this manuscript with one of the reviewers who provided the listed reports. This is part of the Nature Communications initiative to facilitate training in peer review and to provide appropriate recognition for Early Career Researchers who co-review manuscripts.

We thank the co-reviewer for their contribution to the peer-review process.

Reviewer #3

Overall comment: This study presents compelling cellular, proteomic, and lipidomic evidence that loss of ATG9A or ATG2A/B leads to increased small EV secretion and altered endosomal lipid composition, a phenotype that is clearly distinct from canonical autophagy impairment, as evidenced by the lack of a similar effect in ATG7-deficient cells. While these findings strongly support a role for ATG9A/ATG2 in restraining endosome-derived EV output, the mechanistic and physiological interpretation warrants careful framing. The key phenotypes described—particularly enhanced EV secretion and release of LC3-positive vesicles under lysosomal inhibition—are revealed primarily under chronic genetic ablation or pharmacological blockade of lysosomal function, conditions that likely represent non-physiological stress states rather than regulated trafficking decisions. Thus, although the data convincingly demonstrate that ATG9A/ATG2 are required to prevent lipid-driven rerouting of membranes into the exosome pathway, they do not yet establish whether this restraint constitutes a dynamically regulated physiological function or instead reflects a buffering role that becomes apparent only when membrane homeostasis is severely perturbed. Clarifying whether partial, acute, or context-dependent modulation of ATG9A/ATG2 activity can tune EV output under more physiological conditions would substantially strengthen the model and help distinguish normal membrane-partitioning mechanisms from stress-induced overflow phenomena. However, the reviewer recognizes that such experiments are technically challenging at present. Overall, the data are robust, internally consistent, and support the authors' main conclusions. Even with the limitation noted above, the results will be valuable to the community for understanding how lipid transfer is mediated in cells—a complex yet important topic in cell biology.

We thank the reviewer for this thoughtful and balanced assessment, highlighting that "the data are robust, internally consistent, and support the authors' main conclusions". We agree that the initial framing needs revision, so we have modified the Discussion accordingly to distinguish between regulated membrane partitioning and the stress/adaptation state crated by chronic ATG9 or ATG2 deletion and lysosomal inhibition.

Minor points:

1. The reviewer feels that citing Tsuboyama et al., Science (2016) and briefly explaining that ATG7 KO does not completely prevent autophagosome formation would help a broader audience better understand the comparisons among the various ATG KO cell lines used in this study.

We agree and have added this citation and explanatory text (new Line 143/4). The revised manuscript now clarifies that ATG7 KO blocks ATG8 lipidation and selective autophagy-related cargo handling but does not necessarily eliminate formation of all autophagosome-like structures. This distinction helps explain why ATG7 KO cells differ from ATG9A KO and ATG2AB DKO cells, which affect earlier lipid delivery/expansion steps in autophagosome biogenesis.

2. The sentence describing ATG7 KO cells implies that inhibition of LC3 lipidation is demonstrated in Fig. 1a; however, Fig. 1a appears to show only loss of ATG7 expression. Please clarify the wording or provide experimental evidence supporting inhibition of LC3 lipidation (perhaps shown in Extended Data Fig. 1a).

We have clarified the wording and corrected the figure reference. The revised text indicates that Fig. 1a confirms ATG7 loss, while the autophagic-flux/LC3 lipidation defect is supported by Extended Data Figure 1a.

3. Lines 159–161 state: "Cells treated with BafA1 showed a significant increase of amphisomal structures in the WT and ATG7 KO cells, where different cellular content in large endosomes can be observed (Fig. 1h,i)." This feature is difficult to discern for untrained readers. Please guide readers more explicitly to the defining ultrastructural features in these micrographs.

Based on this suggestion and since MVE structures are more relevant to the figure (focus is ILV quantification) than amphisomes (properly described later in the STED experiments), we decided to focus only on the clearly identifiable MVEs from the TEM images. Accordingly, we have revised the text and remove any reference to the other structures the reviewer points out.

4. Figures 1j and 1k do not appear to be referenced in the text. In addition, the text seems to contain incorrect figure references in lines 159–163. Please verify the citations to Fig. 1f–k, particularly (Fig. 1f,g) in line 159 and (Fig. 1h,i) in lines 161 and 163.

Thank you for pointing this out. We have now corrected these figure references accordingly.

5. Please explain what the dotted-line circles in Fig. 2i represent and how they were defined.

We have clarified this in the Fig. 2 legend and Methods and provided a new extended figure (Extended Data Figure 2.2) to facilitate the reader with the procedure we used to define such structures. Briefly, the dotted outlines indicate representative regions of interest drawn around visually identifiable LC3-positive structures and adjacent CD63-positive compartments under <u>low confocal resolution</u> before acquiring the same image using STED. Thus, they are not intended to define amphisome boundaries quantitatively. We have revised the legend to avoid implying that the dotted outlines were used as objective segmentation masks or as formal evidence of membrane continuity.

6. The sentence "In none of the conditions analyzed for these KO cells, these LC3-positive aggregates mixed with CD63-positive MVEs (Fig. 2i), suggesting that amphisomes formation is effectively disrupted in these

autophagosome-deficient cells." is confusing, as the term "mixed" is imprecise. Rephrasing this description would improve clarity.

We agree and have rephrased the sentence. The revised text now states that LC3-positive structures in ATG9A KO and ATG2AB DKO cells remain spatially segregated from CD63-positive compartments at the resolution of STED imaging, in contrast to the increased juxtaposition/overlap observed in BafA1-treated WT cells.

7. The reference (Fig. 3a,b) in line 176 likely should read (Fig. 2a,b).

Corrected.

Reviewer #4

Cells secrete small vesicles (exosomes or sEVs) into the extracellular space and sequester cellular material in autophagosomes. Both pathways intersect in the endosomal compartment, where intralumenal small vesicles and larger vesicles derived from the inner autophagosomal membrane can be present in the same population of endosomes. Here, the authors investigate how the autophagy lipid transfer and scrambling proteins ATG2 and ATG9, as well as the ATG7 protein involved in LC3 lipidation, influence exosome secretion and composition and the cellular lipidome. The authors report a number of interesting observations regarding the composition of exosomes in cells lacking the proteins mentioned above. The main findings are a drastically changed cellular lipidome and an increase in exosome secretion on cells lacking ATG2A/B or ATG9A. In addition, it is reported that, as expected, the secretion of autophagosomal cargoes is abolished in these cells. Overall, the manuscript reports several observations, which are useful for the community. However, it is unclear if many of the differences between wild-type and ATG-deficient cells are due to direct autophagy-related effects of ATG2/9, chronic autophagy inhibition, and therefore indirect effects and/or autophagy-independent functions. Therefore, the actual impact of this study is somewhat limited in its current form.

We thank the reviewer for this thoughtful critique. We agree that the original manuscript did not sufficiently separate three related issues: (i) loss of canonical autophagosome/amphisome biogenesis, (ii) chronic adaptation to knockout of ATG9A or ATG2A/B, and (iii) autophagy-independent functions of these lipid-handling proteins. We have revised the manuscript to make this distinction explicit, especially in the Introduction, Results, and Discussion sections.

Major comments:

1. ATG2A/B and ATG9A are essential autophagy proteins, and their deletion almost completely blocks autophagosome formation. However, it is becoming clear that these proteins have autophagy-independent roles. For example, ATG2 functions in lysosome repair (PMID: 36071159) and on lipid droplets (PMID: 41249819), while ATG9 cycles in the endosomal compartment and has been reported to have a role in plasma membrane integrity (PMID: 34257406). It is therefore far from clear if the effects on exosome secretion and the lipidome can be attributed (solely) to a lack of autophagosome biogenesis.

We agree with the reviewer that ATG9A and ATG2A/B have functions that extend beyond a simple block of canonical autophagy and we made an effort to reflect this more explicitly in the Introduction and Discussion sections.

Further, the effects of ATG2A/B and ATG9A deletion are compared to ATG7 deletion. Deletion of ATG7 abolishes ATG8 lipidation in autophagy and CASM. While ATG8 lipidation is important for autophagosome biogenesis, this process is not essential (PMID: 27885029, 27864321). It is therefore not clear if the effects seen in the ATG2/AB and ATG9A cells are due to their lipid handling activities during autophagosome biogenesis or chronic, total block of macroautophagy.

We agree and have revised the manuscript to clarify the purpose and limitation of the ATG7 KO comparison. ATG7 KO abolishes ATG8 lipidation, thereby disrupting ATG8-dependent cargo recruitment, CASM, and efficient autophagosome maturation, but it should not be interpreted as a complete autophagosome-null condition because ATG8

lipidation is not strictly required for all autophagosome-like structures. In contrast, ATG9A and ATG2A/B act earlier by supporting lipid delivery, membrane expansion, and phagophore maturation. Thus, the lack of a strong sEV and lipidome phenotype in ATG7 KO cells indicates that loss of ATG8 lipidation alone is insufficient to reproduce the phenotypes caused by ATG9A or ATG2A/B deletion; it does not prove that these phenotypes are independent of autophagy. We now interpret the ATG9A/ATG2A/B phenotypes more conservatively in the Discussion section, as potentially reflecting combined effects of impaired early autophagosome biogenesis, disrupted lipid-transfer/membrane-homeostasis functions, chronic adaptation to a severe autophagy-biogenesis block, and possible autophagy-independent activities of ATG9A/ATG2A/B.

In combination, the convoluted effects mentioned above make the reported observation hard to reconcile into a clear take-home message. Most of these issues could potentially be clarified by comparing the ATG2A/B and ATG9 KO cells with FIP200 KO cells. FIP200 deletion also completely blocks autophagosome biogenesis but it is not directly involved in lipid handling.

As suggested by the reviewer, we generated a FIP200 KO cells to compare the effects of impairing autophagy initiation with those caused by deletion of the lipid-transfer machinery (new Extended Data Figure2.3). In the revised manuscript, we now report that FIP200 KO cells do not exhibit enhanced EV secretion in terms of particle number but those sEVs have increased CD63 content. Similarly to ATG9A and ATG2AB DKO cells, EVs derived from FIP200 KO cells lack LC3, suggesting a defect in autophagosome and amphisome formation. Notably, unlike ATG9A and ATG2AB DKO cells, FIP200 KO cells don't show lipid accumulation, as assessed by PLIN3 staining for lipid droplets. These results show that while FIP200 loss mimics the EV cargo changes associated with autophagy disruption, the changes purely associated with ATG9A and ATG2AB DKO (enhanced EV secretion with smaller size ILVs and later-described whole-cell lipidome remodeling) are likely driven by the autophagy-independent roles in lipid handling of these proteins. This is now further examined in the Discussion section.

Also, the authors could acutely deplete the ATG2A/B proteins using AID-based approaches.

We appreciate the reviewer's thoughtful suggestion. We agree that an AID-based acute-depletion system would be an elegant approach to distinguish acute loss of ATG2A/B function from potential long-term adaptation in knockout cells. However, establishing this system in our model would require substantial new cell-line engineering (including endogenous tagging), validation of auxin-dependent degradation kinetics for both ATG2A and ATG2B and control ATG7 or ATG9A, and functional optimization. Unfortunately, this was not feasible within the timeframe available for revision and we niw address this as a limitation to our study in the Discussion section. As an alternative acute-perturbation strategy, we performed shRNA-mediated knockdown of the four different ATG genes in the study. Although we achieved detectable protein depletion within the first two passages post-transduction (see figure R1 below), protein knockdown was incomplete. Consistent with this partial reduction, we did not recapitulate the lipid droplet and p62 accumulation observed in the knockout cells, nor did we observe the robust increase in sEV secretion seen in the corresponding knockout lines. These results

suggest that partial reduction of ATG9A or ATG2A/B is not sufficient to reproduce the full phenotype observed after complete genetic deletion:

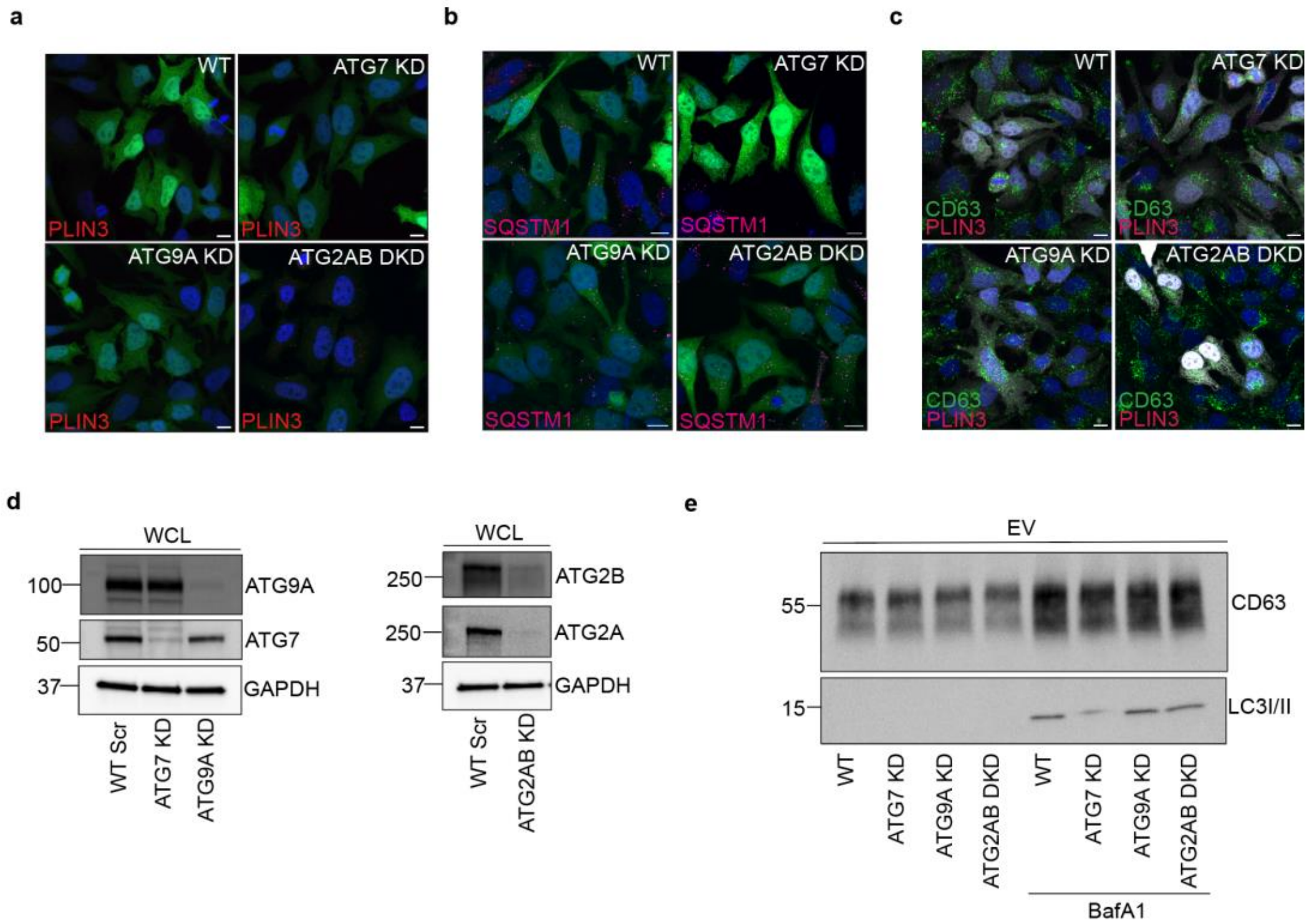

Figure R1: a-c, Representative confocal images of lipid droplets labeled with PLIN3/TIP47 (magenta) (a), p62/SQSTM1 (magenta) (b), and CD63 (green) (c) in WT cells expressing scrambled shRNA and ATG knockdown cells (shATG7, shATG9A, and shATG2A/B), all coexpressing GFP (part of the shRNA vector to demonstrate efficient transduction). Scale bar, 10 µm. **d,** Immunoblot analysis of the knockdown efficiency. **e,** Immunoblot analysis of the exosomal marker CD63 and the autophagy marker LC3B in sEVs isolated by size exclusion chromatography from either WT scrambled (Scr) and corresponding ATG knockdown cells in control medium or treated with 100 nM BafA1 for 16 h. Representative blots were probed with the indicated antibodies (right). Molecular weight markers are shown on the left.

2. To rule out the role of CASM in the formation of LC3-rich sEVs (lines 202-207), the authors could express the bacterial effector SopF (PMID: 31327526), which specifically blocks this pathway.

We agree that SopF provides a useful tool to test whether CASM contributes to the LC3-II-positive EV signal. To address this point, we generated stable GFP-SopF-expressing cells and analyzed the EV phenotype in the ATG2-deficient background. In the revised manuscript, we report now that stable GFP-SopF expression did not lead to a clear decrease in LC3-II levels in sEVs, suggesting that CASM is unlikely the primary source of residual LC3-II in ATG9A and ATG2AB DKO derived sEVs (Extended Data Figure 2.1k).

3. Figure 2i: the authors should clarify how the boundaries of amphisomes were defined.

We have clarified this in the Fig. 2 legend and Methods and provided a new extended figure (Extended Data Figure 2.2) to facilitate the reader with the procedure we used to define such structures. The dotted outlines indicate representative regions of interest drawn around visually identifiable LC3-positive structures and adjacent CD63-positive compartments under low confocal resolution before acquiring the same image using STED. Thus, they are not intended to define amphisome boundaries quantitatively. We have revised the legend to avoid implying that the dotted outlines were used as objective segmentation masks or as formal evidence of membrane continuity.

4. Figure 4b: the authors should clarify which samples/conditions were compared for the enrichment analysis.

We have clarified this in the figure and figure legend. The enrichment analysis in Fig. 4b was performed using the differentially detected EV proteins identified across the indicated WT versus ATG KO comparisons under control and BafA1-treated conditions.

5. Lines 339, 349; Fig. 5a: the authors conclude that a small but significant increase in the PC/PE ratio was observed only in the ATG2AB DKO cells. However, it seems as if the same effect would also be observed for the other KO lines if the number of experiments/data points were increased.

We agree with the reviewer and have removed the PC/PE ratio analysis from the main text and corresponding figures. Although the initial analysis suggested a possible trend, the number of replicates and the quantitative robustness of this derived ratio were not sufficient to support a strong mechanistic conclusion. We therefore revised the Results to focus on the lipid classes and lipidomic patterns that are better supported by the dataset, including sphingomyelin/ceramide remodeling, neutral-lipid accumulation, and changes in major glycerophospholipid species. We also removed the corresponding interpretation from the Discussion to avoid overstatement.

Minor:
Line 62: the "autophagy initiation complex" should be renamed to ULK1/2 complex, if this is what they mean.
Figure 1j, k: these figure panels don't seem to be referred to in the text.
Line 176: it should be Fig. 2a,b instead of Fig. 3
Lines 318 – 322: This sentence should be rephrased because ATG7 is also involved in autophagosome formation.
Line 350: it should be Fig. 5h and not f

We have made all these corrections.
